# Supplementary material for: How can community pharmacists be supported to manage skin conditions? A multistage stakeholder research prioritisation exercise
Source: BMJ Open. 2024 Jan 2;14(1):e071863. doi: 10.1136/bmjopen-2023-071863 (PMC10773317; doi:10.1136/bmjopen-2023-071863)
Supplement: Supplementary data [file bmjopen-2023-071863supp001.pdf]

Derm/Comm Pharm PSP Workshop 1 – Community Pharmacists

7pm WELCOME & INTRODUCTIONS FROM US.

- Thank-you for coming.
- Introductions from PL / JH.
- Purpose of the project.

The purpose of this project is to identify uncertainties that community pharmacists experience when they are faced with someone seeking help about a skin condition.

Our goal is to identify a small number of research projects which could be undertaken to support community pharmacists - these could be trialling new ways of working, developing information resources, understanding the challenges of seeing skin conditions in community pharmacy, testing treatments, etc.

- Purpose of this evening.

From the first stage of the project, an online survey of community pharmacists, we have identified several areas that we would like to understand further, outlined below.

- Rules of engagement.
- Recording start.

7.10pm AROUND PARTICIPANTS – INTRODUCTIONS.

- name, where from, pharmacist role/background.
- 1 thing about skin….

Is there one thing that you would like to know that would help you when dealing with a skin condition / skin condition query?

7.25pm YOUR SCORING OF THE MATERIALS SENT.

7.30pm TOPIC 1.

7.45pm TOPIC 2.

8.00pm SHORT BREAK.

8.10pm OTHER TOPICS AND QUESTIONS.

8.40pm RANKING/SCORING.

8.50pm. CLOSING COMMENTS AND THANKS.

- vouchers.
- final workshop date and possibilities.

Identifying and diagnosing skin disorders.

What's the biggest challenge in this // What would help? What do you need?

For example:
The need for resources to support skin condition identification.
Specific skin conditions that you find difficult to identify (moles, insect bites, general rashes).

Additional prompts:
[is it about skin conditions? Is it about facilities/resources? Is it about knowledge/resources]

What's the biggest challenge in this – lack of knowledge, anxious public, number of conditions.
Adults / children?
Mild and very early presentation?
Time? And facilities?
Existing resources – adequacy? Appropriateness?
Training.
What do you currently do – examples of good practice.

Knowing when to refer someone to a GP.

What's the biggest challenge in this // What would help? What do you need?

For example:
Knowing which conditions should be referred to a GP (or when the severity of a condition requires referral).
Understanding how to refer / the need for more formal mechanism of referral.
Knowing when it is ok to offer advice/product and manage locally.

Additional prompts:
[is it about skin conditions? Is it about facilities/resources? Is it about knowledge/resources]

Are there specific situations/conditions where this is a particular problem?
What would discourage you from referring a patient?
Would having a referral pathway be useful similar to those between GPs and secondary care?
Is the reason for referral due to not knowing the diagnosis or not being able to treat the condition once diagnosed?

Skin of Colour.

What's the biggest challenge in this // What would help? What do you need?

For example:
Understanding the different presentation of skin conditions in skin of colour.
Knowing skin conditions which are distinct / more prevalent in skin of colour.

Additional prompts:
[is it about skin conditions? Is it about facilities/resources? Is it about knowledge/resources]

Did you receive any training specifically in diagnosing/treating conditions in skin of colour?
How have you learnt about treating conditions in patients with skin of colour?
Are there any conditions that you see more frequently in patients with skin of colour?

Disease specific concerns.

What's the biggest challenge in this // What would help? What do you need?

For example:
Recognising specific conditions which can / cannot be managed in community pharmacy.
Understanding how to support specific, chronic skin conditions.
The potential for dedicated roles in supporting specific conditions – trained staff, dedicated services.
Supporting members of the public with eczema self-management.

Additional prompts:
[is it about skin conditions? Is it about facilities/resources? Is it about knowledge/resources]

What long-term conditions do you see the most frequently?
What conditions do you see patients struggle to deal with?

What conditions do think a pharmacist could help to manage?
What role do you currently have in managing long-term conditions?
What role would you like to have with managing conditions both acute and long-term?

Product specific concerns.

For example:
Understanding concerns about the use of topical corticosteroids.
Understanding how to navigate the range of products available for skin conditions.
Understanding the benefit of products for different conditions and different severities. Knowing conditions for which you have no treatment.

Additional prompts:
[is it about skin conditions? Is it about facilities/resources? Is it about knowledge/resources]

What other medications do you think should be available OTC? Could be tested as OTC?
What are the common problems you have when selling TCS OTC? How do you think these problems should be managed?
What conditions are the most difficult to treat with the products that you have available?
How do you think
