# Supplementary material for: How can community pharmacists be supported to manage skin conditions? A multistage stakeholder research prioritisation exercise
Source: BMJ Open. 2024 Jan 2;14(1):e071863. doi: 10.1136/bmjopen-2023-071863 (PMC10773317; doi:10.1136/bmjopen-2023-071863)
Supplement: Supplementary data [file bmjopen-2023-071863supp002.pdf]

Public Pharmacy Dermatology Priority Setting Workshop

7pm WELCOME & INTRODUCTIONS FROM US.

- Thank-you for coming.
- Introductions from PL / JH.
- Purpose of the project.

The purpose of this project is to identify uncertainties that community pharmacists experience when they are faced with someone seeking help about a skin condition.

Our goal is to identify a small number of research projects which could be undertaken to support community pharmacists - these could be trialling new ways of working, developing information resources, understanding the challenges of seeing skin conditions in community pharmacy, testing treatments, etc.

- Purpose of this evening.

This evening we want to talk about your experiences of using a community pharmacy and discuss what roles you think pharmacists can play in the management of skin conditions. We want to know what types of projects you think need to be done that could help pharmacies to offer the best possible care to their patients.

- Rules of engagement.
- Split into two groups.
- Recording start.

7.10pm AROUND PARTICIPANTS – INTRODUCTIONS.

- name, where from, do you have any skin conditions/ experience of having skin conditions,

7.20pm

- Have you ever consulted a pharmacist/member of pharmacy staff regarding a skin condition? What were your experiences of using community pharmacy to treat skin conditions? Would you be as happy to see a pharmacist about a skin condition as you would about a cold? Do you have a long-term community pharmacist? Why do you go to that pharmacist?
  - Positive
  - Negative

- What would encourage you to use a community pharmacy for a skin condition?
  - Access
  - lack of GP appointments?
  - Familiarity (do you know your local pharmacist?)
  - See skin condition as a minor condition and not worthwhile bothering a GP (seeking reassurance from pharmacist that they should see a GP)

- What would discourage you from using a community pharmacy
  - Privacy (how would you feel about going into the consultation room?)
  - Having to speak to counter staff (would you feel comfortable asking to speak to a pharmacist)
  - Worried about lack of knowledge (would you feel any better knowing that the pharmacist had accessed specialist training in treating skin conditions?)

- When you have been to a community pharmacy which members of staff have you spoken to? How often do they bring the pharmacist into the conversation? Do you always get to see the pharmacist if you ask? Do you trust what the counter assistant tells you? Would you trust the pharmacist more?

- For those of you who have had skin conditions or have cared for people with skin conditions, at what point in your treatment do you think a pharmacist could have improved the quality of the care you/they received from the NHS as a whole.

  Please think about:
  - The initial diagnosis
  - The first time you were prescribed a medication
  - Each time you picked up a prescription from the pharmacy
  - When you purchased a treatment for a skin condition from the pharmacy
  - If the pharmacist referred you to someone else for treatment

- What role do you think the pharmacist should have? Should they diagnose your skin condition? Should they just treat your skin condition?
- What do you know about pharmacists training?

8pm short break

8.10pm

8.10pm RESEARCH TOPICS AND QUESTIONS.

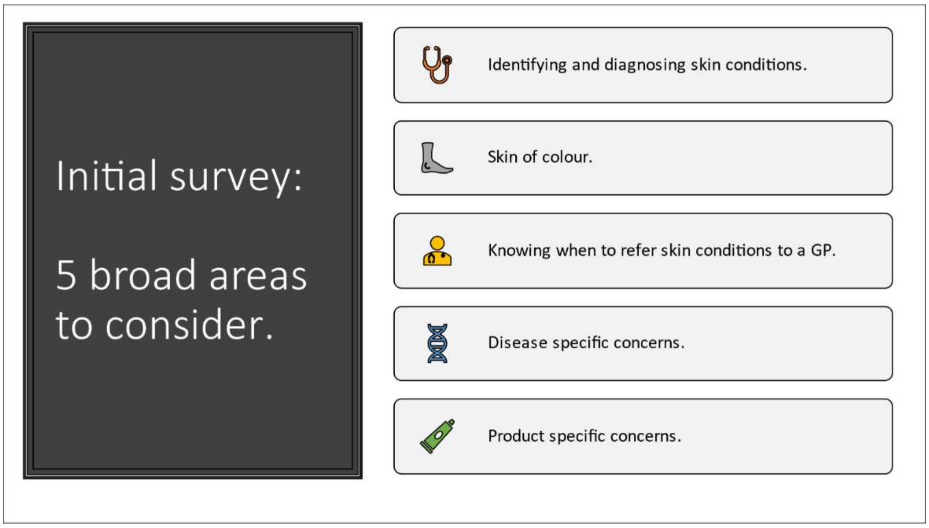

What do you think of this order?
Are you surprised?
Do you think the role of the pharmacist is to diagnose?
Or is their role just to tell you when you need to go to the GP?
Do you trust what a pharmacist says?
Do you think it would be useful if they could diagnose?

Identifying and diagnosing skin disorders.
What's the biggest challenge in this?
What would help? What do you need?

For example:
The need for resources to support skin condition identification.

Specific skin conditions that you find difficult to identify (moles, insect bites, general rashes).

Current use of photographs / websites / other resources?

What are you most likely to see? What is most likely to be difficult?

Additional prompts:
[is it about skin conditions? Is it about facilities/resources? Is it about knowledge/resources]

What's the biggest challenge in this – lack of knowledge, anxious public, number of conditions.
Adults / children?
Mild and very early presentation?
Time? And facilities?
Existing resources – adequacy? Appropriateness?
Training.
What do you currently do – examples of good practice.

Knowing when to refer someone to a GP.

What's the biggest challenge in this // What would help? What do you need?

For example:
Knowing which conditions should be referred to a GP (or when the severity of a condition requires referral).
Understanding how to refer / the need for more formal mechanism of referral.
Knowing when it is ok to offer advice/product and manage locally.

Knowing how long to wait?

What are you most likely to refer?

Additional prompts:
[is it about skin conditions? Is it about facilities/resources? Is it about knowledge/resources]

Are there specific situations/conditions where this is a particular problem?
What would discourage you from referring a patient?
Would having a referral pathway be useful similar to those between GPs and secondary care?
Is the reason for referral due to not knowing the diagnosis or not being able to treat the condition once diagnosed?

Disease specific concerns.

What's the biggest challenge in this // What would help? What do you need?

What conditions ? what challenges?

What's your role in this? What are you trying to achieve? [help GPs, …]

Do you want to do more?

Integrated services focused upon diseases?

For example:
Recognising specific conditions which can / cannot be managed in community pharmacy.
Understanding how to support specific, chronic skin conditions.
The potential for dedicated roles in supporting specific conditions – trained staff, dedicated services.
Supporting members of the public with eczema self-management.

Additional prompts:
[is it about skin conditions? Is it about facilities/resources? Is it about knowledge/resources]

What long-term conditions do you see the most frequently?
What conditions do you see patients struggle to deal with?
What conditions do think a pharmacist could help to manage?
What role do you currently have in managing long-term conditions?
What role would you like to have with managing conditions both acute and long-term?

Skin of Colour.

What's the biggest challenge in this // What would help? What do you need?

For example:
Understanding the different presentation of skin conditions in skin of colour.
Knowing skin conditions which are distinct / more prevalent in skin of colour.

Additional prompts:
[is it about skin conditions? Is it about facilities/resources? Is it about knowledge/resources]

Did you receive any training specifically in diagnosing/treating conditions in skin of colour?
How have you learnt about treating conditions in patients with skin of colour?
Are there any conditions that you see more frequently in patients with skin of colour?

Product specific concerns.

For example:
Understanding concerns about the use of topical corticosteroids.
Understanding how to navigate the range of products available for skin conditions.
Understanding the benefit of products for different conditions and different severities. Knowing conditions for which you have no treatment.

Additional prompts:
[is it about skin conditions? Is it about facilities/resources? Is it about knowledge/resources]

What other medications do you think should be available OTC? Could be tested as OTC?
What are the common problems you have when selling TCS OTC? How do you think these problems should be managed?
What conditions are the most difficult to treat with the products that you have available?
How do you think

8.40pm RANKING/SCORING.

8.50pm. CLOSING COMMENTS AND THANKS.
- vouchers.
- final workshop date and possibilities.
