# Supplementary material for: How can community pharmacists be supported to manage skin conditions? A multistage stakeholder research prioritisation exercise
Source: BMJ Open. 2024 Jan 2;14(1):e071863. doi: 10.1136/bmjopen-2023-071863 (PMC10773317; doi:10.1136/bmjopen-2023-071863)
Supplement: Supplementary data [file bmjopen-2023-071863supp003.pdf]

COREQ checklist

**Item number**		**Notes**
1	Which author/s conducted the interview or focus group?	All workshops and interviews were facilitated by both JH and PL.
2	What were the researcher's credentials? E.g. PhD, MD	JH has a Ph.D and MPharm. PL has a Ph.D
3	What was their occupation at the time of the study?	JH and PL work at the Centre of Evidence based Dermatology, School of Medicine, University of Nottingham.<br>JH is a registered pharmacist.
4	Was the researcher male or female?	N/A
5	What experience or training did the researcher have?	PL has extensive expertise in qualitative methods.<br><br>JH has completed several pieces of qualitative work examining pharmacist led services.
6	Was a relationship established prior to study commencement?	Researchers did not establish a relationship with participants prior to study commencement
7	What did the participants know about the researcher? e.g. personal goals, reasons for doing the research	Participants did not know that JH was a registered pharmacist. They were aware both researchers worked for the Centre of Evidence Dermatology.
8	What characteristics were reported about the inter viewer/facilitator? e.g. Bias, assumptions, reasons and interests in the research topic	We have acknowledged in the write-up that JH has a background as a pharmacist
9	What methodological orientation was stated to underpin the study? e.g. grounded theory, discourse analysis, ethnography, phenomenology, content analysis	This was exploratory, consultative research broadly informed by the James Lind Priority Setting Partnership approach.<br><br>See methods section, line 200, 206, 213, 216
10	How were participants selected? e.g. purposive, convenience, consecutive, snowball	See methods section line 165
11	How were participants approached? e.g. face-to-face, telephone, mail, email	Line 165,175
12	How many participants were in the study?	Table 1
13	How many people refused to participate or dropped out? Reasons?	Although the first focus groups were attended by 19 pharmacists, the final online consensus workshop was poorly attended by

		pharmacists and members of pharmacy staff. We had initially invited an equal number of pharmacists and pharmacy staff (12) and members of the public (13) but only 5/12 pharmacists and members of pharmacy staff, who were signed up prior to the meeting, attended the meeting.<br><br>Reasons (where provided) for nonattendance included sickness and that they were too busy.
14	Where was the data collected? e.g. home, clinic, workplace	Line 203
15	Was anyone else present besides the participants and researchers?	No
16	What are the important characteristics of the sample? e.g. demographic data, date	We did not collect any demographic data.
17	Were questions, prompts, guides provided by the authors? Was it pilot tested?	Workshops used the findings of earlier stages of the research as their focus.<br><br>Data analysis and progress summaries were considered by the wider author group and PPI representatives prior to the workshops.
18	Were repeat inter views carried out? If yes, how many?	N/A
19	Did the research use audio or visual recording to collect the data?	Line 211
20	Were field notes made during and/or after the interview or focus group?	No fieldnotes were made during or after data collection.
21	What was the duration of the inter views or focus group?	Line 250
22	Was data saturation discussed?	Data saturation was not a concept applied to this research, rather data collection
23	Were transcripts returned to participants for comment and/or correction?	No
24	How many data coders coded the data?	PL and JH coded the data. Other authors reviewed coded data and confirmed appropriateness of interpretations.
25	Did authors provide a description of the coding tree?	Yes Table 2

26	Were themes identified in advance or derived from the data?	Initial themes were derived from the inductive content analysis of the online survey. Themes were refined following the exploratory workshops.
27	What software, if applicable, was used to manage the data?	NVivo ver 12 was used to handle research data.
28	Did participants provide feedback on the findings?	No
29	Were participant quotations presented to illustrate the themes/findings? Was each quotation identified? e.g. participant number	Yes, identified by participant number and profession and by workshop number
30	Was there consistency between the data presented and the findings?	Yes findings were based on an analytic process that was iterative.
31	Were major themes clearly presented in the findings?	Yes in the form of the 10 questions
32	Is there a description of diverse cases or discussion of minor themes?	Yes where applicable in the text
