# Supplementary material for: How can community pharmacists be supported to manage skin conditions? A multistage stakeholder research prioritisation exercise
Source: BMJ Open. 2024 Jan 2;14(1):e071863. doi: 10.1136/bmjopen-2023-071863 (PMC10773317; doi:10.1136/bmjopen-2023-071863)
Supplement: Supplementary data [file bmjopen-2023-071863supp004.pdf]

Introduction

The responses from two open questions from the Aston Pharmacy Dermatology Survey have been analysed.

We have:

- Identified the top 20 words from the two open questions; the questions concerned are the “challenge” question and the “research priority” question.
- Created word trees from the most common words identified, then used thematic analysis of the word trees to draw up the long list of questions.

Please see below for an example of the analyses mentioned above.

Challenge word analysis

Challenge question: Please list at least one and up to two of the most challenging aspects of treating or managing patients presenting with skin conditions. For example: "How can community pharmacy best support safe use of topical corticosteroids?" or "How do we know when to refer someone with a mole?"

Number of challenge word responses=348

Top 20 words from the challenge question

Word	Count	Similar words
**refer**	44	refer, referal, referred, referring
**rash**	39	rash, rashes
**products**	26	product, products
**differential**	25	differential, differentials, differentiate, differentiating
**know**	24	know, knowing
**diagnosis**	22	diagnosis
**different**	21	difference, differences, different, differently
**steroid**	19	steroid, steroids
**need**	16	need, needed, needing, needs
**support**	16	support, supporting
**treatment**	16	treatment, treatments
**creams**	15	cream, creams
**infected**	15	infected, infection, infections
**time**	15	time, timely, times
**limited**	14	limitations, limited, limits
**difficult**	14	difficult
**emollients**	14	emollient, emollients
**identify**	14	identify, identifying
**treat**	14	treat, treated, treating
**mole**	14	mole, moles

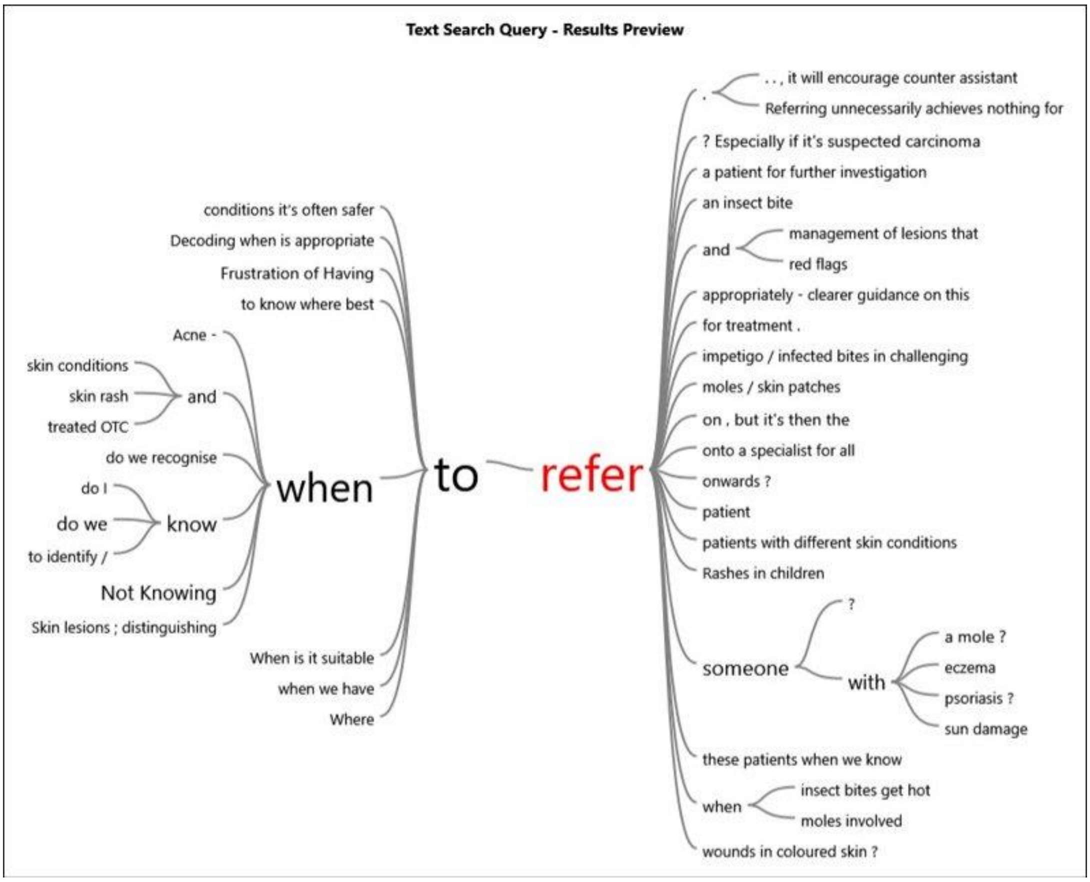
Text Search Query - Results Preview
conditions it's often safer
Decoding when is appropriate
Frustration of Having
to know where best
Acne -
skin conditions
skin rash
treated OTC
and
do we recognise
do I
do we
to identify /
know
Not Knowing
Skin lesions ; distinguishing
when
When is it suitable
when we have
Where
to
refer
. . , it will encourage counter assistant
Referring unnecessarily achieves nothing for
? Especially if it's suspected carcinoma
a patient for further investigation
an insect bite
and
management of lesions that
red flags
appropriately - clearer guidance on this
for treatment .
impetigo / infected bites in challenging
moles / skin patches
on , but it's then the
onto a specialist for all
onwards ?
patient
patients with different skin conditions
Rashes in children
someone
?
with
a mole ?
eczema
psoriasis ?
sun damage
these patients when we know
when
insect bites get hot
moles involved
wounds in coloured skin ?

Research priority analysis question

Please list at least one and up to three of your top research priorities (e.g.what do we need to know more about) in relation to the role of community pharmacy in skincare (consider both advice/counter prescribing and advice on prescription medications).

Number of research priority responses - 522

Top 20 words – research priority questions

Word	Count	Similar words
**treatment**	47	treatment, treatments
**different**	32	difference, different, differing
**products**	31	product, products
**need**	28	need, needed, needing, needs
**creams**	24	cream, creams
**rashes**	22	rash, rashes
**care**	21	care
**steroid**	21	steroid, steroidal, steroids
**pharmacists**	20	pharmacist, pharmacists
**eczema**	19	eczema
**psoriasis**	18	psoriasis
**training**	18	train, trained, training
**prescribing**	17	prescribe, prescribed, prescriber, prescribers, prescribing
**diagnosis**	16	diagnosis
**information**	16	inform, information
**effects**	15	effecting, effective, effectively, effectiveness, effects
**acne**	15	acne
**advice**	15	advice
**infections**	15	infected, infection, infections
**referral**	15	referral

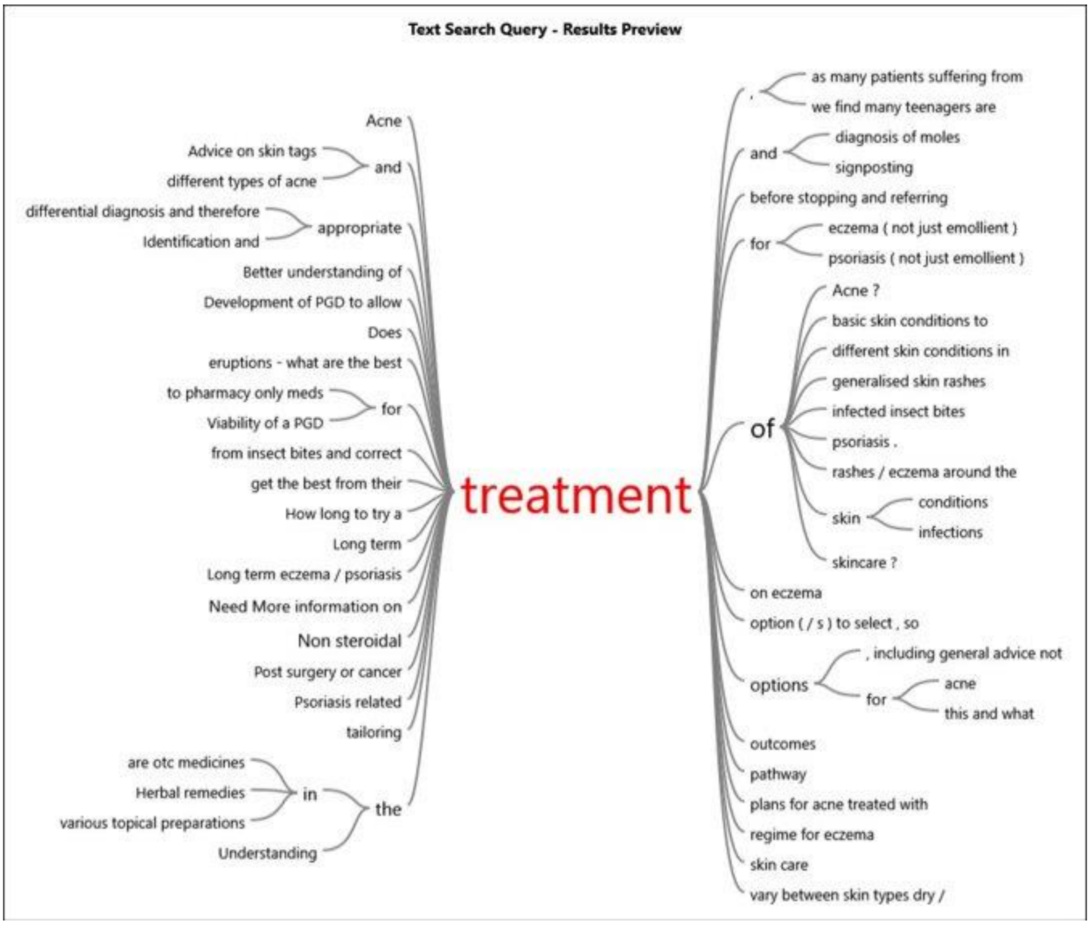
Text Search Query - Results Preview
treatment
Acne
Advice on skin tags
different types of acne
and
differential diagnosis and therefore
Identification and
appropriate
Better understanding of
Development of PGD to allow
Does
eruptions - what are the best
to pharmacy only meds
Viability of a PGD
for
from insect bites and correct
get the best from their
How long to try a
Long term
Long term eczema / psoriasis
Need More information on
Non steroidal
Post surgery or cancer
Psoriasis related
tailoring
are otc medicines
Herbal remedies
various topical preparations
in
Understanding
the
,
as many patients suffering from
we find many teenagers are
and
diagnosis of moles
signposting
before stopping and referring
for
eczema (not just emollient)
psoriasis (not just emollient)
of
Acne ?
basic skin conditions to
different skin conditions in
generalised skin rashes
infected insect bites
psoriasis .
rashes / eczema around the
skin
conditions
infections
skincare ?
on eczema
option (/ s) to select , so
options
, including general advice not
for
acne
this and what
outcomes
pathway
plans for acne treated with
regime for eczema
skin care
vary between skin types dry /
