# Supplementary material for: How can community pharmacists be supported to manage skin conditions? A multistage stakeholder research prioritisation exercise
Source: BMJ Open. 2024 Jan 2;14(1):e071863. doi: 10.1136/bmjopen-2023-071863 (PMC10773317; doi:10.1136/bmjopen-2023-071863)
Supplement: Supplementary data [file bmjopen-2023-071863supp005.pdf]

Further quotes to support research questions derived from Themes 1 and 2 – Resources to support Identifying and diagnosing skin conditions and resources to support Identifying and diagnosing skin conditions in skin of colour

Resources to help the Identification of skin conditions within community pharmacies	*"Yeah, if someone says they have dry eyes, you don't recognize patterns. You just accept you have dry eyes" Pharmacist*<br><br>*"I think a lot of dermatology things sometimes we've only seen them in pictures, so not actually in real life. And obviously everyone's different and they don't necessarily present how we've seen them in pictures so you're kind of making a judgment call whether it's close enough to the picture or whether it's something completely different that they're presenting with." Pharmacist*
Resources to help the Identification of skin conditions in skin of colour within community pharmacies	*"OK, I use Google images quite a bit for this because they're like what does it look like in a darker skin tones it and Google Google's great. But you know, like, I I don't know if this is like, you should say, oh, this is looking better. I can't say that in a different colour skin tone." Pharmacist*<br><br>*"Yeah, my worst fear is missing a red flag in like a darker skin tone like cancer. You're more likely to know what it looks like on Caucasian skin, but on dark skin I just worry that I missed the red flag." Pharmacist*

Further quotes to support the research question derived from Theme 3 - Resources to support referral

Resources to support referral of skin conditions	*"I probably wouldn't have gone to A&E had I not sort advice from them first, because I wouldn't have wanted to bother A&E. And so you almost feel like a bit better about going to and when they're really busy about what is just this little thing on your neck, because actually you can say, well, I've just been into boots and I was actually advised to come here and that makes you feel a little bit sort of that you've already gone and sought a bit of help first." Patient*<br><br>*"it would be really nice to think if the pharmacist really wanted to be useful and escalate the problem they knew where to send it to, because GPS also generally don't know that much about [skin] because there's thousands of different skin conditions" Patient*<br><br>*"My fear is we go give out antibiotics for that, or impetigo like give the Fucidin cream out for that and we actually delay treatment for something that maybe is a bit more serious." Pharmacist*<br><br>*"It is sometimes difficult even after all this time it is difficult to determine exactly or to have confidence in what you think a particular rash might be and how infectious it is or is not" Pharmacist*<br><br>*"I think the with children with any sort of, any really well, anytime a child comes to you in a pharmacy, I think you just. You feel. More pressure to make the right decision because. You refer just in case it is something more serious because you've got that doubt in the back of your mind." Pharmacist*<br><br>*"Yes, I struggled to refer things to the GP and just cos our GP is getting massive very quickly. We've got a lot of new builds in our area and so they are quite snowed under and generally I think any appointment time is about four weeks and so it is a struggle to refer and normally we just say if you mentioned that you've been to us because we are opposite, we are, we share a gangway so they are quite nice about it, but it's a struggle to say this is serious enough to be seen tomorrow." Pharmacist*<br><br>*"The idea of suggesting to GP that they sort of take on more of a training role or sort of a feedback system with a community pharmacist might not land very well, but in an ideal world I think it's a great a great idea. I would. I would love to have. So[me] two way dialogue, you know, even if if they if they send pictures through, like our district nurses send pictures of patients all the time of the of a wound on their leg and say do you think this needs antibiotics?" GP*

Further quotes to support the research question derived from Theme 4- Working with other healthcare professionals

**Working with other healthcare professionals**	
Initial management of skin conditions	*"Because what I usually tell patients is in this climate if you're not going to get into the GPS for a few weeks, you may as well try one week of hydrocortisone and this emollient and then you have that information to tell the prescriber. If it's better, brilliant if it's worse, at least you won't be sent away with hydrocortisone and an emollient, and you'll already be one step ahead, but I just don't know whether that's the most useful action, so I think just more of a conversation." Pharmacist*<br><br>*"I think going from Community pharmacy to now working alongside GPs when I do have a a concern about a skin condition or some sort of query, you find that a lot of them actually just use trial and error with different prescription only medications and creams. So they'll just say try this for a week If it doesn't work then come back and a lot of it, even they're not 100% sure." Pharmacist*
Roles in management of long-term skin conditions	*"I I think I think if people have got, […] already got a diagnosis. I think that [upskilling pharmacists to provide counselling] would be really helpful because you know follow up appointments are getting more and more difficult. I think everything's changed so much in the last 20 years because we've now got biologics. So in in secondary care then we're mainly focusing on patients who have had the systemic medications at the topicals, the systemics." Specialist dermatology nurse*<br><br>*"So in all the years that I've been having these meds I've never been offered any kind of advice except for once. When the pharmacist said don't forget to keep these in the fridge because they don't like lots of hot hot temperature." Patient*

Further quotes to support the research questions derived from Theme 5 - Resources to support the management of skin conditions and improving access to medications

Management of skin conditions	*I just always find it difficult with eczema, just the amount of emollients, and it's what emollients to recommend and what circumstance. Pharmacist*
Providing a wider range of treatments	*"I find it difficult to treat over the counter because I feel quite limited in what we're what we're licensed to sell and for what and for use for, like on on the areas of the body" Pharmacy dispenser*<br><br>*"I can't tell you how many referrals I've made for impetigo because there's just nothing to offer" Pharmacist*
Access to topical corticosteroids	*"And as someone mentioned, you have to be quite careful of what you say to them in order to get it [TCS]. " Patients*<br><br>*"I might treat something off license and and pharmacists are very much by the book. But in dermatology, we can't always do things by the book and we have to use things off licence because sometimes there's just nowhere else to go. I mean, you know, I for years, I I had a paediatric eczema clinic. And I just felt so sorry for those parents who, even with the GP, found it difficult to to get the medication that they needed if their children's skin was flaring and they had quite severe flares and they had to wait to come and see us and we only had it clinic once once a week." Specialist dermatology nurse*<br><br>*"[the patient said] I've forgotten my cream and they wanted to buy hydrocortisone cream and they said I've got eczema on my face, and you simply can't sell it. And and and you get into it. So so in many respects you you've got kind of silly restrictions like that which actually impede your ability to practice properly because you think because you effectively look a bit stupid because, well, the doctor gives it to me and you're saying you can't sell it to me and you think what is all that about?" Pharmacist*<br><br>*"I'm not concerned about pharmacists supplying the items inappropriately, but it's the whole over the counter consultation because this my staff know that the face is a no go zone for hydrocortisone. So if somebody was requesting it for the face, that's a hard kind of stop for them to come and grab me to have a conversation with the patient about this. Whereas if that licensing was removed, you may run* the *risk of just missing these patients throughout the whole pharmacy team." Pharmacist*

Further quotes to support Research Question from Theme 6 - Current involvement of pharmacists and the known benefits of the involvement of community pharmacists in the identification and management of skin conditions

Assessment of rising demand	*"And I see such a broad range [..] I just see a bit of everything all the time" Pharmacist*<br><br>*"but I do get a lot of people coming in children with children. All sorts of skin conditions. As you said ringworm and eczema and slap cheek." Pharmacist*<br><br>*"I think it has [consultations for skin disease] increased, but I was still seeing quite a few skin conditions, so people coming in asking before COVID as well asking what it might be and anything [I] could recommend." Pharmacist*<br><br>*"I think it will, I think it will stay [the increased demand], UM I, I think. [It's] the ease of access you know? And because people are not getting appointments with GP" Pharmacist*
Variation in service provisions	*"I think the main ailments aspect needs to be stretched. To cover all areas of the country to make it easier for pharmacies in areas that don't have it. Because some of the things that we can't assist with are ridiculous." Pharmacy dispenser*<br><br>*"[the geographical area] has some PGDs that allow for supply, for example, hydrocortisone on the face under PGD" Pharmacist*<br><br>*"You know, and if I've got the choice of trying to get an appointment to see a GP. And then they'll give me a prescription, and then because I've got a prepayment, I don't have to pay for it or going to a chemist and then charging me £9 for a bottle of Double Base Ointment. The logic says you go to the GP first. You've just saved yourself. You know, a tenner" Patient*<br><br>*"I'm not a massive fan of the CPCS thing yet, cause UM our area doesn't do much minor ailments and they're expecting things for free. And I'm, they're like, no, I can't. I can't give you that you're gonna have to. You're gonna have to pay." Pharmacist*<br><br>*" How well do you trust somebody's clinical judgment against their commercial need?" Patient*

Further quotes to support Research Question 9. and 10. Competency of community pharmacists in managing skin conditions and awareness of the public of the skills of community pharmacists with regards to the identification and management of skin conditions.

Pharmacist competency in the identification and treatment of skin conditions	*"I would guess a certificate on the wall […] go to my optician and he's got all his different specialities on on the wall. So something just with simple as that and I guess they would have to be trained by a consultant dermatologist or at least somebody with many years of experience at spotting all kinds of different conditions." Patient*<br><br>*"I think one I don't know what community pharmacists know, and I think that's part of the problem. We work very closely with community pharmacists in some ways, but we're very, very separate in others, so we phone them up about medication queries and they phone us all the time. But actually knowing what each other's skills are is is quite difficult." GP*<br><br>*"But what I do see is what they've attempted to treat and then comes to us anyway, so I see quite a lot of misdiagnoses from community pharmacies, so a lot of insect bites that look very red and inflamed are sent to us because they think it's infected […] we see lots of sort of cold sore sent to us as query impetigo needing antibiotics, lots of eczema that's just red and inflamed might be infected and gets sent to us and it's not, it just needs eczema treatment so I think. I I see some fairly basic areas of of lacking in knowledge […] but what I don't see are the ones that they have successfully treated and and and sent away so I don't know what sort of ratio we're talking here." GP*
Raising awareness of pharmacy skin services	*"I've had various sort of short term skin conditions and pharmacies have always been my sort of first port of call. It [has] always been good experiences" Patient*<br><br>*"I never knew the pharmacy that they could advise you on skin you know problems, I never knew that. Because I thought they dealt dealt with drugs only and they all seemed very busy. So I'd like to know how the pharmacist can help with skin conditions as well?" Patient*<br><br>*"This minor ailment scheme and for that it needs the patient consent, so we need them to reply to the text message or we need to speak to them on the phone to get their consent and then make the referral, which is a very quick process, but it's all quite clunky and it doesn't always have the patient buy in because they, sometimes incorrectly, value the GP's skills more than the community pharmacists skills, so there needs to be a degree of sort of selling of this and patient education" GP*
