# Supplementary material for: How can community pharmacists be supported to manage skin conditions? A multistage stakeholder research prioritisation exercise
Source: BMJ Open. 2024 Jan 2;14(1):e071863. doi: 10.1136/bmjopen-2023-071863 (PMC10773317; doi:10.1136/bmjopen-2023-071863)
Supplement: Supplementary data [file bmjopen-2023-071863supp006.pdf]

N.B. In the notes below, where we refer to community pharmacists, we include any appropriately qualified member of the community pharmacy team.

Question	Explanatory notes
1. What are the known benefits of community pharmacy involvement in the identification and management of skin conditions?	• This could be about establishing what is already known about community pharmacy involvement in the management of skin conditions.<br>• This could be important research which underpins the development of new services or resources.<br>• In our focus groups, participants described several different activities where community pharmacists have helped people with skin conditions. Some examples are these activities are; diagnosing, supporting self-management, recommending the use of products, and participating in dedicated medicines/illness schemes. Knowing which activities have evidence of being beneficial could support future provision.
2. In what ways are community pharmacists currently involved in the identification and management of skin conditions?	• This could be about developing a more detailed understanding about what currently happens in community pharmacy.<br>• It could include assessing levels of demand, mapping different types of activity, and identifying novel ways of working.<br>• In our focus groups it was apparent that services are developing differently across the country and that people were not aware of all that is currently happening in different areas.
3. How competent are community pharmacists in the identification and management of skin conditions?	• This could involve assessing the knowledge and skills of community pharmacists and other members of the pharmacy team.<br>• In our focus groups pharmacists described different levels of confidence and competence in addressing skin conditions. Members of the public were uncertain about how qualified community pharmacists are to manage skin conditions.
4. Would dedicated resources improve the **identification of skin conditions** by community pharmacists?	• This could involve developing and/or testing resources which support community pharmacists in identifying skin conditions. Resources might be online resources, training programmes etc.<br>• In our focus groups pharmacists described an absence of resources that they are in confident in using to identify skin conditions, they said that high quality resources could be useful in identifying skin conditions.
5. Would dedicated resources improve the **identification of skin conditions** in **skin of colour** by community pharmacists?	• This could involve developing and/or testing resources which support community pharmacists in identifying skin conditions in skin of colour.<br>• In our focus groups pharmacists described that identifying skin conditions in skin of colour is an additional challenge due to the different ways that skin conditions can look, and also because most current training/resources present images of white skin.
6. Would dedicated resources support community pharmacists in the **management** of skin conditions?	• This could involve developing and/or testing resources which support community pharmacists in managing skin conditions.<br>• In our focus groups pharmacists described being unsure about when they might take an active role in skin condition management. They also wanted

Question	Explanatory notes
	evidence-based information about what support and products they can provide.<br><br>• This might focus upon specific conditions (eczema, psoriasis, insect bites, etc.) or could be for more generic conditions (a rash, dry skin, etc.).
7. Would dedicated resources support community pharmacists to **effectively refer skin conditions** that require urgent or more specialist attention?	• This could involve developing and/or testing resources which support community pharmacists in referring skin conditions to other sources of healthcare (e.g. emergency care, general practice, etc.).<br><br>• It might include developing referral pathways and new services for specific serious skin conditions.<br><br>• In our focus groups pharmacists described feeling uncertain about when to take this sort of action; members of the public recognised that sometimes they wanted a pharmacist to 'tell them to go to the doctor'.
8. Could a wider range of products and treatments for skin conditions be made available via community pharmacy?	• This might include considering treatments which are restricted due to legal reasons (e.g. can only be supplied against a prescription) or economic reasons (e.g. where price might inhibit purchase).<br><br>• In our focus groups both pharmacists and members of the public described frustration that certain products are restricted and cannot be provided by a pharmacist.
9. What could be done to raise awareness of the skills that community pharmacists have with regards to the identification and management of skin conditions?	• This could involve local or national campaigns to raise awareness about community pharmacy as a healthcare resource. It might focus upon how community pharmacy services are understood by members of the public.<br><br>• In our focus groups some members of the public were not aware that community pharmacists are able to provide healthcare advice about skin conditions. Some members of the public were concerned that commercial concerns might be a priority for pharmacists, i.e. selling products might be more important than providing appropriate advice.
10. How can community pharmacists work most effectively with other healthcare professionals in the identification and management of skin disease?	• This might involve new integrated services for the management of skin conditions that include community pharmacists and other healthcare providers (e.g. GPs, nurses, dermatologists). It could include the use of pharmacist independent prescribers or pharmacists with additional training in dermatology.<br><br>• In our focus groups pharmacists reported working in isolation with little or no feedback about the advice and services that they offer. They also indicated that it is sometimes difficult to contact or engage with other healthcare providers about skin conditions.
