# Supplementary material for: How can community pharmacists be supported to manage skin conditions? A multistage stakeholder research prioritisation exercise
Source: BMJ Open. 2024 Jan 2;14(1):e071863. doi: 10.1136/bmjopen-2023-071863 (PMC10773317; doi:10.1136/bmjopen-2023-071863)
Supplement: Author's manuscript [file bmjopen-2023-071863.draft_revisions.pdf]

BMJ Open

BMJ Open is committed to open peer review. As part of this commitment we make the peer review history of every article we publish publicly available.

When an article is published we post the peer reviewers' comments and the authors' responses online. We also post the versions of the paper that were used during peer review. These are the versions that the peer review comments apply to.

The versions of the paper that follow are the versions that were submitted during the peer review process. They are not the versions of record or the final published versions. They should not be cited or distributed as the published version of this manuscript.

BMJ Open is an open access journal and the full, final, typeset and author-corrected version of record of the manuscript is available on our site with no access controls, subscription charges or pay-per-view fees (http://bmjopen.bmj.com).

If you have any questions on BMJ Open's open peer review process please email info.bmjopen@bmj.com

BMJ Open

Supporting community pharmacists in managing skin conditions; a research priority setting exercise.

Journal:	*BMJ Open*
Manuscript ID	bmjopen-2023-071863
Article Type:	Original research
Date Submitted by the Author:	26-Jan-2023

Complete List of Authors:	Harvey, Jane; University of Nottingham Centre for Evidence Based Dermatology<br>Shariff, Zakia; Aston University Aston Pharmacy School<br>Anderson, Claire ; University of Nottingham School of Pharmacy<br>Boyd, Matthew; University of Nottingham School of Pharmacy, School of Pharmacy<br>Ridd, Matthew; University of Bristol Faculty of Health Sciences, Population Health Sciences<br>Santer, Miriam; University of Southampton, Primary Care Research Centre,<br>Thomas, Kim; University of Nottingham Centre for Evidence Based Dermatology, Dermatology<br>Maidment, Ian; Aston University School of Life and Health Sciences, Aston Pharmacy School<br>Leighton, Paul; University of Nottingham Centre for Evidence Based Dermatology

Keywords:	DERMATOLOGY, Patient Participation, QUALITATIVE RESEARCH, PRIMARY CARE

SCHOLARONE™
Manuscripts

I, the Submitting Author has the right to grant and does grant on behalf of all authors of the Work (as defined in the below author licence), an exclusive licence and/or a non-exclusive licence for contributions from authors who are: i) UK Crown employees; ii) where BMJ has agreed a CC-BY licence shall apply, and/or iii) in accordance with the terms applicable for US Federal Government officers or employees acting as part of their official duties; on a worldwide, perpetual, irrevocable, royalty-free basis to BMJ Publishing Group Ltd ("BMJ") its licensees and where the relevant Journal is co-owned by BMJ to the co-owners of the Journal, to publish the Work in this journal and any other BMJ products and to exploit all rights, as set out in our licence.

The Submitting Author accepts and understands that any supply made under these terms is made by BMJ to the Submitting Author unless you are acting as an employee on behalf of your employer or a postgraduate student of an affiliated institution which is paying any applicable article publishing charge ("APC") for Open Access articles. Where the Submitting Author wishes to make the Work available on an Open Access basis (and intends to pay the relevant APC), the terms of reuse of such Open Access shall be governed by a Creative Commons licence – details of these licences and which Creative Commons licence will apply to this Work are set out in our licence referred to above.

Other than as permitted in any relevant BMJ Author's Self Archiving Policies, I confirm this Work has not been accepted for publication elsewhere, is not being considered for publication elsewhere and does not duplicate material already published. I confirm all authors consent to publication of this Work and authorise the granting of this licence.

For peer review only

Title: Supporting community pharmacists in managing skin conditions; a research priority setting exercise.

Proposed journal for submission: BMJ open

Authors: J Harvey[1#], Z Shariff[2], C Anderson[3], MJ Boyd[3], MJ Ridd[4] M Santer[5], KS Thomas[1], I Maidment[2] and P Leighton[1]

Affiliations

[1]Centre of Evidence Based Dermatology, School of Medicine, University of Nottingham, Nottingham, UK
[2] Aston Pharmacy School, Aston University, Birmingham, UK.
[3] Division of Pharmacy Practice and Policy, School of Pharmacy, University of Nottingham, Nottingham, UK
[4]Population Health Sciences, University of Bristol, Bristol, UK
[5] Primary Care Research Centre, University of Southampton, Southampton, UK
# Corresponding author jane.harvey1@nottingham.ac.uk Centre of Evidence Based Dermatology, Applied Health Research Building, School of Medicine, University of Nottingham, NG7 2RD

ORCiD

J Harvey 0000-0003-1402-6116
Z Shariff 0000-0003-4521-0864
C Anderson 0000-0002-5406-2296
MJ Boyd 0000-0003-2997-5090
MJ Ridd 0000-0002-7954-8823
M Santer 0000-0001-7264-5260
KS Thomas 0000-0001-7785-7465
I Maidment 0000-0003-4152-9704
P Leighton 0000-0001-5208-0274

Word count

Abstract 297

Body of text 4131

Acknowledgements

Thank you also to all the members of pharmacy staff, other healthcare professionals and members of the public that took part in interviews or workshops. Particular thanks goes to Amanda Roberts and our other patient representative for their help with this work.

For peer review only

Exclusive Licence

I, the Submitting Author has the right to grant and does grant on behalf of all authors of the Work (as defined in the below author licence), an exclusive licence and/or a non-exclusive licence for contributions from authors who are: i) UK Crown employees; ii) where BMJ has agreed a CC-BY licence shall apply, and/or iii) in accordance with the terms applicable for US Federal Government officers or employees acting as part of their official duties; on a worldwide, perpetual, irrevocable, royalty-free basis to BMJ Publishing Group Ltd ("BMJ") its licensees and where the relevant Journal is co-owned by BMJ to the co-owners of the Journal, to publish the Work in BMJ Open and any other BMJ products and to exploit all rights, as set out in our licence.

The Submitting Author accepts and understands that any supply made under these terms is made by BMJ to the Submitting Author unless you are acting as an employee on behalf of your employer or a postgraduate student of an affiliated institution which is paying any applicable article publishing charge ("APC") for Open Access articles. Where the Submitting Author wishes to make the Work available on an Open Access basis (and intends to pay the relevant APC), the terms of reuse of such Open Access shall be governed by a Creative Commons licence – details of these licences and which Creative Commons licence will apply to this Work are set out in our licence referred to above.

For peer review only

ABSTRACT

Objective

To establish research priorities which will support the development and delivery of community pharmacy initiatives for the management of skin conditions.

Design

An iterative, multi-stage stakeholder consultation consisting of online survey, participant workshops, and prioritisation meeting.

Setting

All data collection took place online with participants completing a survey (delivered via the JISC Online Survey platform, between July 2021 and January 2022) and participating in online workshops and meetings (hosted on Microsoft Teams between April and July 2022).

Participants

174 community pharmacists and pharmacy staff completed the online survey.

53 participants participated in the exploratory workshops (19 community pharmacists, 4 non pharmacist members of pharmacy staff and 30 members of the public). 4 healthcare professionals who were unable to attend a workshop participated in a one-to-one interview.

29 participants from the workshops took part in the prioritisation meeting (5 pharmacists/pharmacy staff, 1 healthcare professional, and 23 members of the public).

Results

Five broad areas of potential research need were identified in the online survey:(1) identifying and diagnosing skin conditions; (2) skin conditions in skin of colour; (3) when to refer skin conditions; (4) disease specific concerns; and (5) product specific concerns.

These were explored and refined in the workshops to establish ten potential areas for research which will support pharmacists in managing skin conditions. These were ranked in the prioritisation meeting. Amongst those prioritised were topics which consider how pharmacists work with other healthcare professionals to identify and manage skin conditions.

Conclusions

Survey responses and stakeholder workshops all recognised the potential for community pharmacists to play an active role in the management of common skin conditions. Future research may support this in the generation of resources for pharmacists, in encouraging public take-up of pharmacy services, and in evaluating the most effective provision for dealing with skin conditions.

For peer review only

STRENGTHS AND LIMITATIONS

- Novel exploration of the research needs associated with the care of skin conditions within community pharmacy.
- An iterative, multi-stage consultation ensured detailed insight about the topic.
- The involvement of pharmacists, pharmacy staff, healthcare professionals and members of the public ensured that all pertinent voices were heard.
- Participants were self-selecting and may have had a particular interest/perspective upon skin conditions.
- Greater participation from pharmacists in the prioritisation workshops may have been beneficial.

For peer review only

INTRODUCTION

Community pharmacy is recognised as an accessible source of healthcare advice [1-3] and the COVID pandemic has cemented it more clearly in the primary care landscape for members of the public [4]. Moreover, recent initiatives, such as the Community Pharmacy Consultation Service, seek to use pharmacy more effectively by diverting the management of some minor ailments to community pharmacy settings [5].

Skin conditions are amongst the most common diseases encountered by healthcare professionals[6,7]. Each year approximately 54% of the population will experience some form of skin disease[6], at any one time up to one-third of all people will have a skin condition that warrants medical attention[6,8,9]. Skin complaints have been identified as conditions that could be potentially managed within community pharmacy [10,11] and community pharmacists recognise skin conditions as a significant part of their workload [1,12,13]. Pharmacists regularly give advice on the management of common conditions such as eczema, dermatitis, generalised rashes, allergies and acne [12] and just over one-third (38%) of all symptomatic advice requests in community pharmacy relate to skin conditions[14]. Almost 20% of pharmacy sales are for skin products[2].

As community pharmacy continues its trajectory towards expanded and extended provision,[3] support for pharmacists in dealing with queries about skin conditions may be warranted. Skin conditions can be difficult to identify [15] and prior training for pharmacists in dermatological conditions is limited [6,8,16,17]. How best to support pharmacists is, however, unclear.

The aim of this work is to establish stakeholder consensus upon those research priorities which might best support community pharmacists in their involvement in the care of patients with skin conditions.

METHODS

This was a multi-stage, iterative stakeholder consultation informed by James Lind Priority Setting Partnership method [18] consisting of: (1) an online survey, (2) exploratory workshops, and (3) a prioritisation workshop.

Participants

Stage 1 – online survey

An online survey using the JISC Online Survey platform (https://www.jisc.ac.uk/online-surveys#) was targeted to community pharmacists and other community pharmacy staff. Social media (Twitter® and Facebook) and personal and professional networks (e.g., Pharmaceutical Services Negotiating Committee (PSNC) newsletter) were used to promote the survey. A specific analysis of the survey data has been submitted for publication elsewhere.

Stage 2 – exploratory workshops

Community pharmacists, pharmacy staff, other healthcare professionals, and members of the public were recruited to a series of workshops to explore potential research topics which might support the management of skin conditions in community pharmacy.

Social media (Twitter® and Facebook) and personal and professional networks (e.g., Community Pharmacy Dermatology Network, primary care networks) were again used to recruit pharmacists, pharmacy staff as well as healthcare professionals. Additional professional networks (the Primary Care Dermatology Society, the Society for Academic

For peer review only

Primary Care Skin Special Interest Group and the UK Dermatology Clinical Trials Network) were used to recruit healthcare professionals.

Members of the public were recruited via social media and existing public and patient research networks (e.g. the CEBD patient panel and "People in Research" (https://www.peopleinresearch.org/)).

Where any individual was not able to attend a scheduled workshop they were offered the opportunity to take part in a brief one-to-one interview.

Stage 3 – prioritisation workshops

Exploratory workshop participants were subsequently invited to take part in the prioritisation workshop.

Data collection and analysis

Stage 1 – online survey

Survey responses were collected over a period of six months between 20th July 2021 and 20th January 2022.

Content analysis of free text responses was used to identify commonly used words and phrases. Selected words or phrases (frequently used or substantively important) were reviewed thematically[19].

Stage 2 - exploratory workshops and interviews

Workshops and interviews were undertaken online using Microsoft Teams and took place between April and July 2022.

Workshops and interviews were structured according to the thematic analysis of stage 1 data, with key themes explored further through group discussion. All discussions were digitally recorded with permission. Digital recordings were automatically transcribed verbatim and anonymised.

Framework analysis[20] was used to map workshop and interview data to broad uncertainties identified in stage 1. Synthesis of data and interpretation of synthesised data led to the creation of narrower research topics.

Stage 3 - prioritisation workshop

Following the conventions of the Nominal Group Technique[21] research topics were shared with participants prior to the prioritisation workshop. During the workshop group discussion and item scoring were used iteratively to reject and rank topics. Simple, descriptive statistics were used to rank and establish consensus upon priority research topics (i.e., the percentage of respondents selecting a topic for inclusion / priority). For further information regarding methods please see Supplementary File 1.

Patient Involvement

Before the study started, we met with two PPI collaborators to provide an overview of the study and the study methodology. One PPI member collaborated with us to develop the participant information leaflet. They also attended the steering group meeting where we developed the final list of research questions. The other PPI collaborator assisted with recruitment of patients via social media and recommended other areas where we could recruit participants e.g. the "people in research" website. They also attended one of our patient focus groups.

For peer review only

RESULTS

The numbers and characteristics of participants that took place at each stage of the process are shown in Table 1.

Table 1 Numbers of participants included at each stage of the priority setting exercise

	**Numbers of participants**		
	Stage One	Stage Two	Stage Three
**Type of participant**			
Patients	N/A	30	23
Pharmacists	111	19	3
Other members of pharmacy staff	63	4	2
Specialist dermatology nurse	N/A	1	1
GPs	N/A	3	0

Stage 1 – online survey

The survey was completed by 174 participants. Word counts and an example of the word trees are available in Supplementary File 2.

The most reported five words in response to the "challenge" questions within the survey were "refer, rash, products, differential and know" whilst in the "research priorities" question, the most commonly reported words were "treatment, different, products, need and creams". These and the remaining top 20 words from each question encompass a broad range of research challenges, which were reflected in the five key areas of the analytic framework detailed in Table 2.

Table 2 Original analytic framework developed from survey responses

Identifying and diagnosing skin conditions
Skin of colour
Knowing when to refer skin conditions to a GP
Disease specific concerns
Product specific concerns

Stage 2 – exploratory workshops and one to one interviews

Nine workshops were held, and four additional interviews were undertaken to facilitate those unable to attend a scheduled workshop. Workshops lasted between one and two hours, interviews were typically around thirty minutes.

Four workshops consisted of pharmacists (19 participants), one included only pharmacy staff (4 participants), and four workshops contained only members of the public (30 participants). Interviews were undertaken with three GPs and one dermatology nurse specialist (Table 1).

Theme 1 – identifying and diagnosing skin conditions.

The challenge of identifying and diagnosing skin conditions was a common focus for those workshops involving pharmacists and pharmacy staff. It was frequently described as a source of stress:

For peer review only

"Skins a nightmare"
(Workshop 2, Pharmacist 1)

"One of the worst things I can hear in a pharmacy is when a patient says, "can I speak to the pharmacist? Can they tell me what this rash is my child has got?""
(Workshop 2, Pharmacist 2)

Difficulties identifying *reliable* resources which could support practice were recognised. Google images, the National Health Service (NHS) website, Clinical Knowledge Summaries website (CKS) or National Institute for Health and Care Excellent (NICE) guidelines were all discussed, but using *standard* photographs was not always found to be helpful. That some resources (e.g. CKS and NICE) do not contain images further impacts upon their utility.

Pharmacists explained that this is particularly an issue with skin conditions as members of the public commonly show them affected skin, rather than verbally describing symptoms as they do with other conditions (Table 3).

For peer review only

244

245 *Table 3 Further quotes to support research questions derived from Themes 1 and 2 – Resources to support Identifying and diagnosing skin conditions*
246 *and resources to support Identifying and diagnosing skin conditions in skin of colour*

Resources to help the Identification of skin conditions within community pharmacies	*"Yeah, if someone says they have dry eyes, you don't recognize patterns. You just accept you have dry eyes" Pharmacist*<br><br>*"I think a lot of dermatology things sometimes we've only seen them in pictures, so not actually in real life. And obviously everyone's different and they don't necessarily present how we've seen them in pictures so you're kind of making a judgment call whether it's close enough to the picture or whether it's something completely different that they're presenting with." Pharmacist*
Resources to help the Identification of skin conditions in skin of colour within community pharmacies	*"OK, I use Google images quite a bit for this because they're like what does it look like in a darker skin tones it and Google Google's great. But you know, like, I I don't know if this is like, you should say, oh, this is looking better. I can't say that in a different colour skin tone." Pharmacist*<br><br>*"Yeah, my worst fear is missing a red flag in like a darker skin tone like cancer. You're more likely to know what it looks like on Caucasian skin, but on dark skin I just worry that I missed the red flag." Pharmacist*

247

For peer review only

The development of pharmacy specific resources (e.g. online toolkits, in person training etc.) was recognised as a potentially important area for future research and action.

> Possible Research Question - Would dedicated resources improve the identification of skin conditions in community pharmacy?

Theme 2 - Identifying and diagnosing skin conditions in skin of colour

Discussion of skin of colour proceeded almost as an extension of theme 1. With a few exceptions, most participants described identifying skin conditions in skin of colour as more difficult:

> *"I know fungal infection definitely look[s] different on like very dark skin, but I don't know whether my diagnosis would be right, so I'm just always doubting myself. Yeah, so one other thing is, um, discoid eczema [and] ringworm they look very similar on like dark skin or fair skin. So that comes up all the time. I get asked whether it's eczema ringworm all the time. And I don't know the difference."*
> *(Workshop 1, Pharmacist 3)*

Knowing when a condition was getting worse was also considered more challenging (Table 3).

Again, an absence of *reliable, high-quality, evidence-based* resources was considered a barrier to effectively responding to queries and questions about skin conditions in skin of colour.

> Possible Research Question - Would dedicated resources improve the identification of skin conditions in skin of colour in community pharmacy?

Theme 3 - Knowing when to refer skin conditions

Members of the public described using community pharmacy as a form of triage, seeking advice about whether a condition was "*serious enough*" to consult other healthcare professionals (Table 4).

For peer review only

Table 4 Further quotes to support the research question derived from Theme 3 - Resources to support referral

Resources to support referral of skin conditions	*"I probably wouldn't have gone to A&E had I not sort advice from them first, because I wouldn't have wanted to bother A&E. And so you almost feel like a bit better about going to and when they're really busy about what is just this little thing on your neck, because actually you can say, well, I've just been into boots and I was actually advised to come here and that makes you feel a little bit sort of that you've already gone and sought a bit of help first." Patient*<br><br>*"it would be really nice to think if the pharmacist really wanted to be useful and escalate the problem they knew where to send it to, because GPS also generally don't know that much about [skin] because there's thousands of different skin conditions" Patient*<br><br>*"My fear is we go give out antibiotics for that, or impetigo like give the Fucidin cream out for that and we actually delay treatment for something that maybe is a bit more serious." Pharmacist*<br><br>*"It is sometimes difficult even after all this time it is difficult to determine exactly or to have confidence in what you think a particular rash might be and how infectious it is or is not" Pharmacist*<br><br>*"I think the with children with any sort of, any really well, anytime a child comes to you in a pharmacy, I think you just. You feel. More pressure to make the right decision because. You refer just in case it is something more serious because you've got that doubt in the back of your mind." Pharmacist*<br><br>*"Yes, I struggled to refer things to the GP and just cos our GP is getting massive very quickly. We've got a lot of new builds in our area and so they are quite snowed under and generally I think any appointment time is about four weeks and so it is a struggle to refer and normally we just say if you mentioned that you've been to us because we are opposite, we are, we share a gangway so they are quite nice about it, but it's a struggle to say this is serious enough to be seen tomorrow." Pharmacist*<br><br>*"The idea of suggesting to GP that they sort of take on more of a training role or sort of a feedback system with a community pharmacist might not land very well, but in an ideal world I think it's a great a great idea. I would. I would love to have. So[me] two way dialogue, you know, even if if they if they send pictures through, like our district nurses send pictures of patients all the time of the of a wound on their leg and say do you think this needs antibiotics?" GP*

For peer review only

For some a pharmacist’s advice had been an important factor in being confident enough to seek (*demand*) a doctor’s appointment.

This was a role that pharmacists recognised but were not always comfortable with; they had specific concerns about *delaying diagnosis* of serious conditions, *missing infectious diseases* or a fear of *making a condition worse* by giving the wrong advice (Table 4):

> *“Some condition can wait for next day or next week, but some condition need to be managed quite soon. Like same day referral. So I think my challenge was whether to refer […] because weekend 111 is so busy they take hours for them to the doctor they call them back. So sometimes they like go to walk in centre or wait. So I think it's either they can wait till next day or next few days or. With that same day, it's my challenge.”*
> *(Workshop 1, Pharmacist 3)*

These concerns were considered more critical if advice was being sought about a child (Table 4).

Pharmacists also identified that it was not always easy to contact other health professionals and that it is difficult to know when and how to refer patients (Table 4). The potential for better connected services in the management of skin conditions was also recognised in one of our interviews (with a GP), although it was also recognised that resources might be a barrier to this (Table 4).

> Possible Research Question - Would dedicated resources support community pharmacists to effectively refer skin conditions that require urgent or more specialist attention?

Theme 4 - Disease specific concerns

Workshop discussion did not confirm such a strong focus upon specific skin conditions as the survey data, but rather pointed some to general challenges of managing skin conditions in community pharmacy. An absence of feedback, and of knowing the outcome of advice, was commonly described.

> *“So even though I've kind of recommended this steroid, or I've recommended this emollient, I don't know whether it worked or not because they just don't come back. Even if it's a regular customer.”*
> *(Workshop 2, Pharmacist 4)*

This makes it harder for a pharmacist to feel fully confident in the advice that they are providing. Similarly, pharmacists rarely gained feedback when referring an individual to a GP, although subsequently seeing the GP’s prescriptions might offer some informal insight.

The potential for pharmacists to be more involved in managing skin disease was commonly recognised in both the pharmacist workshops as well as healthcare professional interviews (Table 5).

For peer review only

Table 5 Further quotes to support the research question derived from Theme 4- Working with other healthcare professionals

**Working with other healthcare professionals**	
Initial management of skin conditions	*"Because what I usually tell patients is in this climate if you're not going to get into the GPS for a few weeks, you may as well try one week of hydrocortisone and this emollient and then you have that information to tell the prescriber. If it's better, brilliant if it's worse, at least you won't be sent away with hydrocortisone and an emollient, and you'll already be one step ahead, but I just don't know whether that's the most useful action, so I think just more of a conversation."* Pharmacist<br><br>*"I think going from Community pharmacy to now working alongside GPs when I do have a a concern about a skin condition or some sort of query, you find that a lot of them actually just use trial and error with different prescription only medications and creams. So they'll just say try this for a week If it doesn't work then come back and a lot of it, even they're not 100% sure."* Pharmacist
Roles in management of long-term skin conditions	*"I I think I think if people have got, […] already got a diagnosis. I think that [upskilling pharmacists to provide counselling] would be really helpful because you know follow up appointments are getting more and more difficult. I think everything's changed so much in the last 20 years because we've now got biologics. So in in secondary care then we're mainly focusing on patients who have had the systemic medications at the topicals, the systemics."* Specialist dermatology nurse<br><br>*"So in all the years that I've been having these meds I've never been offered any kind of advice except for once. When the pharmacist said don't forget to keep these in the fridge because they don't like lots of hot hot temperature."* Patient

For peer review only

A few suggested that this might be in diagnosing and suggesting initial treatments (Table 5), others focused upon counselling on long-term medication use:

> *"Perhaps a bigger and perhaps more important role for pharmacists is actually in supporting patients with long term chronic skin conditions. Because there are loads of people out there there with eczema, acne, psoriasis and so on who don't really get the best out of their treatment and end up going into secondary care because they are very poorly managed within primary care. Because the GPs don't know very much about it and I think this is a golden opportunity for community pharmacists to get more involved is actually in supporting those patients who have chronic skin conditions because this is where the majority of the the problem for the for those patients lies."*
> *(Workshop 1, Pharmacist 5)*

The potential to integrate community pharmacy with other healthcare was considered as offering benefits for the management of skin disease management.

> Possible research question - How can community pharmacists work most effectively with other healthcare professionals in the identification and management of skin disease?

Theme 5 - Product specific concerns
During the workshops pharmacists communicated that they were in the main confident about their knowledge of products used to manage skin conditions. Members of the public reinforced the importance of this by communicating that they expected pharmacists to understand the products that they were providing (either over the counter or against a prescription):

> *"It seems to me that a pharmacist should be an expert on the products. And if they're not already an expert on the products then one questions what they're doing as a pharmacist. Sorry"*
> *(Workshop 5, Patient 1)*

During the pharmacist workshops some frustration was communicated about not being allowed to provide certain products over the counter (Table 6), products that customers would subsequently receive on prescriptions for from their GPs:

> *"I do recognize some or quite a few skin conditions, I would like to give them something that is prescription only, but I can't. So then I have to send them off to the GP, so I would personally like some sort of PGD [Patient Group Direction] or guidelines to be able to prescribe [erm], to do a course be accredited and to be able to prescribe that maybe not to have to go through the whole performance of becoming an independent prescriber, because I don't have the time or the facility to do that."*
> *(Workshop 4, Pharmacist 6)*

For peer review only

Table 6 Further quotes to support the research questions derived from Theme 5 - Resources to support the management of skin conditions and improving access to medications

Management of skin conditions	*I just always find it difficult with eczema, just the amount of emollients, and it's what emollients to recommend and what circumstance. Pharmacist*
Providing a wider range of treatments	*"I find it difficult to treat over the counter because I feel quite limited in what we're what we're licensed to sell and for what and for use for, like on on the areas of the body" Pharmacy dispenser*<br><br>*"I can't tell you how many referrals I've made for impetigo because there's just nothing to offer" Pharmacist*
Access to topical corticosteroids	*"And as someone mentioned, you have to be quite careful of what you say to them in order to get it [TCS]. " Patients*<br><br>*"I might treat something off license and and pharmacists are very much by the book. But in dermatology, we can't always do things by the book and we have to use things off licence because sometimes there's just nowhere else to go. I mean, you know, I for years, I I had a paediatric eczema clinic. And I just felt so sorry for those parents who, even with the GP, found it difficult to to get the medication that they needed if their children's skin was flaring and they had quite severe flares and they had to wait to come and see us and we only had it clinic once once a week." Specialist dermatology nurse*<br><br>*"[the patient said] I've forgotten my cream and they wanted to buy hydrocortisone cream and they said I've got eczema on my face, and you simply can't sell it. And and and you get into it. So so in many respects you you've got kind of silly restrictions like that which actually impede your ability to practice properly because you think because you effectively look a bit stupid because, well, the doctor gives it to me and you're saying you can't sell it to me and you think what is all that about?" Pharmacist*<br><br>*"I'm not concerned about pharmacists supplying the items inappropriately, but it's the whole over the counter consultation because this my staff know that the face is a no go zone for hydrocortisone. So if somebody was requesting it for the face, that's a hard kind of stop for them to come and grab me to have a conversation with the patient about this. Whereas if that licensing was removed, you may run* the risk *of just missing these patients throughout the whole pharmacy team." Pharmacist*

For peer review only

Some members of the public were equally frustrated by this, confused about why they could order medications from the internet but not access them directly via community pharmacies. Topical corticosteroids (TCS) were often discussed in this way in the workshops as well as in some of the one-to-one healthcare professional interviews. Members of the public described how they had lied about how they were going to use TCS to ensure that it was provided (Table 6):

> *"the, the only time it was mentioned [topical corticosteroids] was when they refused to sell me it, you know, and that that that sounds stupid. It was, you know, when I've got it on prescription, there's never been any query or any conversation about it. It's just been given in a bag. [And] But when I needed to actually purchase something over the counter. And that's when the interrogation started."*
>
> *(Workshop 5, Patient 2)*

The centrality of product knowledge to the role of the community pharmacist suggests that it could be an important area for research and resource development. The potential to extend what pharmacists can do may be an important in this.

> Possible research question - Could a wider range of products and treatments for skin conditions be made available via community pharmacy?

> Possible research questions - Would dedicated resources support community pharmacists in the management of skin conditions?

Theme 6 – Other topics

Discussion of the themes identified in the online survey often prompted a broader discussion of community pharmacy and skin conditions. This led to the identification of additional areas where research might be warranted.

In the workshops pharmacists reinforced the notion that they see a broad range of skin conditions daily, and that the number of customers seeking advice about skin conditions is increasing (both because of Covid 19 as well as the Community Pharmacy Consultation Service [CPCS]) (Table 7).

> *"I think the numbers of skin referrals with the CPCS is going to go up into Community pharmacy because it's one thing that the GPs can triage without seeing."*
>
> *(Workshop 4, Pharmacist 7)*

For peer review only

Table 7 Further quotes to support Research Question from Theme 6 - Current involvement of pharmacists and the known benefits of the involvement of community pharmacists in the identification and management of skin conditions

Assessment of rising demand	*"And I see such a broad range [..] I just see a bit of everything all the time" Pharmacist*<br><br>*"but I do get a lot of people coming in children with children. All sorts of skin conditions. As you said ringworm and eczema and slap cheek." Pharmacist*<br><br>*"I think it has [consultations for skin disease] increased, but I was still seeing quite a few skin conditions, so people coming in asking before COVID as well asking what it might be and anything [I] could recommend." Pharmacist*<br><br>*"I think it will, I think it will stay [the increased demand], UM I, I think. [It's] the ease of access you know? And because people are not getting appointments with GP" Pharmacist*
Variation in service provisions	*"I think the main ailments aspect needs to be stretched. To cover all areas of the country to make it easier for pharmacies in areas that don't have it. Because some of the things that we can't assist with are ridiculous." Pharmacy dispenser*<br><br>*"[the geographical area] has some PGDs that allow for supply, for example, hydrocortisone on the face under PGD" Pharmacist*<br><br>*"You know, and if I've got the choice of trying to get an appointment to see a GP. And then they'll give me a prescription, and then because I've got a prepayment, I don't have to pay for it or going to a chemist and then charging me £9 for a bottle of Double Base Ointment. The logic says you go to the GP first. You've just saved yourself. You know, a tenner" Patient*<br><br>*"I'm not a massive fan of the CPCS thing yet, cause UM our area doesn't do much minor ailments and they're expecting things for free. And I'm, they're like, no, I can't. I can't give you that you're gonna have to. You're gonna have to pay." Pharmacist*<br><br>*" How well do you trust somebody's clinical judgment against their commercial need?" Patient*

For peer review only

Pharmacists, however, were less confident in making an assessment about how demand is growing and evolving: e.g. which clinical conditions, what types of enquiry, specific demographic groups, adults/children, etc. Discussion in the workshops, as well as some one-to-one interviews, recognised that understanding trends in demand could be an important precursor to any substantive change in how community pharmacy works or engages with skin conditions.

Possible research question - In what ways are community pharmacists currently involved in the identification and management of skin conditions?

Some of these discussions (especially in the pharmacist workshop) exposed current localised variation in what is available and what pharmacists are allowed to do.

"We're lucky like, in England, as you have heard you saying that you guys have to charge your folk for it. In the pharmacy first, one thing we've got, is we can give it out free of charge and they can come back six times and get six different bottles and it doesn't cost them anything"
(Workshop 2, Pharmacist 1)

Discussion demonstrated that variations are manifest in: (i) the skin conditions that pharmacists are paid to treat via minor ailments schemes; (ii) the medications which can be provided via the use of patient group directions (PGDs); and, (iii) whether patients had to pay for their treatment (Table 7). As with understanding trends in demand it was considered pertinent to develop a better understanding of the success of current skin focused initiatives and ways of working as a precursor to any further development.

Possible research question - What are the known benefits of community pharmacy involvement in the identification and management of skin conditions?

The public workshops offered a slightly different perspective on this topic, focusing upon establishing that pharmacists are appropriately qualified and competent to deal with skin conditions.

"It just feels that pharmacists know a lot about their medicines and of course they know the pros and cons of uses, but whether they have got the expertise in recognizing a particular type of rash or a particular type of mark on the skin […] I wouldn't know if they had that expertise."
(Workshop 7, Patient 3)

Similar concerns were expressed in the healthcare professional interviews, with one GP indicating that they saw "a lot of inappropriate" referrals from pharmacists (Table 8).

For peer review only

Table 8 Further quotes to support Research Question 9. and 10. Competency of community pharmacists in managing skin conditions and awareness of the public of the skills of community pharmacists with regards to the identification and management of skin conditions.

Pharmacist competency in the identification and treatment of skin conditions	*"I would guess a certificate on the wall […] go to my optician and he's got all his different specialities on on the wall. So something just with simple as that and I guess they would have to be trained by a consultant dermatologist or at least somebody with many years of experience at spotting all kinds of different conditions." Patient*<br><br>*"I think one I don't know what community pharmacists know, and I think that's part of the problem. We work very closely with community pharmacists in some ways, but we're very, very separate in others, so we phone them up about medication queries and they phone us all the time. But actually knowing what each other's skills are is is quite difficult." GP*<br><br>*"But what I do see is what they've attempted to treat and then comes to us anyway, so I see quite a lot of misdiagnoses from community pharmacies, so a lot of insect bites that look very red and inflamed are sent to us because they think it's infected […] we see lots of sort of cold sore sent to us as query impetigo needing antibiotics, lots of eczema that's just red and inflamed might be infected and gets sent to us and it's not, it just needs eczema treatment so I think. I I see some fairly basic areas of of lacking in knowledge […] but what I don't see are the ones that they have successfully treated and and and sent away so I don't know what sort of ratio we're talking here." GP*
Raising awareness of pharmacy skin services	*"I've had various sort of short term skin conditions and pharmacies have always been my sort of first port of call. It [has] always been good experiences" Patient*<br><br>*"I never knew the pharmacy that they could advise you on skin you know problems, I never knew that. Because I thought they dealt dealt with drugs only and they all seemed very busy. So I'd like to know how the pharmacist can help with skin conditions as well?" Patient*<br><br>*"This minor ailment scheme and for that it needs the patient consent, so we need them to reply to the text message or we need to speak to them on the phone to get their consent and then make the referral, which is a very quick process, but it's all quite clunky and it doesn't always have the patient buy in because they, sometimes incorrectly, value the GP's skills more than the community pharmacists skills, so there needs to be a degree of sort of selling of this and patient education" GP*

For peer review only

Once again the benefit of establishing the state of current provision for skin conditions in community pharmacy was recognised as an appropriate focus.

> Possible research question - How competent are community pharmacists in the identification and management of skin conditions?

Discussion of direct experience of accessing community pharmacy exposed that participants in the public workshops were often polarised, between those that regularly used their pharmacist for help and advice and those that were not aware that pharmacists offered this type of service.

> *"I never knew that they could advise you on skin you know problems, I never knew that. Because I thought they dealt dealt with drugs only and they all seemed very busy. So, I'd like to know how the pharmacist can help with skin conditions as well?"*
> *(Workshop 6, Patient 4)*

A concern about a lack of awareness about pharmacy services was echoed in the healthcare professional interviews, where GPs described difficulties convincing patients that minor ailments schemes are appropriate to use (Table 8). Identifying barriers and encouraging the use of community pharmacy might be important research that underpins the effectiveness of any specific initiative:

Possible research question - What could be done to raise awareness of the skills that community pharmacists have with regards to the identification and management of skin conditions?

A list of all ten research questions are provided in Table 9 (see online Supplementary File 3 for list of list of research questions and explanatory notes).

For peer review only

Table 9 The ten research questions from the community pharmacy and dermatology priority setting partnership (in no particular order, prioritised questions in bold)

1. **Would dedicated resources improve the identification of skin conditions in community pharmacy?**
2. Would dedicated resources improve the identification of skin conditions in skin of colour in community pharmacy?
3. Would dedicated resources support community pharmacists to effectively refer skin conditions that require urgent or more specialist attention?
4. **How can community pharmacists work most effectively with other healthcare professionals in the identification and management of skin disease?**
5. Could a wider range of products and treatments for skin conditions be made available via community pharmacy?
6. Would dedicated resources support community pharmacists in the management of skin conditions?
7. In what ways are community pharmacists currently involved in the identification and management of skin conditions?
8. What are the known benefits of community pharmacy involvement in the identification and management of skin conditions?
9. **How competent are community pharmacists in the identification and management of skin conditions?**
10. What could be done to raise awareness of the skills that community pharmacists have with regards to the identification and management of skin conditions?

For peer review only

Stage 3 - prioritisation workshops

To accommodate all those interested in participating, the prioritisation workshop was split into two parts which ran consecutively. The first prioritisation workshops included one dermatology nurse specialist and 10 patients. The second prioritisation workshop included three pharmacists, two members of pharmacy staff and 13 patients (Table 2). Scores at the end of the first workshop were carried forward as the starting point for the second.

At the conclusion of the second workshop the following questions were voted to be the most important (3 participants did not vote):

1. How can community pharmacists work most effectively with other healthcare professionals in the identification and management of skin disease? 11/15 participants
2. How competent are community pharmacists in the identification and management of skin conditions? 9/15 participants
3. Would dedicated resources improve the identification of skin conditions in community pharmacy? 6/15

DISCUSSION

Summary

Through consultation with a range of stakeholders including pharmacists, pharmacy staff, GPs, dermatology nurses and members of the public we have developed a set of research questions to support dermatology provision in community pharmacy. In a final prioritisation exercise we have established three topics as a starting point for a dermatology/community pharmacy research agenda.

Discussion in our workshops reinforced existing assessment that dermatology is a significant part of the workload faced by community pharmacists [12,14,22]. It also reinforced the expectation that this demand is likely to grow in future. Concerns about limited training and knowledge about skin conditions [1] were evident in comments about lacking confidence in dealing with skin queries. A perceived lack of resources, training and post-consultation feedback were recognised as factors in this. Developing resources for pharmacists/pharmacy staff might be an important area where research can benefit pharmacy practice – resources in this context might be training programmes, information resources for staff, information resources for the public, and they could be delivered in print, in person or online.

Research uncertainties identified here might suggest the value of repeating and/or expanding prior research which has considered pharmacists' ability to identify skin conditions [16,17].

Our findings also reinforce more general issues of public awareness about the role of community pharmacy [23,24], specifically recognising this to be an issue with regards to skin conditions. Previous work has identified that individuals felt that only doctors are "qualified/trustworthy" to manage skin complaints [11], a view also expressed by medicine counter assistants[1]. This is a particularly important challenge to negotiate given that all stakeholders who took part in the exercise recognised that the demand in primary care exceeds what GPs are able to manage. However, as there is currently a shortfall in the number of pharmacists and pharmacy staff, it is possible that, under the current workforce model pharmacists may also struggle to meet this demand[25].

For peer review only

Further information about the questions

We have developed a broad range of questions reflecting a broad range of concerns described in the workshops.

We have framed questions loosely and in general terms to allow interpretation and wide scope for impact. For example, the final question, "*How can community pharmacists work most effectively with other healthcare professionals in the identification and management of skin disease?*" could be approached in terms of consideration of the appropriate prescribing of "over the counter" medicines or in terms of the provision of information for regarding the use of long-term prescription medications.

In this, however, we might suggest that there is a form of natural hierarchy with questions focused upon understanding current provision a necessary precursor to research which develops new ways of working. For example, answering, *"in what ways are community pharmacists currently involved in the identification and management of skin conditions?",* would allow future work to be appropriately directed to the most common or most difficult to manage skin conditions.

We would encourage researchers to develop the focus of research in meaningful ways, mindful of the changing landscape within community pharmacy with initiatives such as the independent prescribing schemes in England[3].

Strengths and limitations

This has been a broad reaching exercise which has included 111 community pharmacists in an online survey as well as 57 workshop and interview participants. Workshops sought input from both healthcare professionals as well as members of the public – numbers were approximately even in the exploratory workshops (27 healthcare professionals / 30 members of the public).

Whilst we are confident that a broad range of perspectives were considered in the identification of research topics the final prioritisation stage was heavily weighted towards the input of members of the public. At this stage of our process the goal of equal numbers was not achieved, with only five pharmacists/pharmacy staff participating in the prioritisation stage (despite twelve signing-up). Further work in this area could explore different methods of engaging with GPs and pharmacists to improve recruitment. We should consequently recognise that the final prioritisation more accurately reflects the public view of what research would be most beneficial, rather than a multi-perspective assessment of this.

CONCLUSION

Pharmacists are regularly consulted regarding skin conditions and do not always feel confident in the identification of skin disease. Using information from focus groups with pharmacists, members of the public and other stakeholders we developed 10 research questions that can be used to direct future research to address these challenges.

For peer review only

ETHICAL APPROVAL STATEMENT

Ethical approval for the survey was obtained from Aston University Research Ethics committee. The Faculty of Medicine & Health Sciences Research Ethics Committee, at the University of Nottingham, confirmed that Ethical approval was not required for the workshops as the research was an engagement activity to inform future research priorities.

FUNDING STATEMENT

This project is funded by the National Institute for Health and Care Research (NIHR) School for Primary Care Research (project reference 522).

The views expressed are those of the author(s) and not necessarily those of the NIHR or the Department of Health and Social Care.

CONFLICTS OF INTEREST

JH, IM, CA, KST, PL, ZS have declared they have no conflict of interest.

MB has received personal fees from Delphi Healthcare outside the submitted work, has grants or contracts with Health Education England (Grant for development of experimental learning activity)and Walgreen Boots Alliance (50% funding for PhD studentship). MB has received consulting fees from Clinical Care Quality Solutions (payment split between institution and author). MB has received payment from the Ministry of Health Singapore, Human Manpower Development programme (honoraria to speak including travel and accommodation). MB is a Project advisor/Chair of experiential learning during the MPharm research project. NHS Scotland (payment to institution and author). MB is Vice Chair Pharmacy Law and Ethics Association (no fee received).

MS has the following Grants or contracts:

RAPID and Efficient Eczema Trials (RAPID programme) – lead applicants Thomas and Roberts. NIHR PGfAR NIHR203279 funding to University of Southampton for 5% of my time.

Trial of IGe tests for Eczema Relief (TIGER): randomised controlled trial of test-guided dietary advice for children with eczema, with internal pilot and nested economic and process evaluations – lead applicant Ridd. NIHR HTA NIHR133464 funding to University of Southampton for 10% of my time

Pragmatic, primary care, multi-centre, randomised superiority trial of four emollients in children with eczema, with internal pilot and nested qualitative study (Best Emollients for Eczema - BEE) – lead applicant Ridd. NIHR HTA 15/130/07 completed Aug 2020 funding to University of Southampton for 10% of my time.

MS is a Funding panel member NIHR Programme Grants for Applied Research 2018 to present day and also Academic PPIE lead and Board Member NIHR School for Primary Care Research 2022 to present day.

MR has received Various NIHR grants for studies of skin conditions/food allergy. MR is On TSC/DMC for ERICA, PRINCIPLE and ALPHA trials and is Co-Chair SAPC & NIHR SPCR skin/allergy research groups.

CONTRIBUTIONS

ZS and IM conceptualised, designed and conducted the survey, ZS and JH analysed the survey data, JH and PL led the workshops and interviews, analysis of the interview and

For peer review only

workshop data and drafted the manuscript, comments were made on the draft by ZS, IM, MS, MR, MB and CA All authors commented on and approved the final draft.

DATA SHARING STATEMENT

Data is available from the authors on request.

For peer review only

REFERENCES

1. Tucker R, Stewart D. An exploratory study of the views of community pharmacy staff on the management of patients with undiagnosed skin problems. *Int J Pharm Pract* 2015;23(6):390-98. doi: 10.1111/ijpp.12179
2. Tucker R, Stewart D. Why people seek advice from community pharmacies about skin problems. *Int J Pharm Pract* 2015;23(2):150-53. doi: 10.1111/ijpp.12126
3. Medicines and Pharmacy Directorate - Department of Health and Social Care. The Community Pharmacy Contractual Framework for 2019/20 to 2023/24: supporting delivery for the NHS Long Term Plan. London2019 [Available from: https://assets.publishing.service.gov.uk/government/uploads/system/uploads/attachment_data/file/819601/cpcf-2019-to-2024.pdf accessed April 2022.
4. Delgutte A, Santos D. Community pharmacy has been an indispensable element of the covid-19 response BMJ online; [Available from: https://blogs.bmj.com/bmj/2021/02/21/community-pharmacy-has-been-an-indispensable-element-of-the-covid-19-response/#:~:text=Community%20pharmacy%20has%20been%20an%20indispensable%20element%20of%20the%20covid%2D19%20response,-February%2021%2C%202021&text=Community%20pharmacists%20are%20trained%20to,rest%20of%20the%20healthcare%20system accessed April 2022.
5. NHS England. NHS Community Pharmacist Consultation Service (CPCS) – integrating pharmacy into urgent care [Available from: https://www.england.nhs.uk/primary-care/pharmacy/pharmacy-integration-fund/community-pharmacist-consultation-service/ accessed October 2022.
6. The King's Fund. How can dermatology services meet current and future patient needs while ensuring that quality of care is not compromised and that access is equitable across the UK? . London, 2014.
7. Le Roux E, Edwards PJ, Sanderson E, et al. The content and conduct of GP consultations for dermatology problems: a cross-sectional study. *British Journal of General Practice* 2020;70(699):e723-e30. doi: 10.3399/bjgp20X712577
8. Schofield J, Grindlay D, Williams H. Skin conditions in the UK : a health care needs assessment: Centre of Evidence Based Dermatology Nottingham, 2009.
9. Eedy D. The crisis in dermatology. *BMJ* 2015;350:h2765. doi: 10.1136/bmj.h2765
10. Fielding S, Porteous T, Ferguson J, et al. Estimating the burden of minor ailment consultations in general practices and emergency departments through retrospective review of routine data in North East Scotland. *Fam Pract* 2015;32(2):165-72. doi: 10.1093/fampra/cmv003
11. Hammond T, Clatworthy J, Horne R. Patients' use of GPs and community pharmacists in minor illness: a cross-sectional questionnaire-based study. *Fam Pract* 2004;21(2):146-9. doi: 10.1093/fampra/cmh207
12. Tucker R. Community pharmacists' perceptions of the skin conditions they encounter and how they view their role in dermatological care. *Int J Pharm Pract* 2012;20(5):344-6. doi: 10.1111/j.2042-7174.2012.00212.x
13. Tucker R. An exploratory study of uk community pharmacist's perceptions of the skin conditions they encounter: prevalence, reasons for referral and over-the-counter dermatological wish list. *Selfcare* 2013;4(1):3-17.
14. Dodds L, Katusiime B, Shamim A, et al. An investigation into the number and nature of the urgent care consultations managed and referred by community pharmacists in South-East England. *Prim Health Care Res Dev* 2020;21:e5. doi: 10.1017/S1463423620000031
15. Rubsam ML, Esch M, Baum E, et al. Diagnosing skin disease in primary care: a qualitative study of GPs' approaches. *Fam Pract* 2015;32(5):591-5. doi: 10.1093/fampra/cmv056
16. Tucker R, K. M, V. P, et al. An exploratory study of community pharmacist diagnosis and management of dermatitis and acne. *Selfcare* 2017;8(2):1-10.
17. Tucker R, Patel M, Layton AL, et al. An exploratory study demonstrating the diagnostic ability of healthcare professionals in primary care using online case

studies for common skin conditions. *Int J Pharm Pract* 2014;22(2):119-24. doi: 10.1111/ijpp.12045
18. Nygaard A, Halvorsrud L, Linnerud S, et al. The James Lind Alliance process approach: scoping review. *BMJ Open* 2019;9(8):e027473. doi: 10.1136/bmjopen-2018-027473
19. Hsieh HF, Shannon SE. Three approaches to qualitative content analysis. *Qual Health Res* 2005;15(9):1277-88. doi: 10.1177/1049732305276687
20. Gale NK, Heath G, Cameron E, et al. Using the framework method for the analysis of qualitative data in multi-disciplinary health research. *BMC Med Res Methodol* 2013;13:117. doi: 10.1186/1471-2288-13-117
21. Delbecq AL, Van de Ven AH. A Group Process Model for Problem Identification and Program Planning. *The Journal of Applied Behavioral Science* 1971;7(4):466-92. doi: 10.1177/002188637100700404
22. Hafejee A, Coulson IH. Community pharmacists' role in managing common skin problems. *Br J Dermatol* 2006;155(6):1297. doi: 10.1111/j.1365-2133.2006.07545.x
23. Latif A, Waring J, Watmough D, et al. Examination of England's New Medicine Service (NMS) of complex health care interventions in community pharmacy. *Res Social Adm Pharm* 2016;12(6):966-89. doi: 10.1016/j.sapharm.2015.12.007
24. Latif A, Waring J, Watmough D, et al. 'I expected just to walk in, get my tablets and then walk out': on framing new community pharmacy services in the English healthcare system. *Sociol Health Illn* 2018;40(6):1019-36. doi: 10.1111/1467-9566.12739
25. Company Chemists Association. Review of the Community Pharmacist Workforce in England 2022 [Available from: https://thecca.org.uk/cca-publishes-review-of-the-community-pharmacist-workforce-in-england/ accessed December 2022.

For peer review only

For peer review only

COREQ checklist

Item number		Notes
1	Which author/s conducted the interview or focus group?	All workshops and interviews were facilitated by both JH and PL.
2	What were the researcher's credentials? E.g. PhD, MD	JH has a Ph.D and MPharm. PL has a Ph.D
3	What was their occupation at the time of the study?	JH and PL work at the Centre of Evidence based Dermatology, School of Medicine, University of Nottingham.<br>JH is a registered pharmacist.
4	Was the researcher male or female?	N/A
5	What experience or training did the researcher have?	PL has extensive expertise in qualitative methods.<br><br>JH has completed several pieces of qualitative work examining pharmacist led services.
6	Was a relationship established prior to study commencement?	Researchers did not establish a relationship with participants prior to study commencement
7	What did the participants know about the researcher? e.g. personal goals, reasons for doing the research	Participants did not know that JH was a registered pharmacist. They were aware both researchers worked for the Centre of Evidence Dermatology.
8	What characteristics were reported about the inter viewer/facilitator? e.g. Bias, assumptions, reasons and interests in the research topic	We have acknowledged in the write-up that JH has a background as a pharmacist
9	What methodological orientation was stated to underpin the study? e.g. grounded theory, discourse analysis, ethnography, phenomenology, content analysis	This was exploratory, consultative research broadly informed by the James Lind Priority Setting Partnership approach.<br><br>See methods section, line 173, 179, 183, 187
10	How were participants selected? e.g. purposive, convenience, consecutive, snowball	See methods section line 143
11	How were participants approached? e.g. face-to-face, telephone, mail, email	Line 145,152
12	How many participants were in the study?	Table 1
13	How many people refused to participate or dropped out? Reasons?	Although the first focus groups were attended by 19 pharmacists, the final online consensus workshop was poorly attended by

		pharmacists and members of pharmacy staff. We had initially invited an equal number of pharmacists and pharmacy staff (12) and members of the public (13) but only 5/12 pharmacists and members of pharmacy staff, who were signed up prior to the meeting, attended the meeting.<br><br>Reasons (where provided) for nonattendance included sickness and that they were too busy.
14	Where was the data collected? e.g. home, clinic, workplace	Line 177
15	Was anyone else present besides the participants and researchers?	No
16	What are the important characteristics of the sample? e.g. demographic data, date	We did not collect any demographic data
17	Were questions, prompts, guides provided by the authors? Was it pilot tested?	Workshops used the findings of earlier stages of the research as their focus.<br><br>Data analysis and progress summaries were considered by the wider author group and PPI representatives prior to the workshops.
18	Were repeat inter views carried out? If yes, how many?	N/A
19	Did the research use audio or visual recording to collect the data?	Line 179
20	Were field notes made during and/or after the interview or focus group?	No fieldnotes were made during or after data collection.
21	What was the duration of the inter views or focus group?	Line 219
22	Was data saturation discussed?	Data saturation was not a concept applied to this research, rather data collection
23	Were transcripts returned to participants for comment and/or correction?	No
24	How many data coders coded the data?	PL and JH coded the data. Other authors reviewed coded data and confirmed appropriateness of interpretations.
25	Did authors provide a description of the coding tree?	Yes Table 2

For peer review only

26	Were themes identified in advance or derived from the data?	Initial themes were derived from the inductive content analysis of the online survey. Themes were refined following the exploratory workshops.
27	What software, if applicable, was used to manage the data?	NVivo ver 12 was used to handle research data.
28	Did participants provide feedback on the findings?	No
29	Were participant quotations presented to illustrate the themes/findings? Was each quotation identified? e.g. participant number	Yes, identified by participant number and profession and by workshop number
30	Was there consistency between the data presented and the findings?	Yes findings were based on an analytic process that was iterative.
31	Were major themes clearly presented in the findings?	Yes in the form of the 10 questions
32	Is there a description of diverse cases or discussion of minor themes?	Yes where applicable in the text

For peer review only

Introduction

The responses from two open questions from the Aston Pharmacy Dermatology Survey have been analysed.

We have:

- Identified the top 20 words from the two open questions; the questions concerned are the “challenge” question and the “research priority” question.
- Created word trees from the most common words identified, then used thematic analysis of the word trees to draw up the long list of questions.

Please see below for an example of the analyses mentioned above.

For peer review only

Challenge word analysis

Challenge question: Please list at least one and up to two of the most challenging aspects of treating or managing patients presenting with skin conditions. For example: "How can community pharmacy best support safe use of topical corticosteroids?" or "How do we know when to refer someone with a mole?"

Number of challenge word responses=348

Top 20 words from the challenge question

Word	Count	Similar words
**refer**	44	refer, referal, referred, referring
**rash**	39	rash, rashes
**products**	26	product, products
**differential**	25	differential, differentials, differentiate, differentiating
**know**	24	know, knowing
**diagnosis**	22	diagnosis
**different**	21	difference, differences, different, differently
**steroid**	19	steroid, steroids
**need**	16	need, needed, needing, needs
**support**	16	support, supporting
**treatment**	16	treatment, treatments
**creams**	15	cream, creams
**infected**	15	infected, infection, infections
**time**	15	time, timely, times
**limited**	14	limitations, limited, limits
**difficult**	14	difficult
**emollients**	14	emollient, emollients
**identify**	14	identify, identifying
**treat**	14	treat, treated, treating
**mole**	14	mole, moles

For peer review only

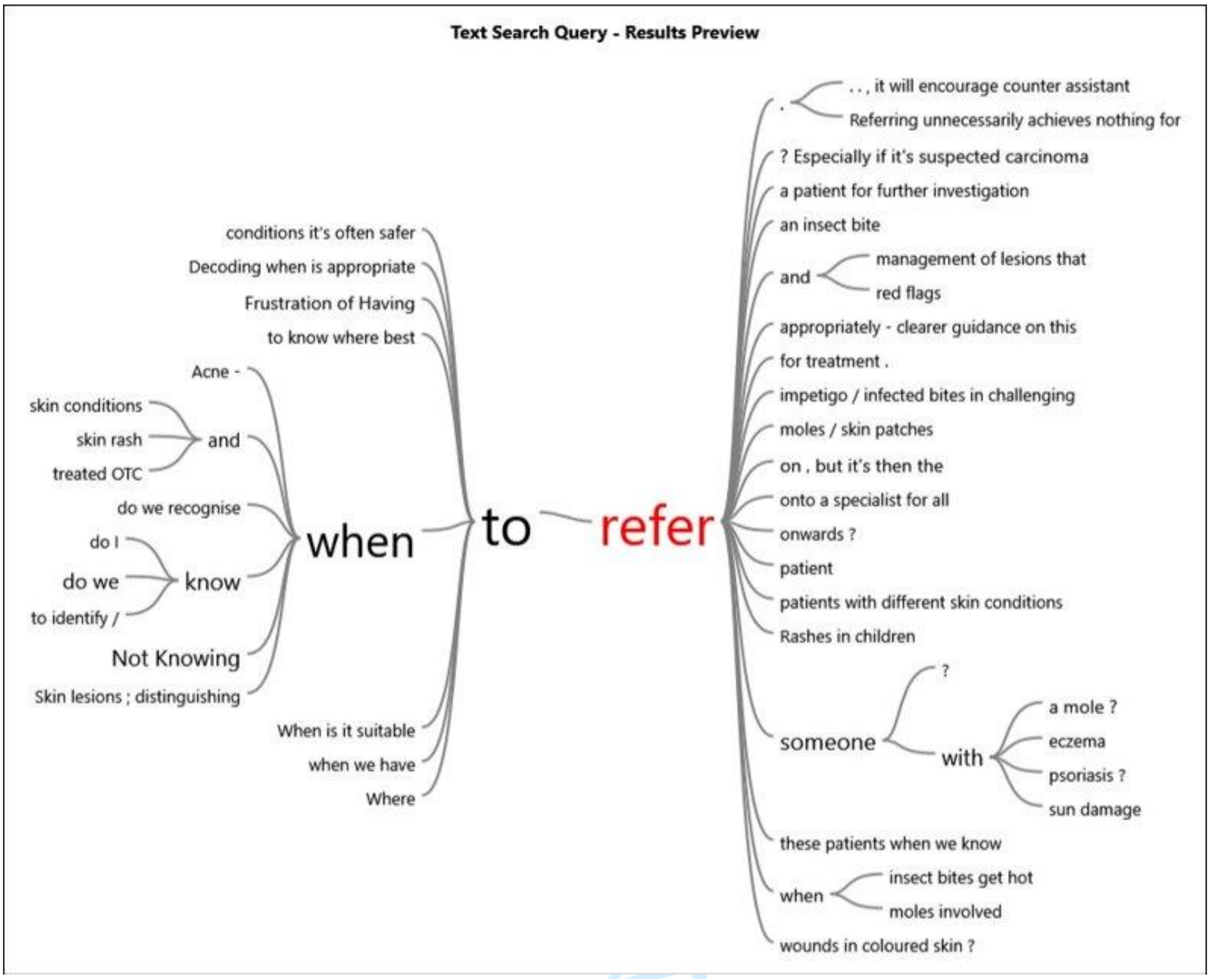
Text Search Query - Results Preview
conditions it's often safer
Decoding when is appropriate
Frustration of Having
to know where best
Acne -
skin conditions
skin rash
treated OTC
and
do we recognise
do I
do we
to identify /
know
Not Knowing
Skin lesions ; distinguishing
when
When is it suitable
when we have
Where
to
refer
. . , it will encourage counter assistant
Referring unnecessarily achieves nothing for
? Especially if it's suspected carcinoma
a patient for further investigation
an insect bite
and
management of lesions that
red flags
appropriately - clearer guidance on this
for treatment .
impetigo / infected bites in challenging
moles / skin patches
on , but it's then the
onto a specialist for all
onwards ?
patient
patients with different skin conditions
Rashes in children
someone
?
with
a mole ?
eczema
psoriasis ?
sun damage
these patients when we know
when
insect bites get hot
moles involved
wounds in coloured skin ?

Research priority analysis question

Please list at least one and up to three of your top research priorities (e.g.what do we need to know more about) in relation to the role of community pharmacy in skincare (consider both advice/counter prescribing and advice on prescription medications).

Number of research priority responses - 522

Top 20 words – research priority questions

Word	Count	Similar words
**treatment**	47	treatment, treatments
**different**	32	difference, different, differing
**products**	31	product, products
**need**	28	need, needed, needing, needs
**creams**	24	cream, creams
**rashes**	22	rash, rashes
**care**	21	care
**steroid**	21	steroid, steroidal, steroids
**pharmacists**	20	pharmacist, pharmacists
**eczema**	19	eczema
**psoriasis**	18	psoriasis
**training**	18	train, trained, training
**prescribing**	17	prescribe, prescribed, prescriber, prescribers, prescribing
**diagnosis**	16	diagnosis
**information**	16	inform, information
**effects**	15	effecting, effective, effectively, effectiveness, effects
**acne**	15	acne
**advice**	15	advice
**infections**	15	infected, infection, infections
**referral**	15	referral

For peer review only

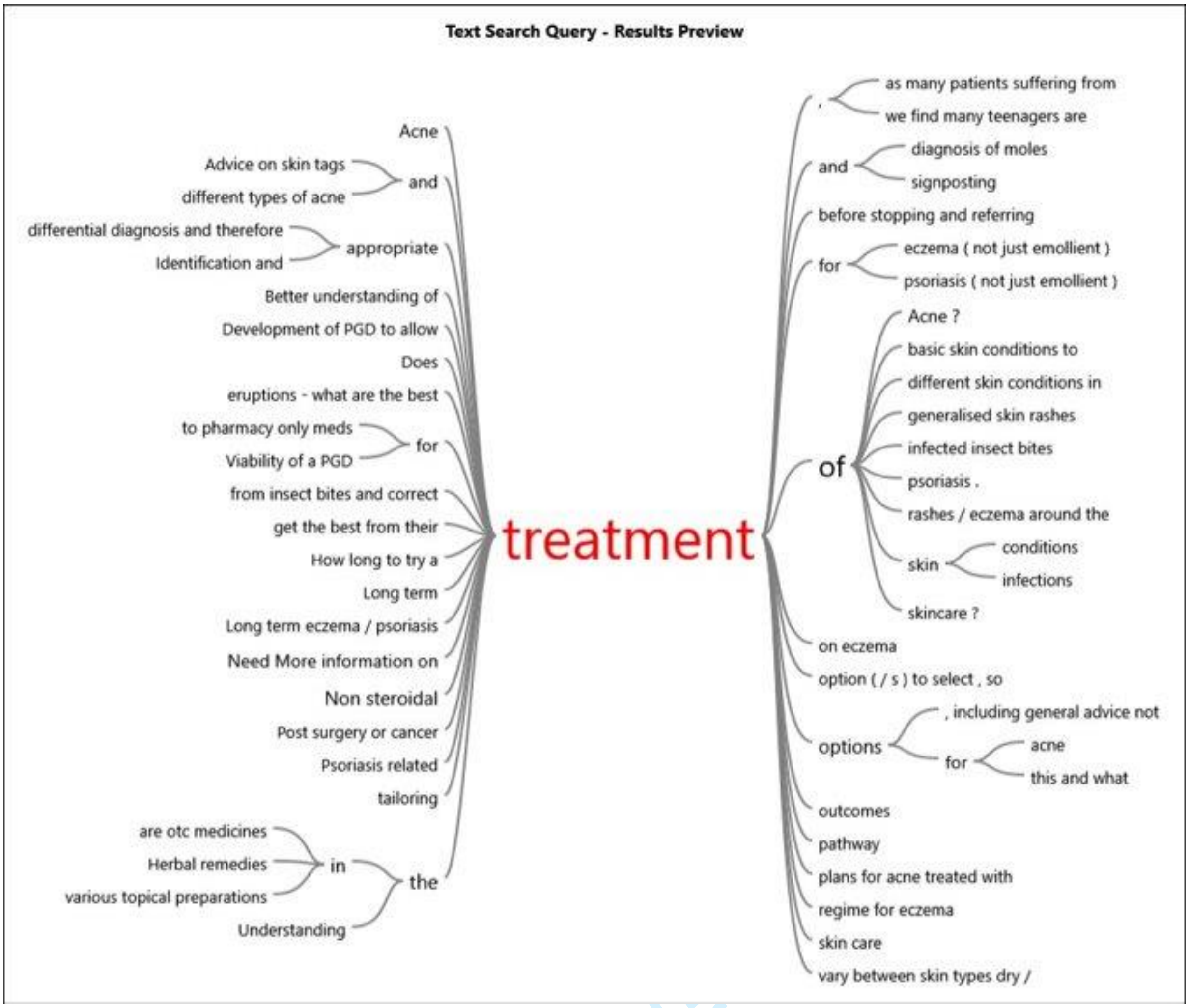

For peer review only

N.B. In the notes below, where we refer to community pharmacists, we include any appropriately qualified member of the community pharmacy team.

Question	Explanatory notes
1. What are the known benefits of community pharmacy involvement in the identification and management of skin conditions?	• This could be about establishing what is already known about community pharmacy involvement in the management of skin conditions.<br>• This could be important research which underpins the development of new services or resources.<br>• In our focus groups, participants described several different activities where community pharmacists have helped people with skin conditions. Some examples are these activities are; diagnosing, supporting self-management, recommending the use of products, and participating in dedicated medicines/illness schemes. Knowing which activities have evidence of being beneficial could support future provision.
2. In what ways are community pharmacists currently involved in the identification and management of skin conditions?	• This could be about developing a more detailed understanding about what currently happens in community pharmacy.<br>• It could include assessing levels of demand, mapping different types of activity, and identifying novel ways of working.<br>• In our focus groups it was apparent that services are developing differently across the country and that people were not aware of all that is currently happening in different areas.
3. How competent are community pharmacists in the identification and management of skin conditions?	• This could involve assessing the knowledge and skills of community pharmacists and other members of the pharmacy team.<br>• In our focus groups pharmacists described different levels of confidence and competence in addressing skin conditions. Members of the public were uncertain about how qualified community pharmacists are to manage skin conditions.
4. Would dedicated resources improve the **identification of skin conditions** by community pharmacists?	• This could involve developing and/or testing resources which support community pharmacists in identifying skin conditions. Resources might be online resources, training programmes etc.<br>• In our focus groups pharmacists described an absence of resources that they are in confident in using to identify skin conditions, they said that high quality resources could be useful in identifying skin conditions.
5. Would dedicated resources improve the **identification of skin conditions** in **skin of colour** by community pharmacists?	• This could involve developing and/or testing resources which support community pharmacists in identifying skin conditions in skin of colour.<br>• In our focus groups pharmacists described that identifying skin conditions in skin of colour is an additional challenge due to the different ways that skin conditions can look, and also because most current training/resources present images of white skin.
6. Would dedicated resources support community pharmacists in the **management** of skin conditions?	• This could involve developing and/or testing resources which support community pharmacists in managing skin conditions.<br>• In our focus groups pharmacists described being unsure about when they might take an active role in skin condition management. They also wanted

Question	Explanatory notes
	evidence-based information about what support and products they can provide.<br><br>• This might focus upon specific conditions (eczema, psoriasis, insect bites, etc.) or could be for more generic conditions (a rash, dry skin, etc.).
7. Would dedicated resources support community pharmacists to **effectively refer skin conditions** that require urgent or more specialist attention?	• This could involve developing and/or testing resources which support community pharmacists in referring skin conditions to other sources of healthcare (e.g. emergency care, general practice, etc.).<br><br>• It might include developing referral pathways and new services for specific serious skin conditions.<br><br>• In our focus groups pharmacists described feeling uncertain about when to take this sort of action; members of the public recognised that sometimes they wanted a pharmacist to 'tell them to go to the doctor'.
8. Could a wider range of products and treatments for skin conditions be made available via community pharmacy?	• This might include considering treatments which are restricted due to legal reasons (e.g. can only be supplied against a prescription) or economic reasons (e.g. where price might inhibit purchase).<br><br>• In our focus groups both pharmacists and members of the public described frustration that certain products are restricted and cannot be provided by a pharmacist.
9. What could be done to raise awareness of the skills that community pharmacists have with regards to the identification and management of skin conditions?	• This could involve local or national campaigns to raise awareness about community pharmacy as a healthcare resource. It might focus upon how community pharmacy services are understood by members of the public.<br><br>• In our focus groups some members of the public were not aware that community pharmacists are able to provide healthcare advice about skin conditions. Some members of the public were concerned that commercial concerns might be a priority for pharmacists, i.e. selling products might be more important than providing appropriate advice.
10. How can community pharmacists work most effectively with other healthcare professionals in the identification and management of skin disease?	• This might involve new integrated services for the management of skin conditions that include community pharmacists and other healthcare providers (e.g. GPs, nurses, dermatologists). It could include the use of pharmacist independent prescribers or pharmacists with additional training in dermatology.<br><br>• In our focus groups pharmacists reported working in isolation with little or no feedback about the advice and services that they offer. They also indicated that it is sometimes difficult to contact or engage with other healthcare providers about skin conditions.

For peer review only

BMJ Open

How can community pharmacists be supported to manage skin conditions? A multi-stage stakeholder research prioritisation exercise.

Journal:	*BMJ Open*
Manuscript ID	bmjopen-2023-071863.R1
Article Type:	Original research
Date Submitted by the Author:	15-Jun-2023
Complete List of Authors:	Harvey, Jane; University of Nottingham Centre for Evidence Based Dermatology<br>Shariff, Zakia; Aston University Aston Pharmacy School<br>Anderson, Claire ; University of Nottingham School of Pharmacy<br>Boyd, Matthew; University of Nottingham School of Pharmacy, School of Pharmacy<br>Ridd, Matthew; University of Bristol Faculty of Health Sciences, Population Health Sciences<br>Santer, Miriam; University of Southampton, Primary Care Research Centre,<br>Thomas, Kim; University of Nottingham Centre for Evidence Based Dermatology, Dermatology<br>Maidment, Ian; Aston University School of Life and Health Sciences, Aston Pharmacy School<br>Leighton, Paul; University of Nottingham Centre for Evidence Based Dermatology
**Primary Subject Heading**:	Dermatology
Secondary Subject Heading:	Health services research
Keywords:	DERMATOLOGY, Patient Participation, QUALITATIVE RESEARCH, PRIMARY CARE

SCHOLARONE™
Manuscripts

I, the Submitting Author has the right to grant and does grant on behalf of all authors of the Work (as defined in the below author licence), an exclusive licence and/or a non-exclusive licence for contributions from authors who are: i) UK Crown employees; ii) where BMJ has agreed a CC-BY licence shall apply, and/or iii) in accordance with the terms applicable for US Federal Government officers or employees acting as part of their official duties; on a worldwide, perpetual, irrevocable, royalty-free basis to BMJ Publishing Group Ltd ("BMJ") its licensees and where the relevant Journal is co-owned by BMJ to the co-owners of the Journal, to publish the Work in this journal and any other BMJ products and to exploit all rights, as set out in our licence.

The Submitting Author accepts and understands that any supply made under these terms is made by BMJ to the Submitting Author unless you are acting as an employee on behalf of your employer or a postgraduate student of an affiliated institution which is paying any applicable article publishing charge ("APC") for Open Access articles. Where the Submitting Author wishes to make the Work available on an Open Access basis (and intends to pay the relevant APC), the terms of reuse of such Open Access shall be governed by a Creative Commons licence – details of these licences and which Creative Commons licence will apply to this Work are set out in our licence referred to above.

Other than as permitted in any relevant BMJ Author's Self Archiving Policies, I confirm this Work has not been accepted for publication elsewhere, is not being considered for publication elsewhere and does not duplicate material already published. I confirm all authors consent to publication of this Work and authorise the granting of this licence.

For peer review only

Title: How can community pharmacists be supported to manage skin conditions? A multi-stage stakeholder research prioritisation exercise.

Proposed journal for submission: BMJ open

Authors: J Harvey[1#], Z Shariff[2], C Anderson[3], MJ Boyd[3], MJ Ridd[4] M Santer[5], KS Thomas[1], I Maidment[2] and P Leighton[1]

Affiliations

[1]Centre of Evidence Based Dermatology, School of Medicine, University of Nottingham, Nottingham, UK
[2] Aston Pharmacy School, Aston University, Birmingham, UK.
[3] Division of Pharmacy Practice and Policy, School of Pharmacy, University of Nottingham, Nottingham, UK
[4]Population Health Sciences, University of Bristol, Bristol, UK
[5] Primary Care Research Centre, University of Southampton, Southampton, UK
# Corresponding author jane.harvey1@nottingham.ac.uk Centre of Evidence Based Dermatology, Applied Health Research Building, School of Medicine, University of Nottingham, NG7 2RD

ORCiD

J Harvey 0000-0003-1402-6116
Z Shariff 0000-0003-4521-0864
C Anderson 0000-0002-5406-2296
MJ Boyd 0000-0003-2997-5090
MJ Ridd 0000-0002-7954-8823
M Santer 0000-0001-7264-5260
KS Thomas 0000-0001-7785-7465
I Maidment 0000-0003-4152-9704
P Leighton 0000-0001-5208-0274

Word count

Abstract 298

Body of text 4678

Acknowledgements

Thank you also to all the members of pharmacy staff, other healthcare professionals and members of the public that took part in interviews or workshops. Particular thanks goes to Amanda Roberts and our other patient representative for their help with this work.

For peer review only

Exclusive Licence

I, the Submitting Author has the right to grant and does grant on behalf of all authors of the Work (as defined in the below author licence), an exclusive licence and/or a non-exclusive licence for contributions from authors who are: i) UK Crown employees; ii) where BMJ has agreed a CC-BY licence shall apply, and/or iii) in accordance with the terms applicable for US Federal Government officers or employees acting as part of their official duties; on a worldwide, perpetual, irrevocable, royalty-free basis to BMJ Publishing Group Ltd ("BMJ") its licensees and where the relevant Journal is co-owned by BMJ to the co-owners of the Journal, to publish the Work in BMJ Open and any other BMJ products and to exploit all rights, as set out in our licence.

The Submitting Author accepts and understands that any supply made under these terms is made by BMJ to the Submitting Author unless you are acting as an employee on behalf of your employer or a postgraduate student of an affiliated institution which is paying any applicable article publishing charge ("APC") for Open Access articles. Where the Submitting Author wishes to make the Work available on an Open Access basis (and intends to pay the relevant APC), the terms of reuse of such Open Access shall be governed by a Creative Commons licence – details of these licences and which Creative Commons licence will apply to this Work are set out in our licence referred to above.

For peer review only

ABSTRACT

Objective

To establish research priorities which will support the development and delivery of community pharmacy initiatives for the management of skin conditions.

Design

An iterative, multi-stage stakeholder consultation consisting of online survey, participant workshops, and prioritisation meeting.

Setting

All data collection took place online with participants completing a survey (delivered via the JISC Online Survey platform, between July 2021 and January 2022) and participating in online workshops and meetings (hosted on Microsoft Teams between April and July 2022).

Participants

174 community pharmacists and pharmacy staff completed the online survey.

53 participants participated in the exploratory workshops (19 community pharmacists, 4 non pharmacist members of pharmacy staff and 30 members of the public). 4 healthcare professionals who were unable to attend a workshop participated in a one-to-one interview.

29 participants from the workshops took part in the prioritisation meeting (5 pharmacists/pharmacy staff, 1 other healthcare professional, and 23 members of the public).

Results

Five broad areas of potential research need were identified in the online survey:(1) identifying and diagnosing skin conditions; (2) skin conditions in skin of colour; (3) when to refer skin conditions; (4) disease specific concerns; and (5) product specific concerns.

These were explored and refined in the workshops to establish ten potential areas for research which will support pharmacists in managing skin conditions. These were ranked in the prioritisation meeting. Amongst those prioritised were topics which consider how pharmacists work with other healthcare professionals to identify and manage skin conditions.

Conclusions

Survey responses and stakeholder workshops all recognised the potential for community pharmacists to play an active role in the management of common skin conditions. Future research may support this in the generation of resources for pharmacists, in encouraging public take-up of pharmacy services, and in evaluating the most effective provision for dealing with skin conditions.

For peer review only

STRENGTHS AND LIMITATIONS

- Novel exploration of the research needs associated with the care of skin conditions within community pharmacy.
- An iterative, multi-stage consultation ensured detailed insight about the topic.
- The involvement of pharmacists, pharmacy staff, healthcare professionals and members of the public ensured that all pertinent voices were heard.
- Participants were self-selecting and may have had a particular interest/perspective upon skin conditions.
- Greater participation from pharmacists in the prioritisation workshops may have been beneficial.

For peer review only

INTRODUCTION

Community pharmacy is recognised as an accessible source of healthcare advice [1-3] and the COVID pandemic has cemented it more clearly in the primary care landscape for members of the public [4]. Moreover, recent initiatives, such as the Community Pharmacy Consultation Service (CPCS), seek to use pharmacy more effectively by diverting the management of some minor ailments to community pharmacy settings [5].

Skin conditions are amongst the most common diseases encountered by healthcare professionals[6,7]. Each year approximately 54% of the population will experience some form of skin disease[6], at any one time up to one-third of all people will have a skin condition that warrants medical attention[6,8,9]. Skin complaints have been identified as conditions that could be potentially managed within community pharmacy [10,11] and community pharmacists recognise skin conditions as a significant part of their workload [1,12,13]. Pharmacists regularly give advice on the management of common conditions such as eczema, dermatitis, generalised rashes, allergies and acne [12] and just over one-third (38%) of all symptomatic advice requests in community pharmacy relate to skin conditions[14]. Almost 20% of pharmacy sales are for skin products[2].

Due to the current stresses faced by the National Health Service, Community pharmacy in the U.K. is developing at a fast pace. Within England pharmacists are involved in treating dermatological conditions through the provision of a number of services. These include the CPCS, introduced in November 2020, where a GP surgery or NHS 111 can refer patients to community pharmacies for the treatment of minor illness, for example skin rashes[15]. Additionally, pharmacists may treat patients with skin conditions, free of charge, through minor ailments schemes but this provision varies in availability between areas[16]. In some areas specially trained pharmacists have access to prescription only medications through the use of patient group directions (PGDs) for certain conditions such as infected eczema or infected insect bites[17]. Most recently the government announced that a “Pharmacy First” scheme will be introduced within England. Through this scheme pharmacists will be able to prescribe medications (through PGDs) to treat conditions such as impetigo, shingles and infected insect bites[18]. In Wales and Scotland, the pharmacy first scheme has already been implemented. In these areas medications can also be provided via PGDs or through independent (non-medical) prescribers[19]. For example, in Scotland there are PGDs available for medications to treat impetigo, shingles and skin infections[20]. Therefore, within the UK context pharmacists are already involved in the diagnosis and treatment of skin conditions and this involvement has accelerated in the past few years. As community pharmacy continues its trajectory towards expanded and extended provision,[3] support for pharmacists in dealing with queries about skin conditions may be warranted. Skin conditions can be difficult to identify [21] and prior training for pharmacists in dermatological conditions is limited [6,8,22,23]. How best to support pharmacists is, however, unclear.

The aim of this work is to establish stakeholder consensus upon those research priorities which might best support community pharmacists in their involvement in the care of patients with skin conditions.

For peer review only

METHODS

This was a multi-stage, iterative stakeholder consultation informed by James Lind Priority Setting Partnership method [24] consisting of: (1) an online survey, (2) exploratory workshops, and (3) a prioritisation workshop.

Participants

Stage 1 – online survey

An online survey using the JISC Online Survey platform (https://www.jisc.ac.uk/online-surveys#) was targeted to community pharmacists and other community pharmacy staff. Social media (Twitter® and Facebook) and personal and professional networks (e.g., Pharmaceutical Services Negotiating Committee (PSNC) newsletter) were used to promote the survey. The survey was opportunistic and there were no specific inclusion criteria i.e. all pharmacists (and other members of pharmacy staff) were eligible to complete the survey.

A specific analysis of the survey data has been submitted for publication elsewhere.

Stage 2 – exploratory workshops

Community pharmacists, pharmacy staff, other healthcare professionals, and members of the public were recruited to a series of workshops to explore potential research topics which might support the management of skin conditions in community pharmacy.

Social media (Twitter® and Facebook) and personal and professional networks (e.g., Community Pharmacy Dermatology Network, primary care networks) were again used to recruit pharmacists, pharmacy staff as well as healthcare professionals (e.g., GPs, specialist nurse practitioners). Additional professional networks (the Primary Care Dermatology Society, the Society for Academic Primary Care Skin Special Interest Group and the UK Dermatology Clinical Trials Network) were used to recruit healthcare professionals.

Members of the public were recruited via social media and existing public and patient research networks (e.g. the CEBD patient panel and "People in Research" (https://www.peopleinresearch.org/)). All members of public who expressed an interest in the project were invited to join focus groups regardless of their experience of skin conditions or pharmacies.

Where any individual was not able to attend a scheduled workshop they were offered the opportunity to take part in a brief one-to-one interview.

Stage 3 – prioritisation workshops

Exploratory workshop participants were subsequently invited to take part in the prioritisation workshop.

Data collection and analysis

Stage 1 – online survey

Survey responses were collected over a period of six months between 20th July 2021 and 20th January 2022.

Content analysis of free text responses was used to identify commonly used words and phrases. Selected words or phrases (frequently used or substantively important) were reviewed thematically[25].

Stage 2 - exploratory workshops and interviews

Workshops and interviews were undertaken online using Microsoft Teams and took place between April and July 2022.

For peer review only

They were structured according to stage 1 data, with key themes explored further through group discussion. Discussion focused explicitly upon "research priorities"; although notions such as "barriers", "facilitators" and "challenges" were also used to make discussions less abstract and to support broad participation. See Supplementary files 1 and 2 for workshop schedules.

All discussions were digitally recorded with permission. Digital recordings were automatically transcribed verbatim and anonymised.

Framework analysis[26] was used to map workshop and interview data to broad uncertainties identified in stage 1. Synthesis of data and interpretation of synthesised data led to the creation of narrower research topics.

Stage 3 - prioritisation workshop

Following the conventions of the Nominal Group Technique[27] research topics were shared with participants prior to the prioritisation workshop. During the workshop group discussion and item scoring were used iteratively to reject and rank topics. Simple, descriptive statistics were used to rank and establish consensus upon priority research topics (i.e., the percentage of respondents selecting a topic for inclusion / priority). For further information regarding methods please see Supplementary file 3.

Patient and Public Involvement

Before the study started, we met with two PPI (patient and public involvement) collaborators to provide an overview of the study and the study methodology. One PPI member collaborated with us to develop the participant information leaflet. They also attended the steering group meeting where we developed the final list of research questions. The other PPI collaborator assisted with recruitment of patients via social media and recommended other areas where we could recruit participants e.g. the "people in research" website. They also attended one of our patient focus groups.

RESULTS

The numbers and characteristics of participants that took place at each stage of the process are shown in Table 1.

Table 1 Numbers of participants included at each stage of the priority setting exercise

	**Numbers of participants**		
	Stage One	Stage Two	Stage Three
**Type of participant**			
Patients	N/A	30	23
Pharmacists	111	19	3
Other members of pharmacy staff	63	4	2
Specialist dermatology nurse	N/A	1	1
GPs	N/A	3	0

Stage 1 – online survey

The survey was completed by 174 participants. Word counts and an example of the word trees are available in Supplementary file 4.

The most reported five words in response to the "challenge" questions were "refer, rash, products, differential and know" whilst in the "research priorities" question, the most reported words were "treatment, different, products, need and creams". These and the remaining top 20 words from each question encompass a broad range of research

For peer review only

challenges, which were reflected in the five key areas of the analytic framework detailed in Table 2.

Table 2 Original analytic framework developed from survey responses

Identifying and diagnosing skin conditions
Skin of colour
Knowing when to refer skin conditions to a GP
Disease specific concerns
Product specific concerns

Stage 2 – exploratory workshops and one to one interviews

Nine workshops were held, and four additional interviews to facilitate those unable to attend a scheduled workshop. Workshops lasted between one and two hours, interviews were typically around thirty minutes.

Four workshops consisted of pharmacists (19 participants), one included only pharmacy staff (4 participants), and four workshops contained only members of the public (30 participants). Interviews were undertaken with three GPs and one dermatology nurse specialist (Table 1).

Data is presented here thematically, pointing to key uncertainties and research possibilities that these themes suggest (further examples of the data are available in Supplementary file 5).

Theme 1 – identifying and diagnosing skin conditions.

The challenge of identifying and diagnosing skin conditions was a common focus and frequently described as a source of stress:

> *"Skins a nightmare"*
> *(Workshop 2, Pharmacist 1)*

> *"One of the worst things I can hear in a pharmacy is when a patient says, "can I speak to the pharmacist? Can they tell me what this rash is my child has got?""*
> *(Workshop 2, Pharmacist 2)*

Difficulties identifying *reliable* resources were recognised. Google images, the National Health Service (NHS) website, Clinical Knowledge Summaries website (CKS) or National Institute for Health and Care Excellent (NICE) guidelines were all discussed, but using *standard* photographs was not always found to be helpful. That some resources (e.g., CKS and NICE) do not contain images further impacts upon their utility.

Pharmacists explained that this is particularly an issue with skin conditions as members of the public commonly show them affected skin, rather than verbally describing symptoms as they do with other conditions.

The development of pharmacy specific resources (e.g., online toolkits, in person training etc.) was recognised as a potentially important area for future research and action.

> Possible Research Question - Would dedicated resources improve the identification of skin conditions in community pharmacy?

For peer review only

Theme 2 - Identifying and diagnosing skin conditions in skin of colour
Discussion of skin of colour proceeded almost as an extension of theme 1. With a few exceptions, most participants described identifying skin conditions in skin of colour as more difficult:

> *"I know fungal infection definitely look[s] different on like very dark skin, but I don't know whether my diagnosis would be right, so I'm just always doubting myself. Yeah, so one other thing is, um, discoid eczema [and] ringworm they look very similar on like dark skin or fair skin. So that comes up all the time. I get asked whether it's eczema ringworm all the time. And I don't know the difference."*
> *(Workshop 1, Pharmacist 3)*

Knowing when a condition was getting worse was also considered more challenging in skin of colour. Again, an absence of *reliable, high-quality, evidence-based* resources was considered a barrier to effectively responding to queries and questions.

> Possible Research Question - Would dedicated resources improve the identification of skin conditions in skin of colour in community pharmacy?

Theme 3 - Knowing when to refer skin conditions.
Members of the public described using community pharmacy as a form of triage, seeking advice about whether a condition was "*serious enough*" to consult other healthcare professionals. For some a pharmacist's advice had been an important factor in being confident enough to seek a doctor's appointment.

This was a role that pharmacists recognised but were not always comfortable with; they had specific concerns about *delaying diagnosis* of serious conditions, *missing infectious diseases* or a fear of *making a condition worse* by giving the wrong advice:

> *"Some condition can wait for next day or next week, but some condition need to be managed quite soon. Like same day referral. So I think my challenge was whether to refer […] because weekend 111 is so busy they take hours for them to the doctor they call them back. So sometimes they like go to walk in centre or wait. So I think it's either they can wait till next day or next few days or. With that same day, it's my challenge."*
> *(Workshop 1, Pharmacist 3)*

These concerns were considered more critical if advice was being sought about a child.

Pharmacists also identified that it was not always easy to contact other health professionals and that it is difficult to know when and how to refer patients. The potential for better connected services in the management of skin conditions was also recognised in one of our interviews (with a GP), although it was also recognised that resources might be a barrier to this.

> Possible Research Question - Would dedicated resources support community pharmacists to effectively refer skin conditions that require urgent or more specialist attention?

Theme 4 - Disease specific concerns
Workshop discussion did not confirm such a strong focus upon specific skin conditions as the survey data, but rather pointed to general challenges of managing skin conditions in community pharmacy. An absence of feedback, and of knowing the outcome of advice was commonly described.

For peer review only

"So even though I've kind of recommended this steroid, or I've recommended this emollient, I don't know whether it worked or not because they just don't come back. Even if it's a regular customer."
(Workshop 2, Pharmacist 4)

This makes it harder for a pharmacist to feel fully confident in the advice that they are providing. Similarly, pharmacists rarely gained feedback when referring an individual to a GP, although subsequently seeing the GP's prescriptions might offer some informal insight.

The potential for pharmacists to be more involved in managing skin disease was commonly recognised in both the pharmacist workshops as well as healthcare professional interviews. A few suggested that this might be in diagnosing and suggesting initial treatments, others focused upon counselling on long-term medication use:

"Perhaps a bigger and perhaps more important role for pharmacists is actually in supporting patients with long term chronic skin conditions. Because there are loads of people out there with eczema, acne, psoriasis and so on who don't really get the best out of their treatment and end up going into secondary care because they are very poorly managed … I think this is a golden opportunity for community pharmacists to get more involved is actually in supporting those patients."
(Workshop 1, Pharmacist 5)

Possible research question - How can community pharmacists work most effectively with other healthcare professionals in the identification and management of skin disease?

Theme 5 - Product specific concerns
During the workshops pharmacists communicated that they were confident about their knowledge of products used to manage skin conditions. Members of the public reinforced the importance of this by communicating that they expected pharmacists to understand the products that they were providing:

"It seems to me that a pharmacist should be an expert on the products. And if they're not already an expert on the products then one questions what they're doing as a pharmacist. Sorry"
(Workshop 5, Patient 1)

During the pharmacist workshops some frustration was communicated about not being allowed to provide certain products over the counter, products that customers would subsequently receive on prescription from their GPs:

"I do recognize some or quite a few skin conditions, I would like to give them something that is prescription only, but I can't. So then I have to send them off to the GP, so I would personally like some sort of PGD [Patient Group Direction] or guidelines to be able to prescribe [erm], to do a course be accredited and to be able to prescribe that maybe not to have to go through the whole performance of becoming an independent prescriber, because I don't have the time or the facility to do that."
(Workshop 4, Pharmacist 6)

Some members of the public were equally frustrated by this, confused about why they could order medications from the internet but not access them directly via community pharmacies. Topical corticosteroids (TCS) were often discussed in this way. Members of

For peer review only

the public described how they had lied about how they were going to use TCS to ensure that it was provided:

> *"the only time it was mentioned [topical corticosteroids] was when they refused to sell me it, you know, and that that that sounds stupid. It was, you know, when I've got it on prescription, there's never been any query or any conversation about it. It's just been given in a bag. [And] But when I needed to actually purchase something over the counter. And that's when the interrogation started."*
> *(Workshop 5, Patient 2)*

The centrality of product knowledge suggests that it could be an important area for research and resource development. The potential to extend what pharmacists can do may be important in this.

> Possible research question - Could a wider range of products and treatments for skin conditions be made available via community pharmacy?

> Possible research questions - Would dedicated resources support community pharmacists in the management of skin conditions?

Theme 6 – Other topics

Discussion of the themes identified in the online survey often prompted a broader discussion of community pharmacy and skin conditions. This led to the identification of additional areas where research might be warranted.

In the workshops pharmacists reinforced the notion that they see a broad range of skin conditions daily, and that the number of customers seeking advice about skin conditions is increasing.

> *"I think the numbers of skin referrals with the CPCS* [Community Pharmacy Consultation Service] *is going to go up into Community pharmacy because it's one thing that the GPs can triage without seeing."*
> *(Workshop 4, Pharmacist 7)*

Pharmacists, however, were less confident in making an assessment about how demand is growing and evolving, e.g. which clinical conditions, what types of enquiry, specific demographic groups, adults/children, etc. Discussion in the workshops, as well as some one-to-one interviews, recognised that understanding trends in demand could be an important precursor to any substantive change in how community pharmacy works or engages with skin conditions.

> Possible research question - In what ways are community pharmacists currently involved in the identification and management of skin conditions?

Some of these discussions (especially in the pharmacist workshop) exposed localised variation in what is available and what pharmacists are allowed to do.

> *"We're lucky like, in England, as you have heard you saying that you guys have to charge your folk for it. In the pharmacy first, one thing we've got, is we can give it out free of charge and they can come back six times and get six different bottles and it doesn't cost them anything"*
> *(Workshop 2, Pharmacist 1)*

Discussion demonstrated that variations are manifest in: (i) the skin conditions that pharmacists are paid to treat via minor ailments schemes; (ii) the medications which can be provided via the use of patient group directions (PGDs); and (iii) whether patients had to pay for their treatment. As with understanding trends in demand it was

For peer review only

considered pertinent to develop a better understanding of the success of current skin focused initiatives and ways of working as a precursor to any further development.

Possible research question - What are the known benefits of community pharmacy involvement in the identification and management of skin conditions?

The public workshops offered a slightly different perspective on this topic, focusing upon establishing that pharmacists are appropriately qualified and competent to deal with skin conditions.

"It just feels that pharmacists know a lot about their medicines and of course they know the pros and cons of uses, but whether they have got the expertise in recognizing a particular type of rash or a particular type of mark on the skin […] I wouldn't know if they had that expertise."
(Workshop 7, Patient 3)

Similar concerns were expressed in the healthcare professional interviews, with one GP indicating that they saw "a lot of inappropriate" referrals from pharmacists.

Once again, the benefit of establishing the state of current provision for skin conditions in community pharmacy was recognised as an appropriate focus.

Possible research question - How competent are community pharmacists in the identification and management of skin conditions?

Discussion of direct experience of accessing community pharmacy exposed that participants in the public workshops were often polarised, between those that regularly used their pharmacist and those that were not aware that pharmacists offered this type of service.

"I never knew that they could advise you on skin you know problems, I never knew that. Because I thought they dealt dealt with drugs only and they all seemed very busy. So, I'd like to know how the pharmacist can help with skin conditions as well?"
(Workshop 6, Patient 4)

A concern about a lack of awareness about pharmacy services was echoed in the healthcare professional interviews, where GPs described difficulties convincing patients that minor ailments schemes are appropriate to use. Identifying barriers and encouraging the use of community pharmacy might be important research that underpins the effectiveness of any specific initiative:

Possible research question - What could be done to raise awareness of the skills that community pharmacists have with regards to the identification and management of skin conditions?

A list of all ten research questions is provided in Table 3 (see online Supplementary File 6 for research questions with explanatory notes).

For peer review only

Table 3 The ten research questions from the community pharmacy and dermatology priority setting partnership (in no particular order, prioritised questions in bold)

1. **Would dedicated resources improve the identification of skin conditions in community pharmacy?**
2. Would dedicated resources improve the identification of skin conditions in skin of colour in community pharmacy?
3. Would dedicated resources support community pharmacists to effectively refer skin conditions that require urgent or more specialist attention?
4. **How can community pharmacists work most effectively with other healthcare professionals in the identification and management of skin disease?**
5. Could a wider range of products and treatments for skin conditions be made available via community pharmacy?
6. Would dedicated resources support community pharmacists in the management of skin conditions?
7. In what ways are community pharmacists currently involved in the identification and management of skin conditions?
8. What are the known benefits of community pharmacy involvement in the identification and management of skin conditions?
9. **How competent are community pharmacists in the identification and management of skin conditions?**
10. What could be done to raise awareness of the skills that community pharmacists have with regards to the identification and management of skin conditions?

For peer review only

Stage 3 - prioritisation workshops

To accommodate all those interested in participating, the prioritisation workshop was split into two parts which ran consecutively. The first prioritisation workshops included one dermatology nurse specialist and 10 patients. The second prioritisation workshop included three pharmacists, two members of pharmacy staff and 13 patients (Table 2). Scores at the end of the first workshop were carried forward as the starting point for the second.

At the conclusion of the second workshop the following questions were voted to be the most important (3 participants did not vote):

1. How can community pharmacists work most effectively with other healthcare professionals in the identification and management of skin disease? 11/15 participants
2. How competent are community pharmacists in the identification and management of skin conditions? 9/15 participants
3. Would dedicated resources improve the identification of skin conditions in community pharmacy? 6/15

DISCUSSION

Summary

Through consultation with a range of stakeholders including pharmacists, pharmacy staff, GPs, dermatology nurses and members of the public we have developed a set of research questions to support dermatology provision in community pharmacy. In a final prioritisation exercise we have established three topics as a starting point for a dermatology/community pharmacy research agenda.

Discussion in our workshops reinforced existing assessment that dermatology is a significant part of the workload faced by community pharmacists [12,14,28]. It also reinforced the expectation that this demand is likely to grow in future. Concerns about limited training and knowledge about skin conditions [1] were evident in comments about lacking confidence in dealing with skin queries. A perceived lack of resources, training and post-consultation feedback were recognised as factors in this. Developing resources for pharmacists/pharmacy staff might be an important area where research can benefit pharmacy practice – resources in this context might be training programmes, information resources for staff, information resources for the public, and they could be delivered in print, in person or online.

Research uncertainties identified here might suggest the value of repeating and/or expanding prior research which has considered pharmacists' ability to identify skin conditions [22,23].

Our findings also reinforce more general issues of public awareness about the role of community pharmacy [29,30], specifically recognising this to be an issue with regards to skin conditions. Previous work has identified that individuals felt that only doctors are "qualified/trustworthy" to manage skin complaints [11], a view also expressed by medicine counter assistants[1]. This is a particularly important challenge to negotiate given that all stakeholders who took part in the exercise recognised that the demand in primary care exceeds what GPs are able to manage. However, as there is currently a shortfall in the number of pharmacists and pharmacy staff, it is possible that, under the current workforce model pharmacists may also struggle to meet this demand[31].

Further information about the questions

We have developed a broad range of questions reflecting a broad range of concerns described in the workshops.

We have framed questions loosely and in general terms to allow interpretation and wide scope for impact. For example, the final question, "*How can community pharmacists work most effectively with other healthcare professionals in the identification and management of skin disease?*" could be approached in terms of consideration of the appropriate prescribing of "over the counter" medicines or in terms of the provision of information for regarding the use of long-term prescription medications.

In this, however, we might suggest that there is a form of natural hierarchy with questions focused upon understanding current provision a necessary precursor to research which develops new ways of working. For example, answering, "*in what ways are community pharmacists currently involved in the identification and management of skin conditions?*", would allow future work to be appropriately directed to the most common or most difficult to manage skin conditions.

We would encourage researchers to develop the focus of research in meaningful ways, mindful of the changing landscape within community pharmacy with initiatives such as the independent prescribing schemes in England[3]. We would also encourage

For peer review only

researchers to consider how resources directed towards community pharmacists can deliver consistent messages to other health care professionals.

The term diagnosis in relation to pharmacist-led activity may not be as widely understand across the globe. However, our survey found identifying and diagnosing skin conditions a key area for further research. This research could include the role of the pharmacist is diagnosing conditions compared to simply identifying them.

Strengths and limitations

This has been a broad reaching exercise which has included 111 community pharmacists in an online survey as well as 57 workshop and interview participants. Workshops sought input from both healthcare professionals as well as members of the public – numbers were approximately even in the exploratory workshops (27 healthcare professionals / 30 members of the public).

Whilst we are confident that a broad range of perspectives were considered in the identification of research topics the final prioritisation stage was heavily weighted towards the input of members of the public. At this stage of our process the goal of equal numbers was not achieved, with only five pharmacists/pharmacy staff participating in the prioritisation stage (despite twelve signing-up). Further work in this area could explore different methods of engaging with GPs, other stakeholders and pharmacists to improve recruitment. We should consequently recognise that the final prioritisation more accurately reflects the public view of what research would be most beneficial, rather than a multi-perspective assessment of this.

CONCLUSION

Pharmacists are regularly consulted regarding skin conditions and do not always feel confident in the identification of skin disease. Using information from focus groups with pharmacists, members of the public and other stakeholders we developed 10 research questions that can be used to direct future research to address these challenges.

For peer review only

ETHICAL APPROVAL STATEMENT

Ethical approval for the survey was obtained from Aston University Research Ethics committee. The Faculty of Medicine & Health Sciences Research Ethics Committee, at the University of Nottingham, confirmed that Ethical approval was not required for the workshops as the research was an engagement activity to inform future research priorities. However, at the start of each workshop we explained the purpose of the study and asked whether participants were happy to be recorded.

FUNDING STATEMENT

This project is funded by the National Institute for Health and Care Research (NIHR) School for Primary Care Research (project reference 522).

The views expressed are those of the author(s) and not necessarily those of the NIHR or the Department of Health and Social Care.

CONFLICTS OF INTEREST

JH, IM, CA, KST, PL, ZS have declared they have no conflict of interest.

MB has received personal fees from Delphi Healthcare outside the submitted work, has grants or contracts with Health Education England (Grant for development of experimental learning activity)and Walgreen Boots Alliance (50% funding for PhD studentship). MB has received consulting fees from Clinical Care Quality Solutions (payment split between institution and author). MB has received payment from the Ministry of Health Singapore, Human Manpower Development programme (honoraria to speak including travel and accommodation). MB is a Project advisor/Chair of experiential learning during the MPharm research project. NHS Scotland (payment to institution and author). MB is Vice Chair Pharmacy Law and Ethics Association (no fee received).

MS has the following Grants or contracts:

RAPID and Efficient Eczema Trials (RAPID programme) – lead applicants Thomas and Roberts. NIHR PGfAR NIHR203279 funding to University of Southampton for 5% of my time.

Trial of IGe tests for Eczema Relief (TIGER): randomised controlled trial of test-guided dietary advice for children with eczema, with internal pilot and nested economic and process evaluations – lead applicant Ridd. NIHR HTA NIHR133464 funding to University of Southampton for 10% of my time

Pragmatic, primary care, multi-centre, randomised superiority trial of four emollients in children with eczema, with internal pilot and nested qualitative study (Best Emollients for Eczema - BEE) – lead applicant Ridd. NIHR HTA 15/130/07 completed Aug 2020 funding to University of Southampton for 10% of my time.

MS is a Funding panel member NIHR Programme Grants for Applied Research 2018 to present day and also Academic PPIE lead and Board Member NIHR School for Primary Care Research 2022 to present day.

MR has received Various NIHR grants for studies of skin conditions/food allergy. MR is On TSC/DMC for ERICA, PRINCIPLE and ALPHA trials and is Co-Chair SAPC & NIHR SPCR skin/allergy research groups.

For peer review only

CONTRIBUTIONS

ZS and IM conceptualised, designed and conducted the survey, ZS and JH analysed the survey data, JH and PL led the workshops and interviews, analysis of the interview and workshop data and drafted the manuscript, comments were made on the draft by ZS, IM, MS, MR, MB, KST and CA. All authors commented on and approved the final draft.

DATA SHARING STATEMENT

Data is available from the authors on request.

For peer review only

598

For peer review only

REFERENCES

1. Tucker R, Stewart D. An exploratory study of the views of community pharmacy staff on the management of patients with undiagnosed skin problems. *Int J Pharm Pract* 2015;23(6):390-98. doi: 10.1111/ijpp.12179
2. Tucker R, Stewart D. Why people seek advice from community pharmacies about skin problems. *Int J Pharm Pract* 2015;23(2):150-53. doi: 10.1111/ijpp.12126
3. Medicines and Pharmacy Directorate - Department of Health and Social Care. The Community Pharmacy Contractual Framework for 2019/20 to 2023/24: supporting delivery for the NHS Long Term Plan. London2019 [Available from: https://assets.publishing.service.gov.uk/government/uploads/system/uploads/attachment_data/file/819601/cpcf-2019-to-2024.pdf accessed April 2022.
4. Delgutte A, Santos D. Community pharmacy has been an indispensable element of the covid-19 response BMJ online; [Available from: https://blogs.bmj.com/bmj/2021/02/21/community-pharmacy-has-been-an-indispensable-element-of-the-covid-19-response/#:~:text=Community%20pharmacy%20has%20been%20an%20indispensable%20element%20of%20the%20covid%2D19%20response,-February%2021%2C%202021&text=Community%20pharmacists%20are%20trained%20to,rest%20of%20the%20healthcare%20system accessed April 2022.
5. NHS England. NHS Community Pharmacist Consultation Service (CPCS) – integrating pharmacy into urgent care [Available from: https://www.england.nhs.uk/primary-care/pharmacy/pharmacy-integration-fund/community-pharmacist-consultation-service/ accessed October 2022.
6. The King's Fund. How can dermatology services meet current and future patient needs while ensuring that quality of care is not compromised and that access is equitable across the UK? . London, 2014.
7. Le Roux E, Edwards PJ, Sanderson E, et al. The content and conduct of GP consultations for dermatology problems: a cross-sectional study. *British Journal of General Practice* 2020;70(699):e723-e30. doi: 10.3399/bjgp20X712577
8. Schofield J, Grindlay D, Williams H. Skin conditions in the UK : a health care needs assessment: Centre of Evidence Based Dermatology Nottingham, 2009.
9. Eedy D. The crisis in dermatology. *BMJ* 2015;350:h2765. doi: 10.1136/bmj.h2765
10. Fielding S, Porteous T, Ferguson J, et al. Estimating the burden of minor ailment consultations in general practices and emergency departments through retrospective review of routine data in North East Scotland. *Fam Pract* 2015;32(2):165-72. doi: 10.1093/fampra/cmv003
11. Hammond T, Clatworthy J, Horne R. Patients' use of GPs and community pharmacists in minor illness: a cross-sectional questionnaire-based study. *Fam Pract* 2004;21(2):146-9. doi: 10.1093/fampra/cmh207
12. Tucker R. Community pharmacists' perceptions of the skin conditions they encounter and how they view their role in dermatological care. *Int J Pharm Pract* 2012;20(5):344-6. doi: 10.1111/j.2042-7174.2012.00212.x
13. Tucker R. An exploratory study of uk community pharmacist's perceptions of the skin conditions they encounter: prevalence, reasons for referral and over-the-counter dermatological wish list. *Selfcare* 2013;4(1):3-17.
14. Dodds L, Katusiime B, Shamim A, et al. An investigation into the number and nature of the urgent care consultations managed and referred by community pharmacists in South-East England. *Prim Health Care Res Dev* 2020;21:e5. doi: 10.1017/S1463423620000031
15. NHS Business Services Authority. NHS Community Pharmacist Consultation Service (Minor Illness and Urgent Repeat Medicines Supply pathways) 2020 [Available from: https://www.nhsbsa.nhs.uk/pharmacies-gp-practices-and-appliance-contractors/dispensing-contractors-information/nhs-community-pharmacist-consultation-service-minor-illness-and-urgent-repeat-medicines-supply accessed 9th June 2023.

16. Wagner A, Noyce PR, Ashcroft DM. Changing patient consultation patterns in primary care: an investigation of uptake of the Minor Ailments Service in Scotland. *Health Policy* 2011;99(1):44-51. doi: https://doi.org/10.1016/j.healthpol.2010.07.002
17. Community Pharmacy England. Service case study: the Midlands Community Pharmacy Extended Care Service 2022 [Available from: https://cpe.org.uk/lpcs-and-local/locally-commissioned-services/services-case-studies-hub/service-case-study-the-midlands-community-pharmacy-extended-care-service/ accessed 9th June 2023.
18. The Pharmaceutical Journal. Pharmacy First will be introduced in England in 2023, says government. 2023;310(7973) doi: 10.1211/PJ.2023.1.184384
19. The Pharmaceutical Journal. When will England get a 'Pharmacy First' service? 2023;310(7969) doi: DOI:10.1211/PJ.2022.1.126642
20. NHS Scotland. NHS Pharmacy First Scotland Service 2020 [Available from: https://www.communitypharmacy.scot.nhs.uk/nhs-boards/nhs-lothian/pharmacy-first/ accessed 9th June 2023.
21. Rubsam ML, Esch M, Baum E, et al. Diagnosing skin disease in primary care: a qualitative study of GPs' approaches. *Fam Pract* 2015;32(5):591-5. doi: 10.1093/fampra/cmv056
22. Tucker R, K. M, V. P, et al. An exploratory study of community pharmacist diagnosis and management of dermatitis and acne. *Selfcare* 2017;8(2):1-10.
23. Tucker R, Patel M, Layton AL, et al. An exploratory study demonstrating the diagnostic ability of healthcare professionals in primary care using online case studies for common skin conditions. *Int J Pharm Pract* 2014;22(2):119-24. doi: 10.1111/ijpp.12045
24. Nygaard A, Halvorsrud L, Linnerud S, et al. The James Lind Alliance process approach: scoping review. *BMJ Open* 2019;9(8):e027473. doi: 10.1136/bmjopen-2018-027473
25. Hsieh HF, Shannon SE. Three approaches to qualitative content analysis. *Qual Health Res* 2005;15(9):1277-88. doi: 10.1177/1049732305276687
26. Gale NK, Heath G, Cameron E, et al. Using the framework method for the analysis of qualitative data in multi-disciplinary health research. *BMC Med Res Methodol* 2013;13:117. doi: 10.1186/1471-2288-13-117
27. Delbecq AL, Van de Ven AH. A Group Process Model for Problem Identification and Program Planning. *The Journal of Applied Behavioral Science* 1971;7(4):466-92. doi: 10.1177/002188637100700404
28. Hafejee A, Coulson IH. Community pharmacists' role in managing common skin problems. *Br J Dermatol* 2006;155(6):1297. doi: 10.1111/j.1365-2133.2006.07545.x
29. Latif A, Waring J, Watmough D, et al. Examination of England's New Medicine Service (NMS) of complex health care interventions in community pharmacy. *Res Social Adm Pharm* 2016;12(6):966-89. doi: 10.1016/j.sapharm.2015.12.007
30. Latif A, Waring J, Watmough D, et al. 'I expected just to walk in, get my tablets and then walk out': on framing new community pharmacy services in the English healthcare system. *Sociol Health Illn* 2018;40(6):1019-36. doi: 10.1111/1467-9566.12739
31. Company Chemists Association. Review of the Community Pharmacist Workforce in England 2022 [Available from: https://thecca.org.uk/cca-publishes-review-of-the-community-pharmacist-workforce-in-england/ accessed December 2022.

For peer review only

Derm/Comm Pharm PSP Workshop 1 – Community Pharmacists

7pm WELCOME & INTRODUCTIONS FROM US.

- Thank-you for coming.
- Introductions from PL / JH.
- Purpose of the project.

The purpose of this project is to identify uncertainties that community pharmacists experience when they are faced with someone seeking help about a skin condition.

Our goal is to identify a small number of research projects which could be undertaken to support community pharmacists - these could be trialling new ways of working, developing information resources, understanding the challenges of seeing skin conditions in community pharmacy, testing treatments, etc.

- Purpose of this evening.

From the first stage of the project, an online survey of community pharmacists, we have identified several areas that we would like to understand further, outlined below.

- Rules of engagement.
- Recording start.

7.10pm AROUND PARTICIPANTS – INTRODUCTIONS.

- name, where from, pharmacist role/background.
- 1 thing about skin….

Is there one thing that you would like to know that would help you when dealing with a skin condition / skin condition query?

7.25pm YOUR SCORING OF THE MATERIALS SENT.

7.30pm TOPIC 1.

7.45pm TOPIC 2.

8.00pm SHORT BREAK.

8.10pm OTHER TOPICS AND QUESTIONS.

8.40pm RANKING/SCORING.

8.50pm. CLOSING COMMENTS AND THANKS.

- vouchers.
- final workshop date and possibilities.

For peer review only

Identifying and diagnosing skin disorders.
What's the biggest challenge in this // What would help? What do you need?

For example:
The need for resources to support skin condition identification.
Specific skin conditions that you find difficult to identify (moles, insect bites, general rashes).

Additional prompts:
[is it about skin conditions? Is it about facilities/resources? Is it about knowledge/resources]

What's the biggest challenge in this – lack of knowledge, anxious public, number of conditions.
Adults / children?
Mild and very early presentation?
Time? And facilities?
Existing resources – adequacy? Appropriateness?
Training.
What do you currently do – examples of good practice.

Knowing when to refer someone to a GP.
What's the biggest challenge in this // What would help? What do you need?

For example:
Knowing which conditions should be referred to a GP (or when the severity of a condition requires referral).
Understanding how to refer / the need for more formal mechanism of referral.
Knowing when it is ok to offer advice/product and manage locally.

Additional prompts:
[is it about skin conditions? Is it about facilities/resources? Is it about knowledge/resources]

Are there specific situations/conditions where this is a particular problem?
What would discourage you from referring a patient?
Would having a referral pathway be useful similar to those between GPs and secondary care?
Is the reason for referral due to not knowing the diagnosis or not being able to treat the condition once diagnosed?

Skin of Colour.
What's the biggest challenge in this // What would help? What do you need?

For example:
Understanding the different presentation of skin conditions in skin of colour.
Knowing skin conditions which are distinct / more prevalent in skin of colour.

Additional prompts:
[is it about skin conditions? Is it about facilities/resources? Is it about knowledge/resources]

Did you receive any training specifically in diagnosing/treating conditions in skin of colour?
How have you learnt about treating conditions in patients with skin of colour?
Are there any conditions that you see more frequently in patients with skin of colour?

Disease specific concerns.
What's the biggest challenge in this // What would help? What do you need?

For example:
Recognising specific conditions which can / cannot be managed in community pharmacy.
Understanding how to support specific, chronic skin conditions.
The potential for dedicated roles in supporting specific conditions – trained staff, dedicated services.
Supporting members of the public with eczema self-management.

Additional prompts:
[is it about skin conditions? Is it about facilities/resources? Is it about knowledge/resources]

What long-term conditions do you see the most frequently?
What conditions do you see patients struggle to deal with?

For peer review only

What conditions do think a pharmacist could help to manage?
What role do you currently have in managing long-term conditions?
What role would you like to have with managing conditions both acute and long-term?

Product specific concerns.

For example:
Understanding concerns about the use of topical corticosteroids.
Understanding how to navigate the range of products available for skin conditions.
Understanding the benefit of products for different conditions and different severities. Knowing conditions for which you have no treatment.

Additional prompts:
[is it about skin conditions? Is it about facilities/resources? Is it about knowledge/resources]

What other medications do you think should be available OTC? Could be tested as OTC?
What are the common problems you have when selling TCS OTC? How do you think these problems should be managed?
What conditions are the most difficult to treat with the products that you have available?
How do you think

For peer review only

Public Pharmacy Dermatology Priority Setting Workshop

7pm WELCOME & INTRODUCTIONS FROM US.

- Thank-you for coming.
- Introductions from PL / JH.
- Purpose of the project.

The purpose of this project is to identify uncertainties that community pharmacists experience when they are faced with someone seeking help about a skin condition.

Our goal is to identify a small number of research projects which could be undertaken to support community pharmacists - these could be trialling new ways of working, developing information resources, understanding the challenges of seeing skin conditions in community pharmacy, testing treatments, etc.

- Purpose of this evening.

This evening we want to talk about your experiences of using a community pharmacy and discuss what roles you think pharmacists can play in the management of skin conditions. We want to know what types of projects you think need to be done that could help pharmacies to offer the best possible care to their patients.

- Rules of engagement.
- Split into two groups.
- Recording start.

7.10pm AROUND PARTICIPANTS – INTRODUCTIONS.

- name, where from, do you have any skin conditions/ experience of having skin conditions,

7.20pm

- Have you ever consulted a pharmacist/member of pharmacy staff regarding a skin condition? What were your experiences of using community pharmacy to treat skin conditions? Would you be as happy to see a pharmacist about a skin condition as you would about a cold? Do you have a long-term community pharmacist? Why do you go to that pharmacist?
  - Positive
  - Negative

- What would encourage you to use a community pharmacy for a skin condition?
  - Access
  - lack of GP appointments?
  - Familiarity (do you know your local pharmacist?)
  - See skin condition as a minor condition and not worthwhile bothering a GP (seeking reassurance from pharmacist that they should see a GP)

- What would discourage you from using a community pharmacy
  - Privacy (how would you feel about going into the consultation room?)
  - Having to speak to counter staff (would you feel comfortable asking to speak to a pharmacist)
  - Worried about lack of knowledge (would you feel any better knowing that the pharmacist had accessed specialist training in treating skin conditions?)

- When you have been to a community pharmacy which members of staff have you spoken to? How often do they bring the pharmacist into the conversation? Do you always get to see the pharmacist if you ask? Do you trust what the counter assistant tells you? Would you trust the pharmacist more?

For peer review only

- For those of you who have had skin conditions or have cared for people with skin conditions, at what point in your treatment do you think a pharmacist could have improved the quality of the care you/they received from the NHS as a whole.

  Please think about:
    - The initial diagnosis
    - The first time you were prescribed a medication
    - Each time you picked up a prescription from the pharmacy
    - When you purchased a treatment for a skin condition from the pharmacy
    - If the pharmacist referred you to someone else for treatment

- What role do you think the pharmacist should have? Should they diagnose your skin condition? Should they just treat your skin condition?
- What do you know about pharmacists training?

8pm short break

8.10pm

8.10pm RESEARCH TOPICS AND QUESTIONS.

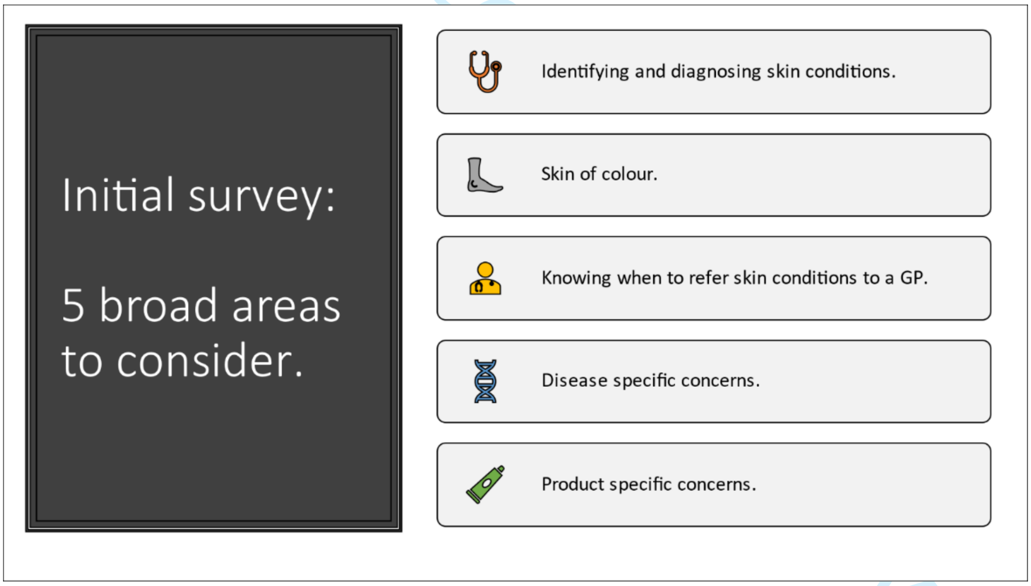

What do you think of this order?
Are you surprised?
Do you think the role of the pharmacist is to diagnose?
Or is their role just to tell you when you need to go to the GP?
Do you trust what a pharmacist says?
Do you think it would be useful if they could diagnose?

Identifying and diagnosing skin disorders.
What's the biggest challenge in this?
What would help? What do you need?

For example:
The need for resources to support skin condition identification.

For peer review only - http://bmjopen.bmj.com/site/about/guidelines.xhtml

Specific skin conditions that you find difficult to identify (moles, insect bites, general rashes).

Current use of photographs / websites / other resources?

What are you most likely to see? What is most likely to be difficult?

Additional prompts:
[is it about skin conditions? Is it about facilities/resources? Is it about knowledge/resources]

What's the biggest challenge in this – lack of knowledge, anxious public, number of conditions.
Adults / children?
Mild and very early presentation?
Time? And facilities?
Existing resources – adequacy? Appropriateness?
Training.
What do you currently do – examples of good practice.

Knowing when to refer someone to a GP.
What's the biggest challenge in this // What would help? What do you need?

For example:
Knowing which conditions should be referred to a GP (or when the severity of a condition requires referral).
Understanding how to refer / the need for more formal mechanism of referral.
Knowing when it is ok to offer advice/product and manage locally.

Knowing how long to wait?

What are you most likely to refer?

Additional prompts:
[is it about skin conditions? Is it about facilities/resources? Is it about knowledge/resources]

Are there specific situations/conditions where this is a particular problem?
What would discourage you from referring a patient?
Would having a referral pathway be useful similar to those between GPs and secondary care?
Is the reason for referral due to not knowing the diagnosis or not being able to treat the condition once diagnosed?

Disease specific concerns.
What's the biggest challenge in this // What would help? What do you need?

What conditions ? what challenges?

What's your role in this? What are you trying to achieve? [help GPs, …]

Do you want to do more?

Integrated services focused upon diseases?

For example:
Recognising specific conditions which can / cannot be managed in community pharmacy.
Understanding how to support specific, chronic skin conditions.
The potential for dedicated roles in supporting specific conditions – trained staff, dedicated services.
Supporting members of the public with eczema self-management.

Additional prompts:
[is it about skin conditions? Is it about facilities/resources? Is it about knowledge/resources]

What long-term conditions do you see the most frequently?
What conditions do you see patients struggle to deal with?
What conditions do think a pharmacist could help to manage?
What role do you currently have in managing long-term conditions?
What role would you like to have with managing conditions both acute and long-term?

Skin of Colour.
What's the biggest challenge in this // What would help? What do you need?

For peer review only

For example:
Understanding the different presentation of skin conditions in skin of colour.
Knowing skin conditions which are distinct / more prevalent in skin of colour.

Additional prompts:
[is it about skin conditions? Is it about facilities/resources? Is it about knowledge/resources]

Did you receive any training specifically in diagnosing/treating conditions in skin of colour?
How have you learnt about treating conditions in patients with skin of colour?
Are there any conditions that you see more frequently in patients with skin of colour?

Product specific concerns.

For example:
Understanding concerns about the use of topical corticosteroids.
Understanding how to navigate the range of products available for skin conditions.
Understanding the benefit of products for different conditions and different severities. Knowing conditions for which you have no treatment.

Additional prompts:
[is it about skin conditions? Is it about facilities/resources? Is it about knowledge/resources]

What other medications do you think should be available OTC? Could be tested as OTC?
What are the common problems you have when selling TCS OTC? How do you think these problems should be managed?
What conditions are the most difficult to treat with the products that you have available?
How do you think

8.40pm RANKING/SCORING.

8.50pm. CLOSING COMMENTS AND THANKS.
- vouchers.
- final workshop date and possibilities.

For peer review only

COREQ checklist

Item number		Notes
1	Which author/s conducted the interview or focus group?	All workshops and interviews were facilitated by both JH and PL.
2	What were the researcher's credentials? E.g. PhD, MD	JH has a Ph.D and MPharm. PL has a Ph.D
3	What was their occupation at the time of the study?	JH and PL work at the Centre of Evidence based Dermatology, School of Medicine, University of Nottingham. JH is a registered pharmacist.
4	Was the researcher male or female?	N/A
5	What experience or training did the researcher have?	PL has extensive expertise in qualitative methods. <br>JH has completed several pieces of qualitative work examining pharmacist led services.
6	Was a relationship established prior to study commencement?	Researchers did not establish a relationship with participants prior to study commencement
7	What did the participants know about the researcher? e.g. personal goals, reasons for doing the research	Participants did not know that JH was a registered pharmacist. They were aware both researchers worked for the Centre of Evidence Dermatology.
8	What characteristics were reported about the inter viewer/facilitator? e.g. Bias, assumptions, reasons and interests in the research topic	We have acknowledged in the write-up that JH has a background as a pharmacist
9	What methodological orientation was stated to underpin the study? e.g. grounded theory, discourse analysis, ethnography, phenomenology, content analysis	This was exploratory, consultative research broadly informed by the James Lind Priority Setting Partnership approach. <br>See methods section, line 200, 206, 213, 216
10	How were participants selected? e.g. purposive, convenience, consecutive, snowball	See methods section line 165
11	How were participants approached? e.g. face-to-face, telephone, mail, email	Line 165,175
12	How many participants were in the study?	Table 1
13	How many people refused to participate or dropped out? Reasons?	Although the first focus groups were attended by 19 pharmacists, the final online consensus workshop was poorly attended by

For peer review only

		pharmacists and members of pharmacy staff. We had initially invited an equal number of pharmacists and pharmacy staff (12) and members of the public (13) but only 5/12 pharmacists and members of pharmacy staff, who were signed up prior to the meeting, attended the meeting.<br><br>Reasons (where provided) for nonattendance included sickness and that they were too busy.
14	Where was the data collected? e.g. home, clinic, workplace	Line 203
15	Was anyone else present besides the participants and researchers?	No
16	What are the important characteristics of the sample? e.g. demographic data, date	We did not collect any demographic data.
17	Were questions, prompts, guides provided by the authors? Was it pilot tested?	Workshops used the findings of earlier stages of the research as their focus.<br><br>Data analysis and progress summaries were considered by the wider author group and PPI representatives prior to the workshops.
18	Were repeat inter views carried out? If yes, how many?	N/A
19	Did the research use audio or visual recording to collect the data?	Line 211
20	Were field notes made during and/or after the interview or focus group?	No fieldnotes were made during or after data collection.
21	What was the duration of the inter views or focus group?	Line 250
22	Was data saturation discussed?	Data saturation was not a concept applied to this research, rather data collection
23	Were transcripts returned to participants for comment and/or correction?	No
24	How many data coders coded the data?	PL and JH coded the data. Other authors reviewed coded data and confirmed appropriateness of interpretations.
25	Did authors provide a description of the coding tree?	Yes Table 2

For peer review only

26	Were themes identified in advance or derived from the data?	Initial themes were derived from the inductive content analysis of the online survey. Themes were refined following the exploratory workshops.
27	What software, if applicable, was used to manage the data?	NVivo ver 12 was used to handle research data.
28	Did participants provide feedback on the findings?	No
29	Were participant quotations presented to illustrate the themes/findings? Was each quotation identified? e.g. participant number	Yes, identified by participant number and profession and by workshop number
30	Was there consistency between the data presented and the findings?	Yes findings were based on an analytic process that was iterative.
31	Were major themes clearly presented in the findings?	Yes in the form of the 10 questions
32	Is there a description of diverse cases or discussion of minor themes?	Yes where applicable in the text

For peer review only

Introduction

The responses from two open questions from the Aston Pharmacy Dermatology Survey have been analysed.

We have:

- Identified the top 20 words from the two open questions; the questions concerned are the "challenge" question and the "research priority" question.
- Created word trees from the most common words identified, then used thematic analysis of the word trees to draw up the long list of questions.

Please see below for an example of the analyses mentioned above.

For peer review only

Challenge word analysis

Challenge question: Please list at least one and up to two of the most challenging aspects of treating or managing patients presenting with skin conditions. For example: "How can community pharmacy best support safe use of topical corticosteroids?" or "How do we know when to refer someone with a mole?"

Number of challenge word responses=348

Top 20 words from the challenge question

Word	Count	Similar words
**refer**	44	refer, referal, referred, referring
**rash**	39	rash, rashes
**products**	26	product, products
**differential**	25	differential, differentials, differentiate, differentiating
**know**	24	know, knowing
**diagnosis**	22	diagnosis
**different**	21	difference, differences, different, differently
**steroid**	19	steroid, steroids
**need**	16	need, needed, needing, needs
**support**	16	support, supporting
**treatment**	16	treatment, treatments
**creams**	15	cream, creams
**infected**	15	infected, infection, infections
**time**	15	time, timely, times
**limited**	14	limitations, limited, limits
**difficult**	14	difficult
**emollients**	14	emollient, emollients
**identify**	14	identify, identifying
**treat**	14	treat, treated, treating
**mole**	14	mole, moles

For peer review only

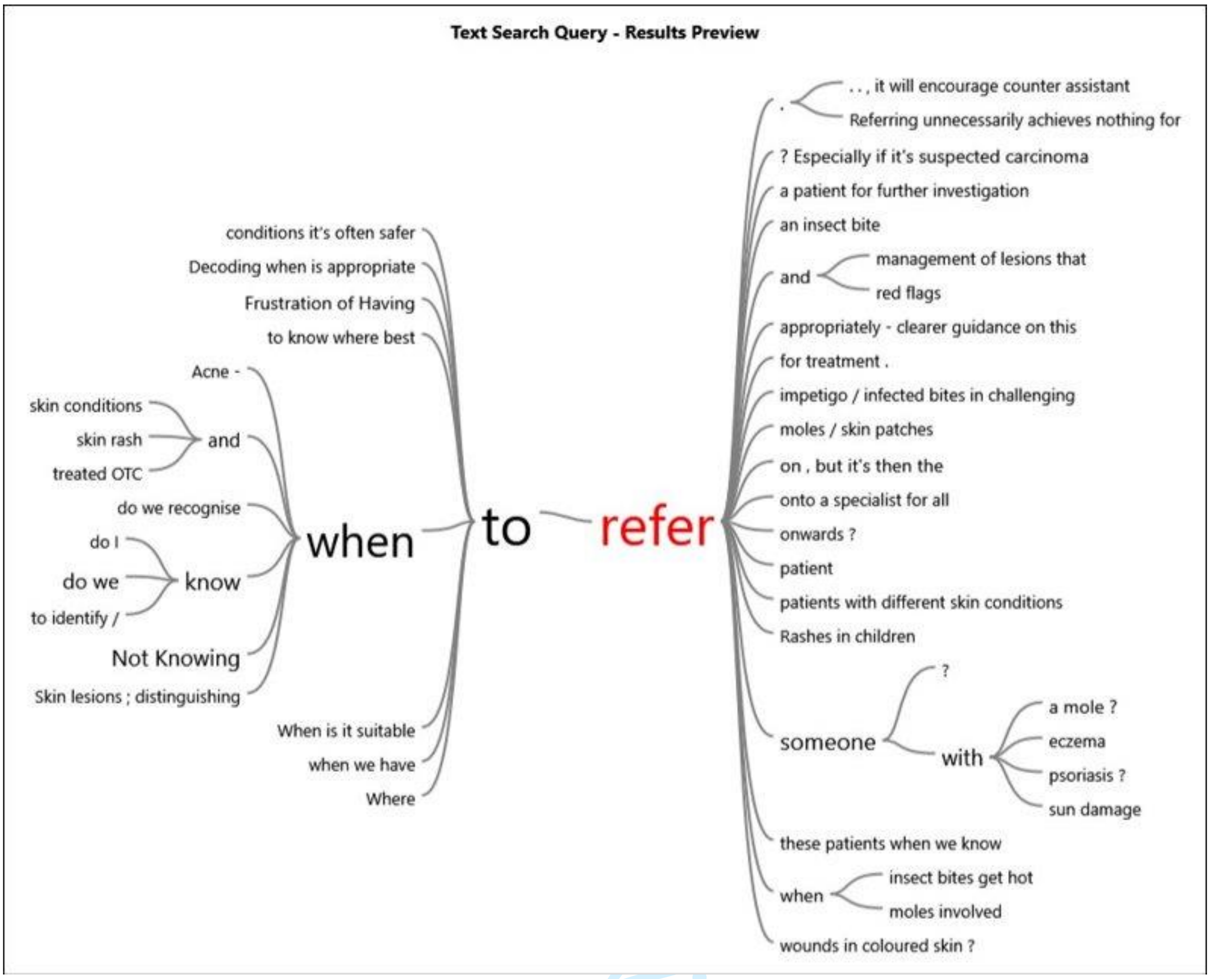

For peer review only - http://bmjopen.bmj.com/site/about/guidelines.xhtml

Research priority analysis question

Please list at least one and up to three of your top research priorities (e.g.what do we need to know more about) in relation to the role of community pharmacy in skincare (consider both advice/counter prescribing and advice on prescription medications).

Number of research priority responses - 522

Top 20 words – research priority questions

Word	Count	Similar words
**treatment**	47	treatment, treatments
**different**	32	difference, different, differing
**products**	31	product, products
**need**	28	need, needed, needing, needs
**creams**	24	cream, creams
**rashes**	22	rash, rashes
**care**	21	care
**steroid**	21	steroid, steroidal, steroids
**pharmacists**	20	pharmacist, pharmacists
**eczema**	19	eczema
**psoriasis**	18	psoriasis
**training**	18	train, trained, training
**prescribing**	17	prescribe, prescribed, prescriber, prescribers, prescribing
**diagnosis**	16	diagnosis
**information**	16	inform, information
**effects**	15	effecting, effective, effectively, effectiveness, effects
**acne**	15	acne
**advice**	15	advice
**infections**	15	infected, infection, infections
**referral**	15	referral

For peer review only

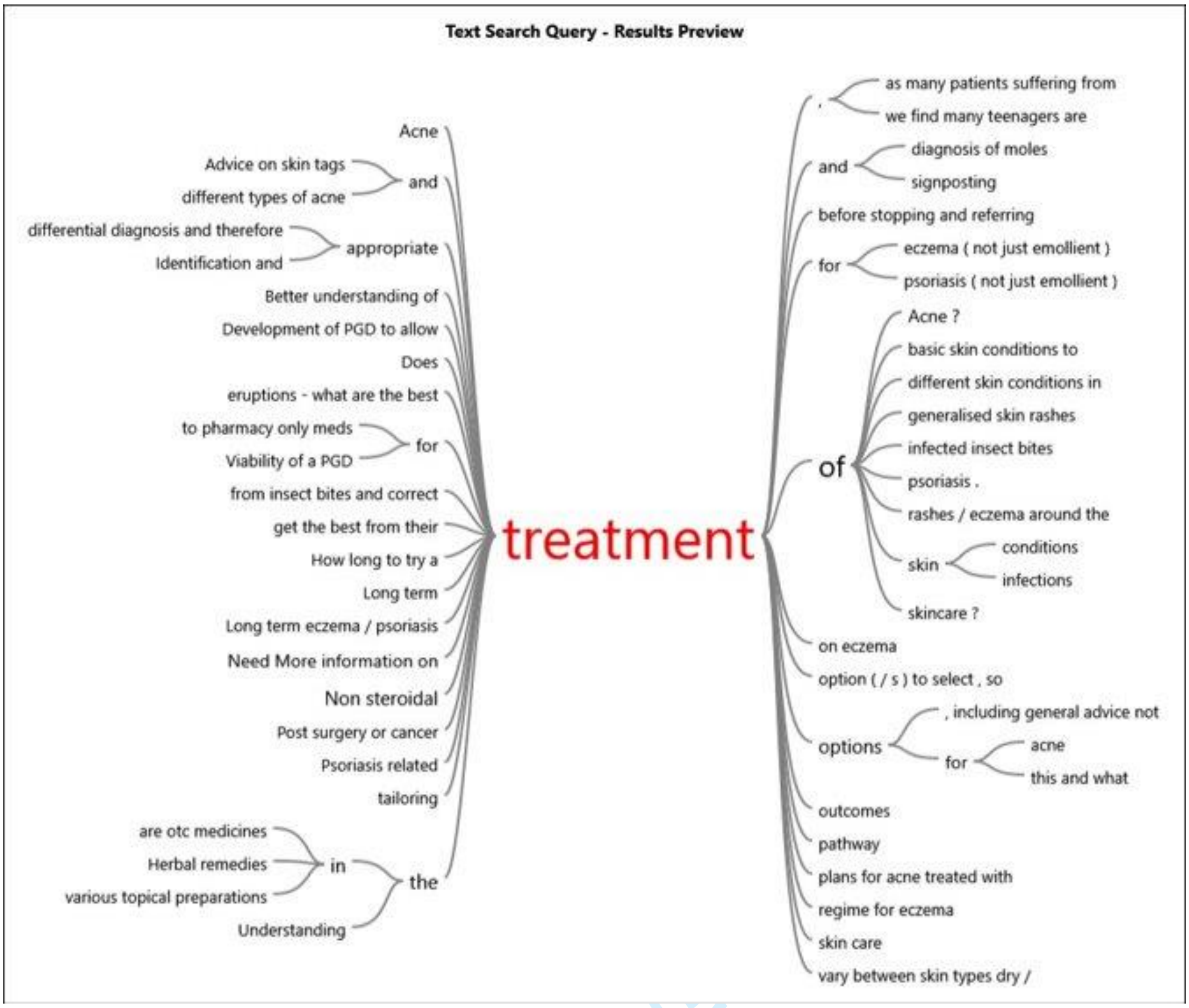
Text Search Query - Results Preview
treatment
Acne
Advice on skin tags
different types of acne
and
differential diagnosis and therefore
Identification and
appropriate
Better understanding of
Development of PGD to allow
Does
eruptions - what are the best
to pharmacy only meds
Viability of a PGD
for
from insect bites and correct
get the best from their
How long to try a
Long term
Long term eczema / psoriasis
Need More information on
Non steroidal
Post surgery or cancer
Psoriasis related
tailoring
are otc medicines
Herbal remedies
various topical preparations
in
Understanding
the
,
as many patients suffering from
we find many teenagers are
and
diagnosis of moles
signposting
before stopping and referring
for
eczema (not just emollient)
psoriasis (not just emollient)
of
Acne ?
basic skin conditions to
different skin conditions in
generalised skin rashes
infected insect bites
psoriasis .
rashes / eczema around the
skin
conditions
infections
skincare ?
on eczema
option (/ s) to select , so
options
, including general advice not
for
acne
this and what
outcomes
pathway
plans for acne treated with
regime for eczema
skin care
vary between skin types dry /

Further quotes to support research questions derived from Themes 1 and 2 – Resources to support Identifying and diagnosing skin conditions and resources to support Identifying and diagnosing skin conditions in skin of colour

Resources to help the Identification of skin conditions within community pharmacies	*"Yeah, if someone says they have dry eyes, you don't recognize patterns. You just accept you have dry eyes" Pharmacist*<br><br>*"I think a lot of dermatology things sometimes we've only seen them in pictures, so not actually in real life. And obviously everyone's different and they don't necessarily present how we've seen them in pictures so you're kind of making a judgment call whether it's close enough to the picture or whether it's something completely different that they're presenting with." Pharmacist*
Resources to help the Identification of skin conditions in skin of colour within community pharmacies	*"OK, I use Google images quite a bit for this because they're like what does it look like in a darker skin tones it and Google Google's great. But you know, like, I I don't know if this is like, you should say, oh, this is looking better. I can't say that in a different colour skin tone." Pharmacist*<br><br>*"Yeah, my worst fear is missing a red flag in like a darker skin tone like cancer. You're more likely to know what it looks like on Caucasian skin, but on dark skin I just worry that I missed the red flag." Pharmacist*

For peer review only

Further quotes to support the research question derived from Theme 3 - Resources to support referral

Resources to support referral of skin conditions	*"I probably wouldn't have gone to A&E had I not sort advice from them first, because I wouldn't have wanted to bother A&E. And so you almost feel like a bit better about going to and when they're really busy about what is just this little thing on your neck, because actually you can say, well, I've just been into boots and I was actually advised to come here and that makes you feel a little bit sort of that you've already gone and sought a bit of help first." Patient*<br><br>*"it would be really nice to think if the pharmacist really wanted to be useful and escalate the problem they knew where to send it to, because GPS also generally don't know that much about [skin] because there's thousands of different skin conditions" Patient*<br><br>*"My fear is we go give out antibiotics for that, or impetigo like give the Fucidin cream out for that and we actually delay treatment for something that maybe is a bit more serious." Pharmacist*<br><br>*"It is sometimes difficult even after all this time it is difficult to determine exactly or to have confidence in what you think a particular rash might be and how infectious it is or is not" Pharmacist*<br><br>*"I think the with children with any sort of, any really well, anytime a child comes to you in a pharmacy, I think you just. You feel. More pressure to make the right decision because. You refer just in case it is something more serious because you've got that doubt in the back of your mind." Pharmacist*<br><br>*"Yes, I struggled to refer things to the GP and just cos our GP is getting massive very quickly. We've got a lot of new builds in our area and so they are quite snowed under and generally I think any appointment time is about four weeks and so it is a struggle to refer and normally we just say if you mentioned that you've been to us because we are opposite, we are, we share a gangway so they are quite nice about it, but it's a struggle to say this is serious enough to be seen tomorrow." Pharmacist*<br><br>*"The idea of suggesting to GP that they sort of take on more of a training role or sort of a feedback system with a community pharmacist might not land very well, but in an ideal world I think it's a great a great idea. I would. I would love to have. So[me] two way dialogue, you know, even if if they if they send pictures through, like our district nurses send pictures of patients all the time of the of a wound on their leg and say do you think this needs antibiotics?" GP*

For peer review only

Further quotes to support the research question derived from Theme 4- Working with other healthcare professionals

**Working with other healthcare professionals**	
Initial management of skin conditions	*"Because what I usually tell patients is in this climate if you're not going to get into the GPS for a few weeks, you may as well try one week of hydrocortisone and this emollient and then you have that information to tell the prescriber. If it's better, brilliant if it's worse, at least you won't be sent away with hydrocortisone and an emollient, and you'll already be one step ahead, but I just don't know whether that's the most useful action, so I think just more of a conversation." Pharmacist*<br><br>*"I think going from Community pharmacy to now working alongside GPs when I do have a a concern about a skin condition or some sort of query, you find that a lot of them actually just use trial and error with different prescription only medications and creams. So they'll just say try this for a week If it doesn't work then come back and a lot of it, even they're not 100% sure." Pharmacist*
Roles in management of long-term skin conditions	*"I I think I think if people have got, […] already got a diagnosis. I think that [upskilling pharmacists to provide counselling] would be really helpful because you know follow up appointments are getting more and more difficult. I think everything's changed so much in the last 20 years because we've now got biologics. So in in secondary care then we're mainly focusing on patients who have had the systemic medications at the topicals, the systemics." Specialist dermatology nurse*<br><br>*"So in all the years that I've been having these meds I've never been offered any kind of advice except for once. When the pharmacist said don't forget to keep these in the fridge because they don't like lots of hot hot temperature." Patient*

For peer review only

Further quotes to support the research questions derived from Theme 5 - Resources to support the management of skin conditions and improving access to medications

Management of skin conditions	*I just always find it difficult with eczema, just the amount of emollients, and it's what emollients to recommend and what circumstance. Pharmacist*
Providing a wider range of treatments	*"I find it difficult to treat over the counter because I feel quite limited in what we're what we're licensed to sell and for what and for use for, like on on the areas of the body" Pharmacy dispenser*<br><br>*"I can't tell you how many referrals I've made for impetigo because there's just nothing to offer" Pharmacist*
Access to topical corticosteroids	*"And as someone mentioned, you have to be quite careful of what you say to them in order to get it [TCS]. " Patients*<br><br>*"I might treat something off license and and pharmacists are very much by the book. But in dermatology, we can't always do things by the book and we have to use things off licence because sometimes there's just nowhere else to go. I mean, you know, I for years, I I had a paediatric eczema clinic. And I just felt so sorry for those parents who, even with the GP, found it difficult to to get the medication that they needed if their children's skin was flaring and they had quite severe flares and they had to wait to come and see us and we only had it clinic once once a week." Specialist dermatology nurse*<br><br>*"[the patient said] I've forgotten my cream and they wanted to buy hydrocortisone cream and they said I've got eczema on my face, and you simply can't sell it. And and and you get into it. So so in many respects you you've got kind of silly restrictions like that which actually impede your ability to practice properly because you think because you effectively look a bit stupid because, well, the doctor gives it to me and you're saying you can't sell it to me and you think what is all that about?" Pharmacist*<br><br>*"I'm not concerned about pharmacists supplying the items inappropriately, but it's the whole over the counter consultation because this my staff know that the face is a no go zone for hydrocortisone. So if somebody was requesting it for the face, that's a hard kind of stop for them to come and grab me to have a conversation with the patient about this. Whereas if that licensing was removed, you may run* the risk *of just missing these patients throughout the whole pharmacy team." Pharmacist*

For peer review only

Further quotes to support Research Question from Theme 6 - Current involvement of pharmacists and the known benefits of the involvement of community pharmacists in the identification and management of skin conditions

Assessment of rising demand	*"And I see such a broad range [..] I just see a bit of everything all the time" Pharmacist*<br><br>*"but I do get a lot of people coming in children with children. All sorts of skin conditions. As you said ringworm and eczema and slap cheek." Pharmacist*<br><br>*"I think it has [consultations for skin disease] increased, but I was still seeing quite a few skin conditions, so people coming in asking before COVID as well asking what it might be and anything [I] could recommend." Pharmacist*<br><br>*"I think it will, I think it will stay [the increased demand], UM I, I think. [It's] the ease of access you know? And because people are not getting appointments with GP" Pharmacist*
Variation in service provisions	*"I think the main ailments aspect needs to be stretched. To cover all areas of the country to make it easier for pharmacies in areas that don't have it. Because some of the things that we can't assist with are ridiculous." Pharmacy dispenser*<br><br>*"[the geographical area] has some PGDs that allow for supply, for example, hydrocortisone on the face under PGD" Pharmacist*<br><br>*"You know, and if I've got the choice of trying to get an appointment to see a GP. And then they'll give me a prescription, and then because I've got a prepayment, I don't have to pay for it or going to a chemist and then charging me £9 for a bottle of Double Base Ointment. The logic says you go to the GP first. You've just saved yourself. You know, a tenner" Patient*<br><br>*"I'm not a massive fan of the CPCS thing yet, cause UM our area doesn't do much minor ailments and they're expecting things for free. And I'm, they're like, no, I can't. I can't give you that you're gonna have to. You're gonna have to pay." Pharmacist*<br><br>*" How well do you trust somebody's clinical judgment against their commercial need?" Patient*

For peer review only

Further quotes to support Research Question 9. and 10. Competency of community pharmacists in managing skin conditions and awareness of the public of the skills of community pharmacists with regards to the identification and management of skin conditions.

Pharmacist competency in the identification and treatment of skin conditions	*"I would guess a certificate on the wall […] go to my optician and he's got all his different specialities on on the wall. So something just with simple as that and I guess they would have to be trained by a consultant dermatologist or at least somebody with many years of experience at spotting all kinds of different conditions." Patient*<br><br>*"I think one I don't know what community pharmacists know, and I think that's part of the problem. We work very closely with community pharmacists in some ways, but we're very, very separate in others, so we phone them up about medication queries and they phone us all the time. But actually knowing what each other's skills are is is quite difficult." GP*<br><br>*"But what I do see is what they've attempted to treat and then comes to us anyway, so I see quite a lot of misdiagnoses from community pharmacies, so a lot of insect bites that look very red and inflamed are sent to us because they think it's infected […] we see lots of sort of cold sore sent to us as query impetigo needing antibiotics, lots of eczema that's just red and inflamed might be infected and gets sent to us and it's not, it just needs eczema treatment so I think. I I see some fairly basic areas of of lacking in knowledge […] but what I don't see are the ones that they have successfully treated and and and sent away so I don't know what sort of ratio we're talking here." GP*
Raising awareness of pharmacy skin services	*"I've had various sort of short term skin conditions and pharmacies have always been my sort of first port of call. It [has] always been good experiences" Patient*<br><br>*"I never knew the pharmacy that they could advise you on skin you know problems, I never knew that. Because I thought they dealt dealt with drugs only and they all seemed very busy. So I'd like to know how the pharmacist can help with skin conditions as well?" Patient*<br><br>*"This minor ailment scheme and for that it needs the patient consent, so we need them to reply to the text message or we need to speak to them on the phone to get their consent and then make the referral, which is a very quick process, but it's all quite clunky and it doesn't always have the patient buy in because they, sometimes incorrectly, value the GP's skills more than the community pharmacists skills, so there needs to be a degree of sort of selling of this and patient education" GP*

For peer review only

N.B. In the notes below, where we refer to community pharmacists, we include any appropriately qualified member of the community pharmacy team.

Question	Explanatory notes
1. What are the known benefits of community pharmacy involvement in the identification and management of skin conditions?	• This could be about establishing what is already known about community pharmacy involvement in the management of skin conditions.<br>• This could be important research which underpins the development of new services or resources.<br>• In our focus groups, participants described several different activities where community pharmacists have helped people with skin conditions. Some examples are these activities are; diagnosing, supporting self-management, recommending the use of products, and participating in dedicated medicines/illness schemes. Knowing which activities have evidence of being beneficial could support future provision.
2. In what ways are community pharmacists currently involved in the identification and management of skin conditions?	• This could be about developing a more detailed understanding about what currently happens in community pharmacy.<br>• It could include assessing levels of demand, mapping different types of activity, and identifying novel ways of working.<br>• In our focus groups it was apparent that services are developing differently across the country and that people were not aware of all that is currently happening in different areas.
3. How competent are community pharmacists in the identification and management of skin conditions?	• This could involve assessing the knowledge and skills of community pharmacists and other members of the pharmacy team.<br>• In our focus groups pharmacists described different levels of confidence and competence in addressing skin conditions. Members of the public were uncertain about how qualified community pharmacists are to manage skin conditions.
4. Would dedicated resources improve the **identification of skin conditions** by community pharmacists?	• This could involve developing and/or testing resources which support community pharmacists in identifying skin conditions. Resources might be online resources, training programmes etc.<br>• In our focus groups pharmacists described an absence of resources that they are in confident in using to identify skin conditions, they said that high quality resources could be useful in identifying skin conditions.
5. Would dedicated resources improve the **identification of skin conditions** in **skin of colour** by community pharmacists?	• This could involve developing and/or testing resources which support community pharmacists in identifying skin conditions in skin of colour.<br>• In our focus groups pharmacists described that identifying skin conditions in skin of colour is an additional challenge due to the different ways that skin conditions can look, and also because most current training/resources present images of white skin.
6. Would dedicated resources support community pharmacists in the **management** of skin conditions?	• This could involve developing and/or testing resources which support community pharmacists in managing skin conditions.<br>• In our focus groups pharmacists described being unsure about when they might take an active role in skin condition management. They also wanted

For peer review only

Question	Explanatory notes
	evidence-based information about what support and products they can provide.<br><br>• This might focus upon specific conditions (eczema, psoriasis, insect bites, etc.) or could be for more generic conditions (a rash, dry skin, etc.).
7. Would dedicated resources support community pharmacists to **effectively refer skin conditions** that require urgent or more specialist attention?	• This could involve developing and/or testing resources which support community pharmacists in referring skin conditions to other sources of healthcare (e.g. emergency care, general practice, etc.).<br><br>• It might include developing referral pathways and new services for specific serious skin conditions.<br><br>• In our focus groups pharmacists described feeling uncertain about when to take this sort of action; members of the public recognised that sometimes they wanted a pharmacist to 'tell them to go to the doctor'.
8. Could a wider range of products and treatments for skin conditions be made available via community pharmacy?	• This might include considering treatments which are restricted due to legal reasons (e.g. can only be supplied against a prescription) or economic reasons (e.g. where price might inhibit purchase).<br><br>• In our focus groups both pharmacists and members of the public described frustration that certain products are restricted and cannot be provided by a pharmacist.
9. What could be done to raise awareness of the skills that community pharmacists have with regards to the identification and management of skin conditions?	• This could involve local or national campaigns to raise awareness about community pharmacy as a healthcare resource. It might focus upon how community pharmacy services are understood by members of the public.<br><br>• In our focus groups some members of the public were not aware that community pharmacists are able to provide healthcare advice about skin conditions. Some members of the public were concerned that commercial concerns might be a priority for pharmacists, i.e. selling products might be more important than providing appropriate advice.
10. How can community pharmacists work most effectively with other healthcare professionals in the identification and management of skin disease?	• This might involve new integrated services for the management of skin conditions that include community pharmacists and other healthcare providers (e.g. GPs, nurses, dermatologists). It could include the use of pharmacist independent prescribers or pharmacists with additional training in dermatology.<br><br>• In our focus groups pharmacists reported working in isolation with little or no feedback about the advice and services that they offer. They also indicated that it is sometimes difficult to contact or engage with other healthcare providers about skin conditions.

For peer review only

BMJ Open

How can community pharmacists be supported to manage skin conditions? A multi-stage stakeholder research prioritisation exercise.

Journal:	*BMJ Open*
Manuscript ID	bmjopen-2023-071863.R2
Article Type:	Original research
Date Submitted by the Author:	10-Nov-2023
Complete List of Authors:	Harvey, Jane; University of Nottingham Centre for Evidence Based Dermatology<br>Shariff, Zakia; Aston University Aston Pharmacy School<br>Anderson, Claire ; University of Nottingham School of Pharmacy<br>Boyd, Matthew; University of Nottingham School of Pharmacy, School of Pharmacy<br>Ridd, Matthew; University of Bristol Faculty of Health Sciences, Population Health Sciences<br>Santer, Miriam; University of Southampton, Primary Care Research Centre,<br>Thomas, Kim; University of Nottingham Centre for Evidence Based Dermatology, Dermatology<br>Maidment, Ian; Aston University School of Life and Health Sciences, Aston Pharmacy School<br>Leighton, Paul; University of Nottingham Centre for Evidence Based Dermatology
**Primary Subject Heading**:	Dermatology
Secondary Subject Heading:	Health services research
Keywords:	DERMATOLOGY, Patient Participation, QUALITATIVE RESEARCH, PRIMARY CARE

SCHOLARONE™
Manuscripts

I, the Submitting Author has the right to grant and does grant on behalf of all authors of the Work (as defined in the below author licence), an exclusive licence and/or a non-exclusive licence for contributions from authors who are: i) UK Crown employees; ii) where BMJ has agreed a CC-BY licence shall apply, and/or iii) in accordance with the terms applicable for US Federal Government officers or employees acting as part of their official duties; on a worldwide, perpetual, irrevocable, royalty-free basis to BMJ Publishing Group Ltd ("BMJ") its licensees and where the relevant Journal is co-owned by BMJ to the co-owners of the Journal, to publish the Work in this journal and any other BMJ products and to exploit all rights, as set out in our licence.

The Submitting Author accepts and understands that any supply made under these terms is made by BMJ to the Submitting Author unless you are acting as an employee on behalf of your employer or a postgraduate student of an affiliated institution which is paying any applicable article publishing charge ("APC") for Open Access articles. Where the Submitting Author wishes to make the Work available on an Open Access basis (and intends to pay the relevant APC), the terms of reuse of such Open Access shall be governed by a Creative Commons licence – details of these licences and which Creative Commons licence will apply to this Work are set out in our licence referred to above.

Other than as permitted in any relevant BMJ Author's Self Archiving Policies, I confirm this Work has not been accepted for publication elsewhere, is not being considered for publication elsewhere and does not duplicate material already published. I confirm all authors consent to publication of this Work and authorise the granting of this licence.

For peer review only

Title: How can community pharmacists be supported to manage skin conditions? A multi-stage stakeholder research prioritisation exercise.

Proposed journal for submission: BMJ open

Authors: J Harvey[1#], Z Shariff[2], C Anderson[3], MJ Boyd[3], MJ Ridd[4] M Santer[5], KS Thomas[1], I Maidment[2] and P Leighton[1]

Affiliations

[1]Centre of Evidence Based Dermatology, School of Medicine, University of Nottingham, Nottingham, UK
[2] Aston Pharmacy School, Aston University, Birmingham, UK.
[3] Division of Pharmacy Practice and Policy, School of Pharmacy, University of Nottingham, Nottingham, UK
[4]Population Health Sciences, University of Bristol, Bristol, UK
[5] Primary Care Research Centre, University of Southampton, Southampton, UK
# Corresponding author jane.harvey1@nottingham.ac.uk Centre of Evidence Based Dermatology, Applied Health Research Building, School of Medicine, University of Nottingham, NG7 2RD

ORCiD

J Harvey 0000-0003-1402-6116
Z Shariff 0000-0003-4521-0864
C Anderson 0000-0002-5406-2296
MJ Boyd 0000-0003-2997-5090
MJ Ridd 0000-0002-7954-8823
M Santer 0000-0001-7264-5260
KS Thomas 0000-0001-7785-7465
I Maidment 0000-0003-4152-9704
P Leighton 0000-0001-5208-0274

Word count

Abstract 298

Body of text 4678

Acknowledgements

Thank you also to all the members of pharmacy staff, other healthcare professionals and members of the public that took part in interviews or workshops. Particular thanks goes to Amanda Roberts and our other patient representative for their help with this work.

For peer review only

Exclusive Licence

I, the Submitting Author has the right to grant and does grant on behalf of all authors of the Work (as defined in the below author licence), an exclusive licence and/or a non-exclusive licence for contributions from authors who are: i) UK Crown employees; ii) where BMJ has agreed a CC-BY licence shall apply, and/or iii) in accordance with the terms applicable for US Federal Government officers or employees acting as part of their official duties; on a worldwide, perpetual, irrevocable, royalty-free basis to BMJ Publishing Group Ltd ("BMJ") its licensees and where the relevant Journal is co-owned by BMJ to the co-owners of the Journal, to publish the Work in BMJ Open and any other BMJ products and to exploit all rights, as set out in our licence.

The Submitting Author accepts and understands that any supply made under these terms is made by BMJ to the Submitting Author unless you are acting as an employee on behalf of your employer or a postgraduate student of an affiliated institution which is paying any applicable article publishing charge ("APC") for Open Access articles. Where the Submitting Author wishes to make the Work available on an Open Access basis (and intends to pay the relevant APC), the terms of reuse of such Open Access shall be governed by a Creative Commons licence – details of these licences and which Creative Commons licence will apply to this Work are set out in our licence referred to above.

For peer review only

ABSTRACT

Objective

To establish research priorities which will support the development and delivery of community pharmacy initiatives for the management of skin conditions.

Design

An iterative, multi-stage stakeholder consultation consisting of online survey, participant workshops, and prioritisation meeting.

Setting

All data collection took place online with participants completing a survey (delivered via the JISC Online Survey platform, between July 2021 and January 2022) and participating in online workshops and meetings (hosted on Microsoft Teams between April and July 2022).

Participants

174 community pharmacists and pharmacy staff completed the online survey.

53 participants participated in the exploratory workshops (19 community pharmacists, 4 non pharmacist members of pharmacy staff and 30 members of the public). 4 healthcare professionals who were unable to attend a workshop participated in a one-to-one interview.

29 participants from the workshops took part in the prioritisation meeting (5 pharmacists/pharmacy staff, 1 other healthcare professional, and 23 members of the public).

Results

Five broad areas of potential research need were identified in the online survey:(1) identifying and diagnosing skin conditions; (2) skin conditions in skin of colour; (3) when to refer skin conditions; (4) disease specific concerns; and (5) product specific concerns.

These were explored and refined in the workshops to establish ten potential areas for research which will support pharmacists in managing skin conditions. These were ranked in the prioritisation meeting. Amongst those prioritised were topics which consider how pharmacists work with other healthcare professionals to identify and manage skin conditions.

Conclusions

Survey responses and stakeholder workshops all recognised the potential for community pharmacists to play an active role in the management of common skin conditions. Future research may support this in the generation of resources for pharmacists, in encouraging public take-up of pharmacy services, and in evaluating the most effective provision for dealing with skin conditions.

For peer review only

104 **STRENGTHS AND LIMITATIONS**

105 • Novel exploration of the research needs associated with the care of skin conditions within community pharmacy.
107 • An iterative, multi-stage consultation ensured detailed insight about the topic.
108 • The involvement of pharmacists, pharmacy staff, healthcare professionals and members of the public ensured that all pertinent voices were heard.
110 • Participants were self-selecting and may have had a particular interest/perspective upon skin conditions.
112 • Greater participation from pharmacists in the prioritisation workshops may have been beneficial.

114

For peer review only

INTRODUCTION

Community pharmacy is recognised as an accessible source of healthcare advice [1-3] and the COVID pandemic has cemented it more clearly in the primary care landscape for members of the public [4]. Moreover, recent initiatives, such as the Community Pharmacy Consultation Service (CPCS), seek to use pharmacy more effectively by diverting the management of some minor ailments to community pharmacy settings [5].

Skin conditions are amongst the most common diseases encountered by healthcare professionals[6,7]. Each year approximately 54% of the population will experience some form of skin disease[6], at any one time up to one-third of all people will have a skin condition that warrants medical attention[6,8,9]. Skin complaints have been identified as conditions that could be potentially managed within community pharmacy [10,11] and community pharmacists recognise skin conditions as a significant part of their workload [1,12,13]. Pharmacists regularly give advice on the management of common conditions such as eczema, dermatitis, generalised rashes, allergies and acne [12] and just over one-third (38%) of all symptomatic advice requests in community pharmacy relate to skin conditions[14]. Almost 20% of pharmacy sales are for skin products[2].

Due to the current stresses faced by the National Health Service, Community pharmacy in the U.K. is developing at a fast pace. Within England pharmacists are involved in treating dermatological conditions through the provision of a number of services. These include the CPCS, introduced in November 2020, where a GP surgery or NHS 111 can refer patients to community pharmacies for the treatment of minor illness, for example skin rashes[15]. Additionally, pharmacists may treat patients with skin conditions, free of charge, through minor ailments schemes but this provision varies in availability between areas[16]. In some areas specially trained pharmacists have access to prescription only medications through the use of patient group directions (PGDs) for certain conditions such as infected eczema or infected insect bites[17]. Most recently the government announced that a "Pharmacy First" scheme will be introduced within England. Through this scheme pharmacists will be able to prescribe medications (through PGDs) to treat conditions such as impetigo, shingles and infected insect bites[18]. In Wales and Scotland, the pharmacy first scheme has already been implemented. In these areas medications can also be provided via PGDs or through independent (non-medical) prescribers[19]. For example, in Scotland there are PGDs available for medications to treat impetigo, shingles and skin infections[20].

Therefore, within the UK context pharmacists are already involved in the diagnosis and treatment of skin conditions and this involvement has accelerated in the past few years. As community pharmacy continues its trajectory towards expanded and extended provision[3] research will demonstrate the effectiveness of new ways of working and will support the development of new evidence-based services and resources. [6,8,21-23]

The aim of this work is to establish stakeholder consensus upon those research priorities which might best support community pharmacists in their involvement in the care of patients with skin conditions.

METHODS

This was a multi-stage, iterative stakeholder consultation informed by James Lind Priority Setting Partnership method [24] consisting of: (1) an online survey, (2) exploratory workshops, and (3) a prioritisation workshop.

For peer review only

Participants

Stage 1 – online survey

An online survey using the JISC Online Survey platform (https://www.jisc.ac.uk/online-surveys#) was targeted to community pharmacists and other community pharmacy staff. Social media (Twitter® and Facebook) and personal and professional networks (e.g., Pharmaceutical Services Negotiating Committee (PSNC) newsletter) were used to promote the survey. The survey was opportunistic and there were no specific inclusion criteria i.e. all pharmacists (and other members of pharmacy staff) were eligible to complete the survey.

A specific analysis of the survey data has been submitted for publication elsewhere.

Stage 2 – exploratory workshops

Community pharmacists, pharmacy staff, other healthcare professionals, and members of the public were recruited to a series of workshops to explore potential research topics which might support the management of skin conditions in community pharmacy. Equal numbers of public and professional participants were sought.

Social media (Twitter® and Facebook) and personal and professional networks (e.g., Community Pharmacy Dermatology Network, primary care networks) were again used to recruit pharmacists, pharmacy staff as well as healthcare professionals (e.g., GPs, specialist nurse practitioners). Additional professional networks (the Primary Care Dermatology Society, the Society for Academic Primary Care Skin Special Interest Group and the UK Dermatology Clinical Trials Network) were used to recruit healthcare professionals.

Members of the public were recruited via social media and existing public and patient research networks (e.g. the CEBD patient panel and "People in Research" (https://www.peopleinresearch.org/)). All members of public who expressed an interest in the project were invited to join focus groups regardless of their experience of skin conditions or pharmacies.

Where any individual was not able to attend a scheduled workshop they were offered the opportunity to take part in a brief one-to-one interview.

Stage 3 – prioritisation workshops

Exploratory workshop participants were subsequently invited to take part in the prioritisation workshop, with the goal of equal numbers of public and professional participants.

Data collection and analysis

Stage 1 – online survey

Survey responses were collected over a period of six months between 20th July 2021 and 20th January 2022.

Content analysis of free text responses was used to identify commonly used words and phrases. Selected words or phrases (frequently used or substantively important) were reviewed thematically[25].

Stage 2 - exploratory workshops and interviews

Workshops and interviews were undertaken online using Microsoft Teams and took place between April and July 2022. Up to six workshops were planned, to ensure 40 – 60 participants in this phase.

For peer review

They were structured according to stage 1 data, with key themes explored further through group discussion. Discussion focused explicitly upon "research priorities"; although notions such as "barriers", "facilitators" and "challenges" were also used to make discussions less abstract and to support broad participation. See Supplementary files 1 and 2 for workshop schedules.

All discussions were digitally recorded with permission. Digital recordings were automatically transcribed verbatim and anonymised.

Framework analysis[26] was used to map workshop and interview data to broad uncertainties identified in stage 1. Synthesis of data and interpretation of synthesised data led to the creation of narrower research topics.

Stage 3 - prioritisation workshop

Following the conventions of the Nominal Group Technique[27] research topics were shared with participants prior to the prioritisation workshop. During the workshop group discussion and item scoring were used iteratively to reject and rank topics. Simple, descriptive statistics were used to rank and establish consensus upon priority research topics (i.e., the percentage of respondents selecting a topic for inclusion / priority). For further information regarding methods please see Supplementary file 3.

Patient and Public Involvement

Before the study started, we met with two PPI (patient and public involvement) collaborators to provide an overview of the study and the study methodology. One PPI member collaborated with us to develop the participant information leaflet. They also attended the steering group meeting where we developed the final list of research questions. The other PPI collaborator assisted with recruitment of patients via social media and recommended other areas where we could recruit participants e.g. the "people in research" website. They also attended one of our patient focus groups.

RESULTS

The numbers and characteristics of participants that took place at each stage of the process are shown in Table 1.

Table 1 Numbers of participants included at each stage of the priority setting exercise

	**Numbers of participants**		
	Stage One	Stage Two	Stage Three
**Type of participant**			
Patients	N/A	30	23
Pharmacists	111	19	3
Other members of pharmacy staff	63	4	2
Specialist dermatology nurse	N/A	1	1
GPs	N/A	3	0

Stage 1 – online survey

The survey was completed by 174 participants. Word counts and an example of the word trees are available in Supplementary file 4.

The most reported five words in response to the "challenge" questions were "refer, rash, products, differential and know" whilst in the "research priorities" question, the most reported words were "treatment, different, products, need and creams". These and the remaining top 20 words from each question encompass a broad range of research

For peer review only

challenges, which were reflected in the five key areas of the analytic framework detailed in Table 2.

Table 2 Original analytic framework developed from survey responses

Identifying and diagnosing skin conditions
Skin of colour
Knowing when to refer skin conditions to a GP
Disease specific concerns
Product specific concerns

Stage 2 – exploratory workshops and one to one interviews

Nine workshops were held, and four additional interviews to facilitate those unable to attend a scheduled workshop. Workshops lasted between one and two hours, interviews were typically around thirty minutes.

Four workshops consisted of pharmacists (19 participants), one included only pharmacy staff (4 participants), and four workshops contained only members of the public (30 participants). Interviews were undertaken with three GPs and one dermatology nurse specialist (Table 1).

Data is presented here thematically, pointing to key uncertainties and research possibilities that these themes suggest (further examples of the data are available in Supplementary file 5).

Theme 1 – identifying and diagnosing skin conditions.

The challenge of identifying and diagnosing skin conditions was a common focus and frequently described as a source of stress:

> *"Skins a nightmare"*
> *(Workshop 2, Pharmacist 1)*

> *"One of the worst things I can hear in a pharmacy is when a patient says, "can I speak to the pharmacist? Can they tell me what this rash is my child has got?""*
> *(Workshop 2, Pharmacist 2)*

Difficulties identifying *reliable* resources were recognised. Google images, the National Health Service (NHS) website, Clinical Knowledge Summaries website (CKS) or National Institute for Health and Care Excellent (NICE) guidelines were all discussed, but using *standard* photographs was not always found to be helpful. That some resources (e.g., CKS and NICE) do not contain images further impacts upon their utility.

Pharmacists explained that this is particularly an issue with skin conditions as members of the public commonly show them affected skin, rather than verbally describing symptoms as they do with other conditions.

The development of pharmacy specific resources (e.g., online toolkits, in person training etc.) was recognised as a potentially important area for future research and action.

> Possible Research Question - Would dedicated resources improve the identification of skin conditions in community pharmacy?

For peer review only

Theme 2 - Identifying and diagnosing skin conditions in skin of colour

Discussion of skin of colour proceeded almost as an extension of theme 1. With a few exceptions, most participants described identifying skin conditions in skin of colour as more difficult:

> *"I know fungal infection definitely look[s] different on like very dark skin, but I don't know whether my diagnosis would be right, so I'm just always doubting myself. Yeah, so one other thing is, um, discoid eczema [and] ringworm they look very similar on like dark skin or fair skin. So that comes up all the time. I get asked whether it's eczema ringworm all the time. And I don't know the difference."*
> *(Workshop 1, Pharmacist 3)*

Knowing when a condition was getting worse was also considered more challenging in skin of colour. Again, an absence of *reliable, high-quality, evidence-based* resources was considered a barrier to effectively responding to queries and questions.

> Possible Research Question - Would dedicated resources improve the identification of skin conditions in skin of colour in community pharmacy?

Theme 3 - Knowing when to refer skin conditions.

Members of the public described using community pharmacy as a form of triage, seeking advice about whether a condition was "*serious enough*" to consult other healthcare professionals. For some a pharmacist's advice had been an important factor in being confident enough to seek a doctor's appointment.

This was a role that pharmacists recognised but were not always comfortable with; they had specific concerns about *delaying diagnosis* of serious conditions, *missing infectious diseases* or a fear of *making a condition worse* by giving the wrong advice:

> *"Some condition can wait for next day or next week, but some condition need to be managed quite soon. Like same day referral. So I think my challenge was whether to refer […] because weekend 111 is so busy they take hours for them to the doctor they call them back. So sometimes they like go to walk in centre or wait. So I think it's either they can wait till next day or next few days or. With that same day, it's my challenge."*
> *(Workshop 1, Pharmacist 3)*

These concerns were considered more critical if advice was being sought about a child.

Pharmacists also identified that it was not always easy to contact other health professionals and that it is difficult to know when and how to refer patients. The potential for better connected services in the management of skin conditions was also recognised in one of our interviews (with a GP), although it was also recognised that resources might be a barrier to this.

> Possible Research Question - Would dedicated resources support community pharmacists to effectively refer skin conditions that require urgent or more specialist attention?

Theme 4 - Disease specific concerns

Workshop discussion did not confirm such a strong focus upon specific skin conditions as the survey data, but rather pointed to general challenges of managing skin conditions in community pharmacy. An absence of feedback, and of knowing the outcome of advice was commonly described.

For peer review only

> *"So even though I've kind of recommended this steroid, or I've recommended this emollient, I don't know whether it worked or not because they just don't come back. Even if it's a regular customer."*
> *(Workshop 2, Pharmacist 4)*

This makes it harder for a pharmacist to feel fully confident in the advice that they are providing. Similarly, pharmacists rarely gained feedback when referring an individual to a GP, although subsequently seeing the GP's prescriptions might offer some informal insight.

The potential for pharmacists to be more involved in managing skin disease was commonly recognised in both the pharmacist workshops as well as healthcare professional interviews. A few suggested that this might be in diagnosing and suggesting initial treatments, others focused upon counselling on long-term medication use:

> *"Perhaps a bigger and perhaps more important role for pharmacists is actually in supporting patients with long term chronic skin conditions. Because there are loads of people out there with eczema, acne, psoriasis and so on who don't really get the best out of their treatment and end up going into secondary care because they are very poorly managed … I think this is a golden opportunity for community pharmacists to get more involved is actually in supporting those patients."*
> *(Workshop 1, Pharmacist 5)*

> Possible research question - How can community pharmacists work most effectively with other healthcare professionals in the identification and management of skin disease?

Theme 5 - Product specific concerns

During the workshops pharmacists communicated that they were confident about their knowledge of products used to manage skin conditions. Members of the public reinforced the importance of this by communicating that they expected pharmacists to understand the products that they were providing:

> *"It seems to me that a pharmacist should be an expert on the products. And if they're not already an expert on the products then one questions what they're doing as a pharmacist. Sorry"*
> *(Workshop 5, Patient 1)*

During the pharmacist workshops some frustration was communicated about not being allowed to provide certain products over the counter, products that customers would subsequently receive on prescription from their GPs:

> *"I do recognize some or quite a few skin conditions, I would like to give them something that is prescription only, but I can't. So then I have to send them off to the GP, so I would personally like some sort of PGD [Patient Group Direction] or guidelines to be able to prescribe [erm], to do a course be accredited and to be able to prescribe that maybe not to have to go through the whole performance of becoming an independent prescriber, because I don't have the time or the facility to do that."*
> *(Workshop 4, Pharmacist 6)*

Some members of the public were equally frustrated by this, confused about why they could order medications from the internet but not access them directly via community pharmacies. Topical corticosteroids (TCS) were often discussed in this way. Members of

For peer review only

the public described how they had lied about how they were going to use TCS to ensure that it was provided:

> *"the only time it was mentioned [topical corticosteroids] was when they refused to sell me it, you know, and that that that sounds stupid. It was, you know, when I've got it on prescription, there's never been any query or any conversation about it. It's just been given in a bag. [And] But when I needed to actually purchase something over the counter. And that's when the interrogation started."*
> *(Workshop 5, Patient 2)*

The centrality of product knowledge suggests that it could be an important area for research and resource development. The potential to extend what pharmacists can do may be important in this.

> Possible research question - Could a wider range of products and treatments for skin conditions be made available via community pharmacy?

> Possible research questions - Would dedicated resources support community pharmacists in the management of skin conditions?

Theme 6 – Other topics

Discussion of the themes identified in the online survey often prompted a broader discussion of community pharmacy and skin conditions. This led to the identification of additional areas where research might be warranted.

In the workshops pharmacists reinforced the notion that they see a broad range of skin conditions daily, and that the number of customers seeking advice about skin conditions is increasing.

> *"I think the numbers of skin referrals with the CPCS* [Community Pharmacy Consultation Service] *is going to go up into Community pharmacy because it's one thing that the GPs can triage without seeing."*
> *(Workshop 4, Pharmacist 7)*

Pharmacists, however, were less confident in making an assessment about how demand is growing and evolving, e.g. which clinical conditions, what types of enquiry, specific demographic groups, adults/children, etc. Discussion in the workshops, as well as some one-to-one interviews, recognised that understanding trends in demand could be an important precursor to any substantive change in how community pharmacy works or engages with skin conditions.

> Possible research question - In what ways are community pharmacists currently involved in the identification and management of skin conditions?

Some of these discussions (especially in the pharmacist workshop) exposed localised variation in what is available and what pharmacists are allowed to do.

> *"We're lucky like, in England, as you have heard you saying that you guys have to charge your folk for it. In the pharmacy first, one thing we've got, is we can give it out free of charge and they can come back six times and get six different bottles and it doesn't cost them anything"*
> *(Workshop 2, Pharmacist 1)*

Discussion demonstrated that variations are manifest in: (i) the skin conditions that pharmacists are paid to treat via minor ailments schemes; (ii) the medications which can be provided via the use of patient group directions (PGDs); and (iii) whether patients had to pay for their treatment. As with understanding trends in demand it was

For peer review only

considered pertinent to develop a better understanding of the success of current skin focused initiatives and ways of working as a precursor to any further development.

> Possible research question - What are the known benefits of community pharmacy involvement in the identification and management of skin conditions?

The public workshops offered a slightly different perspective on this topic, focusing upon establishing that pharmacists are appropriately qualified and competent to deal with skin conditions.

> *"It just feels that pharmacists know a lot about their medicines and of course they know the pros and cons of uses, but whether they have got the expertise in recognizing a particular type of rash or a particular type of mark on the skin […] I wouldn't know if they had that expertise."*
> *(Workshop 7, Patient 3)*

Similar concerns were expressed in the healthcare professional interviews, with one GP indicating that they saw "a lot of inappropriate" referrals from pharmacists.

Once again, the benefit of establishing the state of current provision for skin conditions in community pharmacy was recognised as an appropriate focus.

> Possible research question - How competent are community pharmacists in the identification and management of skin conditions?

Discussion of direct experience of accessing community pharmacy exposed that participants in the public workshops were often polarised, between those that regularly used their pharmacist and those that were not aware that pharmacists offered this type of service.

> *"I never knew that they could advise you on skin you know problems, I never knew that. Because I thought they dealt dealt with drugs only and they all seemed very busy. So, I'd like to know how the pharmacist can help with skin conditions as well?"*
> *(Workshop 6, Patient 4)*

A concern about a lack of awareness about pharmacy services was echoed in the healthcare professional interviews, where GPs described difficulties convincing patients that minor ailments schemes are appropriate to use. Identifying barriers and encouraging the use of community pharmacy might be important research that underpins the effectiveness of any specific initiative:

> Possible research question - What could be done to raise awareness of the skills that community pharmacists have with regards to the identification and management of skin conditions?

A list of all ten research questions is provided in Table 3 (see online Supplementary File 6 for research questions with explanatory notes).

For peer review only

Table 3 The ten research questions from the community pharmacy and dermatology priority setting partnership (in no particular order, prioritised questions in bold)

1. **Would dedicated resources improve the identification of skin conditions in community pharmacy?**
2. Would dedicated resources improve the identification of skin conditions in skin of colour in community pharmacy?
3. Would dedicated resources support community pharmacists to effectively refer skin conditions that require urgent or more specialist attention?
4. **How can community pharmacists work most effectively with other healthcare professionals in the identification and management of skin disease?**
5. Could a wider range of products and treatments for skin conditions be made available via community pharmacy?
6. Would dedicated resources support community pharmacists in the management of skin conditions?
7. In what ways are community pharmacists currently involved in the identification and management of skin conditions?
8. What are the known benefits of community pharmacy involvement in the identification and management of skin conditions?
9. **How competent are community pharmacists in the identification and management of skin conditions?**
10. What could be done to raise awareness of the skills that community pharmacists have with regards to the identification and management of skin conditions?

Stage 3 - prioritisation workshops

To accommodate all those interested in participating, the prioritisation workshop was split into two parts which ran consecutively. The first prioritisation workshops included one dermatology nurse specialist and 10 patients. The second prioritisation workshop included three pharmacists, two members of pharmacy staff and 13 patients (Table 2). Scores at the end of the first workshop were carried forward as the starting point for the second.

At the conclusion of the second workshop the following questions were voted to be the most important (3 participants did not vote):

1. How can community pharmacists work most effectively with other healthcare professionals in the identification and management of skin disease? 11/15 participants
2. How competent are community pharmacists in the identification and management of skin conditions? 9/15 participants
3. Would dedicated resources improve the identification of skin conditions in community pharmacy? 6/15

For peer review only

DISCUSSION

Summary

Through consultation with a range of stakeholders including pharmacists, pharmacy staff, GPs, dermatology nurses and members of the public we have developed a set of research questions to support dermatology provision in community pharmacy. In a final prioritisation exercise we have established three topics as a starting point for a dermatology/community pharmacy research agenda.

Discussion in our workshops reinforced existing assessment that dermatology is a significant part of the workload faced by community pharmacists [12,14,28]. It also reinforced the expectation that this demand is likely to grow in future. Concerns about limited training and knowledge about skin conditions [1] were evident in comments about lacking confidence in dealing with skin queries. A perceived lack of resources, training and post-consultation feedback were recognised as factors in this. Developing resources for pharmacists/pharmacy staff might be an important area where research can benefit pharmacy practice – resources in this context might be training programmes, information resources for staff, information resources for the public, and they could be delivered in print, in person or online.

Research uncertainties identified here might suggest the value of repeating and/or expanding prior research which has considered pharmacists' ability to identify skin conditions [22,23].

Our findings also reinforce more general issues of public awareness about the role of community pharmacy [29,30], specifically recognising this to be an issue with regards to skin conditions. Previous work has identified that individuals felt that only doctors are "qualified/trustworthy" to manage skin complaints [11], a view also expressed by medicine counter assistants[1]. This is a particularly important challenge to negotiate given that all stakeholders who took part in the exercise recognised that the demand in primary care exceeds what GPs are able to manage. However, as there is currently a shortfall in the number of pharmacists and pharmacy staff, it is possible that, under the current workforce model pharmacists may also struggle to meet this demand[31].

Further information about the questions

We have developed a broad range of questions reflecting a broad range of concerns described in the workshops. It is important to note that though we have highlighted these questions for future research, we have not conducted a systematic review to check whether there is already research that addresses these issues.

We have framed questions loosely and in general terms to allow interpretation and wide scope for impact. For example, the final question, "*How can community pharmacists work most effectively with other healthcare professionals in the identification and management of skin disease?*" could be approached in terms of consideration of the appropriate prescribing of "over the counter" medicines or in terms of the provision of information for regarding the use of long-term prescription medications.

In this, however, we might suggest that there is a form of natural hierarchy with questions focused upon understanding current provision a necessary precursor to research which develops new ways of working. For example, answering, "*in what ways are community pharmacists currently involved in the identification and management of skin conditions?*", would allow future work to be appropriately directed to the most common or most difficult to manage skin conditions.

We would encourage researchers to develop the focus of research in meaningful ways, mindful of the changing landscape within community pharmacy with initiatives such as

For peer review only

the independent prescribing schemes in England[3]. We would also encourage researchers to consider how resources directed towards community pharmacists can deliver consistent messages to other health care professionals.

The term diagnosis in relation to pharmacist-led activity may not be as widely understand across the globe. However, our survey found identifying and diagnosing skin conditions a key area for further research. This research could include the role of the pharmacist is diagnosing conditions compared to simply identifying them.

Strengths and limitations

This has been a broad reaching exercise which has included 111 community pharmacists in an online survey as well as 57 workshop and interview participants. Workshops sought input from both healthcare professionals as well as members of the public – numbers were approximately even in the exploratory workshops (27 healthcare professionals / 30 members of the public).

Whilst we are confident that a broad range of perspectives were considered in the identification of research topics the final prioritisation stage was heavily weighted towards the input of members of the public. At this stage of our process the goal of equal numbers was not achieved, with only five pharmacists/pharmacy staff participating in the prioritisation stage (despite twelve signing-up).

Overall, we were only able to recruit 3 GPs and one specialist dermatology nurse to take part in interviews. We did not include dermatologists or decision makers in the project, reflecting our primary care focus. Further work in this area could explore different methods of engaging with GPs, other stakeholders and pharmacists to improve recruitment. We should consequently recognise that the final prioritisation more accurately reflects the public view of what research would be most beneficial, rather than a multi-perspective assessment of this.

We might also acknowledge that the qualitative nature of much of the data generated here necessarily required interpreting as part of data analysis – it may be that in our interpretations we found questions and uncertainties that were not intended by participants. The iterative nature of the study with a final workshop specifically focused upon research uncertainties hopefully tempers this process.

CONCLUSION

Pharmacists are regularly consulted regarding skin conditions and do not always feel confident in the identification of skin disease. Using information from focus groups with pharmacists, members of the public and other stakeholders we developed 10 research questions that can be used to direct future research to address these challenges.

For peer review only

ETHICAL APPROVAL STATEMENT

This study was conducted in two parts. The survey was administered by Aston University and the workshops/interviews by the University of Nottingham.

We included a copy of the Ethical Approval Letter from the Aston University Ethical Committee with regards to the survey in the submission of this article. Participants completing the survey were required to complete an online consent form.

We also provided a copy of a letter from the University of Nottingham Faculty of Medicine and Health Sciences stating ethical approval was not required for this part of the study. Participants did not complete a written consent form in line with the fact this was not judged to be research, however, they were asked at the start of the focus groups/interviews whether they were happy to be recorded.

FUNDING STATEMENT

This project is funded by the National Institute for Health and Care Research (NIHR) School for Primary Care Research (project reference 522).

The views expressed are those of the author(s) and not necessarily those of the NIHR or the Department of Health and Social Care.

CONFLICTS OF INTEREST

JH, IM, CA, KST, PL, ZS have declared they have no conflict of interest.

MB has received personal fees from Delphi Healthcare outside the submitted work, has grants or contracts with Health Education England (Grant for development of experimental learning activity)and Walgreen Boots Alliance (50% funding for PhD studentship). MB has received consulting fees from Clinical Care Quality Solutions (payment split between institution and author). MB has received payment from the Ministry of Health Singapore, Human Manpower Development programme (honoraria to speak including travel and accommodation). MB is a Project advisor/Chair of experiential learning during the MPharm research project. NHS Scotland (payment to institution and author). MB is Vice Chair Pharmacy Law and Ethics Association (no fee received).

MS has the following Grants or contracts:

RAPID and Efficient Eczema Trials (RAPID programme) – lead applicants Thomas and Roberts. NIHR PGfAR NIHR203279 funding to University of Southampton for 5% of my time.

Trial of IGe tests for Eczema Relief (TIGER): randomised controlled trial of test-guided dietary advice for children with eczema, with internal pilot and nested economic and process evaluations – lead applicant Ridd. NIHR HTA NIHR133464 funding to University of Southampton for 10% of my time

Pragmatic, primary care, multi-centre, randomised superiority trial of four emollients in children with eczema, with internal pilot and nested qualitative study (Best Emollients for Eczema - BEE) – lead applicant Ridd. NIHR HTA 15/130/07 completed Aug 2020 funding to University of Southampton for 10% of my time.

MS is a Funding panel member NIHR Programme Grants for Applied Research 2018 to present day and also Academic PPIE lead and Board Member NIHR School for Primary Care Research 2022 to present day.

For peer review only

MR has received Various NIHR grants for studies of skin conditions/food allergy. MR is On TSC/DMC for ERICA, PRINCIPLE and ALPHA trials and is Co-Chair SAPC & NIHR SPCR skin/allergy research groups.

CONTRIBUTIONS

ZS and IM conceptualised, designed and conducted the survey, ZS and JH analysed the survey data, JH and PL led the workshops and interviews, analysis of the interview and workshop data and drafted the manuscript, comments were made on the draft by ZS, IM, MS, MR, MB, KST and CA. All authors commented on and approved the final draft.

DATA SHARING STATEMENT

Data is available from the authors on request.

For peer review only

616

For peer review only

REFERENCES

1. Tucker R, Stewart D. An exploratory study of the views of community pharmacy staff on the management of patients with undiagnosed skin problems. *Int J Pharm Pract* 2015;23(6):390-98. doi: 10.1111/ijpp.12179
2. Tucker R, Stewart D. Why people seek advice from community pharmacies about skin problems. *Int J Pharm Pract* 2015;23(2):150-53. doi: 10.1111/ijpp.12126
3. Medicines and Pharmacy Directorate - Department of Health and Social Care. The Community Pharmacy Contractual Framework for 2019/20 to 2023/24: supporting delivery for the NHS Long Term Plan. London2019 [Available from: https://assets.publishing.service.gov.uk/government/uploads/system/uploads/attachment_data/file/819601/cpcf-2019-to-2024.pdf accessed April 2022.
4. Delgutte A, Santos D. Community pharmacy has been an indispensable element of the covid-19 response BMJ online; [Available from: https://blogs.bmj.com/bmj/2021/02/21/community-pharmacy-has-been-an-indispensable-element-of-the-covid-19-response/#:~:text=Community%20pharmacy%20has%20been%20an%20indispensable%20element%20of%20the%20covid%2D19%20response,-February%2021%2C%202021&text=Community%20pharmacists%20are%20trained%20to,rest%20of%20the%20healthcare%20system accessed April 2022.
5. NHS England. NHS Community Pharmacist Consultation Service (CPCS) – integrating pharmacy into urgent care [Available from: https://www.england.nhs.uk/primary-care/pharmacy/pharmacy-integration-fund/community-pharmacist-consultation-service/ accessed October 2022.
6. The King's Fund. How can dermatology services meet current and future patient needs while ensuring that quality of care is not compromised and that access is equitable across the UK? . London, 2014.
7. Le Roux E, Edwards PJ, Sanderson E, et al. The content and conduct of GP consultations for dermatology problems: a cross-sectional study. *British Journal of General Practice* 2020;70(699):e723-e30. doi: 10.3399/bjgp20X712577
8. Schofield J, Grindlay D, Williams H. Skin conditions in the UK : a health care needs assessment: Centre of Evidence Based Dermatology Nottingham, 2009.
9. Eedy D. The crisis in dermatology. *BMJ* 2015;350:h2765. doi: 10.1136/bmj.h2765
10. Fielding S, Porteous T, Ferguson J, et al. Estimating the burden of minor ailment consultations in general practices and emergency departments through retrospective review of routine data in North East Scotland. *Fam Pract* 2015;32(2):165-72. doi: 10.1093/fampra/cmv003
11. Hammond T, Clatworthy J, Horne R. Patients' use of GPs and community pharmacists in minor illness: a cross-sectional questionnaire-based study. *Fam Pract* 2004;21(2):146-9. doi: 10.1093/fampra/cmh207
12. Tucker R. Community pharmacists' perceptions of the skin conditions they encounter and how they view their role in dermatological care. *Int J Pharm Pract* 2012;20(5):344-6. doi: 10.1111/j.2042-7174.2012.00212.x
13. Tucker R. An exploratory study of uk community pharmacist's perceptions of the skin conditions they encounter: prevalence, reasons for referral and over-the-counter dermatological wish list. *Selfcare* 2013;4(1):3-17.
14. Dodds L, Katusiime B, Shamim A, et al. An investigation into the number and nature of the urgent care consultations managed and referred by community pharmacists in South-East England. *Prim Health Care Res Dev* 2020;21:e5. doi: 10.1017/S1463423620000031
15. NHS Business Services Authority. NHS Community Pharmacist Consultation Service (Minor Illness and Urgent Repeat Medicines Supply pathways) 2020 [Available from: https://www.nhsbsa.nhs.uk/pharmacies-gp-practices-and-appliance-contractors/dispensing-contractors-information/nhs-community-pharmacist-consultation-service-minor-illness-and-urgent-repeat-medicines-supply accessed 9th June 2023.

16. Wagner A, Noyce PR, Ashcroft DM. Changing patient consultation patterns in primary care: an investigation of uptake of the Minor Ailments Service in Scotland. *Health Policy* 2011;99(1):44-51. doi: https://doi.org/10.1016/j.healthpol.2010.07.002
17. Community Pharmacy England. Service case study: the Midlands Community Pharmacy Extended Care Service 2022 [Available from: https://cpe.org.uk/lpcs-and-local/locally-commissioned-services/services-case-studies-hub/service-case-study-the-midlands-community-pharmacy-extended-care-service/ accessed 9th June 2023.
18. The Pharmaceutical Journal. Pharmacy First will be introduced in England in 2023, says government. 2023;310(7973) doi: 10.1211/PJ.2023.1.184384
19. The Pharmaceutical Journal. When will England get a 'Pharmacy First' service? 2023;310(7969) doi: DOI:10.1211/PJ.2022.1.126642
20. NHS Scotland. NHS Pharmacy First Scotland Service 2020 [Available from: https://www.communitypharmacy.scot.nhs.uk/nhs-boards/nhs-lothian/pharmacy-first/ accessed 9th June 2023.
21. Rubsam ML, Esch M, Baum E, et al. Diagnosing skin disease in primary care: a qualitative study of GPs' approaches. *Fam Pract* 2015;32(5):591-5. doi: 10.1093/fampra/cmv056
22. Tucker R, K. M, V. P, et al. An exploratory study of community pharmacist diagnosis and management of dermatitis and acne. *Selfcare* 2017;8(2):1-10.
23. Tucker R, Patel M, Layton AL, et al. An exploratory study demonstrating the diagnostic ability of healthcare professionals in primary care using online case studies for common skin conditions. *Int J Pharm Pract* 2014;22(2):119-24. doi: 10.1111/ijpp.12045
24. Nygaard A, Halvorsrud L, Linnerud S, et al. The James Lind Alliance process approach: scoping review. *BMJ Open* 2019;9(8):e027473. doi: 10.1136/bmjopen-2018-027473
25. Hsieh HF, Shannon SE. Three approaches to qualitative content analysis. *Qual Health Res* 2005;15(9):1277-88. doi: 10.1177/1049732305276687
26. Gale NK, Heath G, Cameron E, et al. Using the framework method for the analysis of qualitative data in multi-disciplinary health research. *BMC Med Res Methodol* 2013;13:117. doi: 10.1186/1471-2288-13-117
27. Delbecq AL, Van de Ven AH. A Group Process Model for Problem Identification and Program Planning. *The Journal of Applied Behavioral Science* 1971;7(4):466-92. doi: 10.1177/002188637100700404
28. Hafejee A, Coulson IH. Community pharmacists' role in managing common skin problems. *Br J Dermatol* 2006;155(6):1297. doi: 10.1111/j.1365-2133.2006.07545.x
29. Latif A, Waring J, Watmough D, et al. Examination of England's New Medicine Service (NMS) of complex health care interventions in community pharmacy. *Res Social Adm Pharm* 2016;12(6):966-89. doi: 10.1016/j.sapharm.2015.12.007
30. Latif A, Waring J, Watmough D, et al. 'I expected just to walk in, get my tablets and then walk out': on framing new community pharmacy services in the English healthcare system. *Sociol Health Illn* 2018;40(6):1019-36. doi: 10.1111/1467-9566.12739
31. Company Chemists Association. Review of the Community Pharmacist Workforce in England 2022 [Available from: https://thecca.org.uk/cca-publishes-review-of-the-community-pharmacist-workforce-in-england/ accessed December 2022.

For peer review only

Derm/Comm Pharm PSP Workshop 1 – Community Pharmacists

7pm WELCOME & INTRODUCTIONS FROM US.

- Thank-you for coming.
- Introductions from PL / JH.
- Purpose of the project.

The purpose of this project is to identify uncertainties that community pharmacists experience when they are faced with someone seeking help about a skin condition.

Our goal is to identify a small number of research projects which could be undertaken to support community pharmacists - these could be trialling new ways of working, developing information resources, understanding the challenges of seeing skin conditions in community pharmacy, testing treatments, etc.

- Purpose of this evening.

From the first stage of the project, an online survey of community pharmacists, we have identified several areas that we would like to understand further, outlined below.

- Rules of engagement.
- Recording start.

7.10pm AROUND PARTICIPANTS – INTRODUCTIONS.

- name, where from, pharmacist role/background.
- 1 thing about skin….

Is there one thing that you would like to know that would help you when dealing with a skin condition / skin condition query?

7.25pm YOUR SCORING OF THE MATERIALS SENT.

7.30pm TOPIC 1.

7.45pm TOPIC 2.

8.00pm SHORT BREAK.

8.10pm OTHER TOPICS AND QUESTIONS.

8.40pm RANKING/SCORING.

8.50pm. CLOSING COMMENTS AND THANKS.

- vouchers.
- final workshop date and possibilities.

For peer review only

Identifying and diagnosing skin disorders.
What's the biggest challenge in this // What would help? What do you need?

For example:
The need for resources to support skin condition identification.
Specific skin conditions that you find difficult to identify (moles, insect bites, general rashes).

Additional prompts:
[is it about skin conditions? Is it about facilities/resources? Is it about knowledge/resources]

What's the biggest challenge in this – lack of knowledge, anxious public, number of conditions.
Adults / children?
Mild and very early presentation?
Time? And facilities?
Existing resources – adequacy? Appropriateness?
Training.
What do you currently do – examples of good practice.

Knowing when to refer someone to a GP.
What's the biggest challenge in this // What would help? What do you need?

For example:
Knowing which conditions should be referred to a GP (or when the severity of a condition requires referral).
Understanding how to refer / the need for more formal mechanism of referral.
Knowing when it is ok to offer advice/product and manage locally.

Additional prompts:
[is it about skin conditions? Is it about facilities/resources? Is it about knowledge/resources]

Are there specific situations/conditions where this is a particular problem?
What would discourage you from referring a patient?
Would having a referral pathway be useful similar to those between GPs and secondary care?
Is the reason for referral due to not knowing the diagnosis or not being able to treat the condition once diagnosed?

Skin of Colour.
What's the biggest challenge in this // What would help? What do you need?

For example:
Understanding the different presentation of skin conditions in skin of colour.
Knowing skin conditions which are distinct / more prevalent in skin of colour.

Additional prompts:
[is it about skin conditions? Is it about facilities/resources? Is it about knowledge/resources]

Did you receive any training specifically in diagnosing/treating conditions in skin of colour?
How have you learnt about treating conditions in patients with skin of colour?
Are there any conditions that you see more frequently in patients with skin of colour?

Disease specific concerns.
What's the biggest challenge in this // What would help? What do you need?

For example:
Recognising specific conditions which can / cannot be managed in community pharmacy.
Understanding how to support specific, chronic skin conditions.
The potential for dedicated roles in supporting specific conditions – trained staff, dedicated services.
Supporting members of the public with eczema self-management.

Additional prompts:
[is it about skin conditions? Is it about facilities/resources? Is it about knowledge/resources]

What long-term conditions do you see the most frequently?
What conditions do you see patients struggle to deal with?

For peer review only

What conditions do think a pharmacist could help to manage?
What role do you currently have in managing long-term conditions?
What role would you like to have with managing conditions both acute and long-term?

Product specific concerns.

For example:
Understanding concerns about the use of topical corticosteroids.
Understanding how to navigate the range of products available for skin conditions.
Understanding the benefit of products for different conditions and different severities. Knowing conditions for which you have no treatment.

Additional prompts:
[is it about skin conditions? Is it about facilities/resources? Is it about knowledge/resources]

What other medications do you think should be available OTC? Could be tested as OTC?
What are the common problems you have when selling TCS OTC? How do you think these problems should be managed?
What conditions are the most difficult to treat with the products that you have available?
How do you think

For peer review only

Public Pharmacy Dermatology Priority Setting Workshop

7pm WELCOME & INTRODUCTIONS FROM US.

- Thank-you for coming.
- Introductions from PL / JH.
- Purpose of the project.

The purpose of this project is to identify uncertainties that community pharmacists experience when they are faced with someone seeking help about a skin condition.

Our goal is to identify a small number of research projects which could be undertaken to support community pharmacists - these could be trialling new ways of working, developing information resources, understanding the challenges of seeing skin conditions in community pharmacy, testing treatments, etc.

- Purpose of this evening.

This evening we want to talk about your experiences of using a community pharmacy and discuss what roles you think pharmacists can play in the management of skin conditions. We want to know what types of projects you think need to be done that could help pharmacies to offer the best possible care to their patients.

- Rules of engagement.
- Split into two groups.
- Recording start.

7.10pm AROUND PARTICIPANTS – INTRODUCTIONS.

- name, where from, do you have any skin conditions/ experience of having skin conditions,

7.20pm

- Have you ever consulted a pharmacist/member of pharmacy staff regarding a skin condition? What were your experiences of using community pharmacy to treat skin conditions? Would you be as happy to see a pharmacist about a skin condition as you would about a cold? Do you have a long-term community pharmacist? Why do you go to that pharmacist?
    - Positive
    - Negative

- What would encourage you to use a community pharmacy for a skin condition?
    - Access
    - lack of GP appointments?
    - Familiarity (do you know your local pharmacist?)
    - See skin condition as a minor condition and not worthwhile bothering a GP (seeking reassurance from pharmacist that they should see a GP)

- What would discourage you from using a community pharmacy
    - Privacy (how would you feel about going into the consultation room?)
    - Having to speak to counter staff (would you feel comfortable asking to speak to a pharmacist)
    - Worried about lack of knowledge (would you feel any better knowing that the pharmacist had accessed specialist training in treating skin conditions?)

- When you have been to a community pharmacy which members of staff have you spoken to? How often do they bring the pharmacist into the conversation? Do you always get to see the pharmacist if you ask? Do you trust what the counter assistant tells you? Would you trust the pharmacist more?

For peer review only

- For those of you who have had skin conditions or have cared for people with skin conditions, at what point in your treatment do you think a pharmacist could have improved the quality of the care you/they received from the NHS as a whole.

  Please think about:
    - The initial diagnosis
    - The first time you were prescribed a medication
    - Each time you picked up a prescription from the pharmacy
    - When you purchased a treatment for a skin condition from the pharmacy
    - If the pharmacist referred you to someone else for treatment

- What role do you think the pharmacist should have? Should they diagnose your skin condition? Should they just treat your skin condition?
- What do you know about pharmacists training?

8pm short break

8.10pm

8.10pm RESEARCH TOPICS AND QUESTIONS.

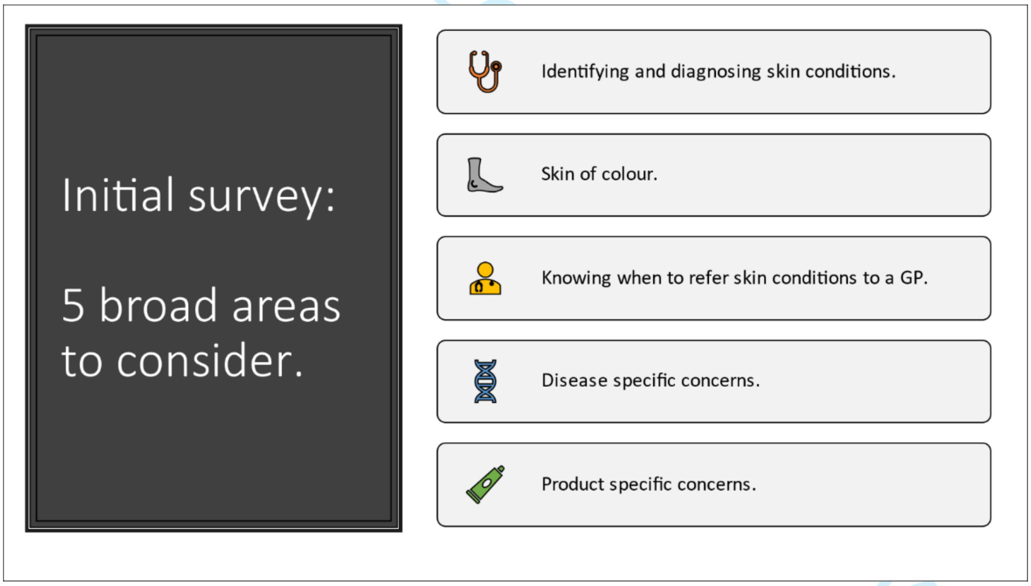

What do you think of this order?
Are you surprised?
Do you think the role of the pharmacist is to diagnose?
Or is their role just to tell you when you need to go to the GP?
Do you trust what a pharmacist says?
Do you think it would be useful if they could diagnose?

Identifying and diagnosing skin disorders.
What's the biggest challenge in this?
What would help? What do you need?

For example:
The need for resources to support skin condition identification.

Specific skin conditions that you find difficult to identify (moles, insect bites, general rashes).

Current use of photographs / websites / other resources?

What are you most likely to see? What is most likely to be difficult?

Additional prompts:
[is it about skin conditions? Is it about facilities/resources? Is it about knowledge/resources]

What's the biggest challenge in this – lack of knowledge, anxious public, number of conditions.
Adults / children?
Mild and very early presentation?
Time? And facilities?
Existing resources – adequacy? Appropriateness?
Training.
What do you currently do – examples of good practice.

Knowing when to refer someone to a GP.
What's the biggest challenge in this // What would help? What do you need?

For example:
Knowing which conditions should be referred to a GP (or when the severity of a condition requires referral).
Understanding how to refer / the need for more formal mechanism of referral.
Knowing when it is ok to offer advice/product and manage locally.

Knowing how long to wait?

What are you most likely to refer?

Additional prompts:
[is it about skin conditions? Is it about facilities/resources? Is it about knowledge/resources]

Are there specific situations/conditions where this is a particular problem?
What would discourage you from referring a patient?
Would having a referral pathway be useful similar to those between GPs and secondary care?
Is the reason for referral due to not knowing the diagnosis or not being able to treat the condition once diagnosed?

Disease specific concerns.
What's the biggest challenge in this // What would help? What do you need?

What conditions ? what challenges?

What's your role in this? What are you trying to achieve? [help GPs, …]

Do you want to do more?

Integrated services focused upon diseases?

For example:
Recognising specific conditions which can / cannot be managed in community pharmacy.
Understanding how to support specific, chronic skin conditions.
The potential for dedicated roles in supporting specific conditions – trained staff, dedicated services.
Supporting members of the public with eczema self-management.

Additional prompts:
[is it about skin conditions? Is it about facilities/resources? Is it about knowledge/resources]

What long-term conditions do you see the most frequently?
What conditions do you see patients struggle to deal with?
What conditions do think a pharmacist could help to manage?
What role do you currently have in managing long-term conditions?
What role would you like to have with managing conditions both acute and long-term?

Skin of Colour.
What's the biggest challenge in this // What would help? What do you need?

For peer review only

For example:
Understanding the different presentation of skin conditions in skin of colour.
Knowing skin conditions which are distinct / more prevalent in skin of colour.

Additional prompts:
[is it about skin conditions? Is it about facilities/resources? Is it about knowledge/resources]

Did you receive any training specifically in diagnosing/treating conditions in skin of colour?
How have you learnt about treating conditions in patients with skin of colour?
Are there any conditions that you see more frequently in patients with skin of colour?

Product specific concerns.

For example:
Understanding concerns about the use of topical corticosteroids.
Understanding how to navigate the range of products available for skin conditions.
Understanding the benefit of products for different conditions and different severities. Knowing conditions for which you have no treatment.

Additional prompts:
[is it about skin conditions? Is it about facilities/resources? Is it about knowledge/resources]

What other medications do you think should be available OTC? Could be tested as OTC?
What are the common problems you have when selling TCS OTC? How do you think these problems should be managed?
What conditions are the most difficult to treat with the products that you have available?
How do you think

8.40pm RANKING/SCORING.

8.50pm. CLOSING COMMENTS AND THANKS.

- vouchers.
- final workshop date and possibilities.

For peer review only

COREQ checklist

**Item number**		**Notes**
1	Which author/s conducted the interview or focus group?	All workshops and interviews were facilitated by both JH and PL.
2	What were the researcher's credentials? E.g. PhD, MD	JH has a Ph.D and MPharm. PL has a Ph.D
3	What was their occupation at the time of the study?	JH and PL work at the Centre of Evidence based Dermatology, School of Medicine, University of Nottingham.<br>JH is a registered pharmacist.
4	Was the researcher male or female?	N/A
5	What experience or training did the researcher have?	PL has extensive expertise in qualitative methods.<br><br>JH has completed several pieces of qualitative work examining pharmacist led services.
6	Was a relationship established prior to study commencement?	Researchers did not establish a relationship with participants prior to study commencement
7	What did the participants know about the researcher? e.g. personal goals, reasons for doing the research	Participants did not know that JH was a registered pharmacist. They were aware both researchers worked for the Centre of Evidence Dermatology.
8	What characteristics were reported about the inter viewer/facilitator? e.g. Bias, assumptions, reasons and interests in the research topic	We have acknowledged in the write-up that JH has a background as a pharmacist
9	What methodological orientation was stated to underpin the study? e.g. grounded theory, discourse analysis, ethnography, phenomenology, content analysis	This was exploratory, consultative research broadly informed by the James Lind Priority Setting Partnership approach.<br><br>See methods section, line 200, 206, 213, 216
10	How were participants selected? e.g. purposive, convenience, consecutive, snowball	See methods section line 165
11	How were participants approached? e.g. face-to-face, telephone, mail, email	Line 165,175
12	How many participants were in the study?	Table 1
13	How many people refused to participate or dropped out? Reasons?	Although the first focus groups were attended by 19 pharmacists, the final online consensus workshop was poorly attended by

For peer review only

		pharmacists and members of pharmacy staff. We had initially invited an equal number of pharmacists and pharmacy staff (12) and members of the public (13) but only 5/12 pharmacists and members of pharmacy staff, who were signed up prior to the meeting, attended the meeting.<br><br>Reasons (where provided) for nonattendance included sickness and that they were too busy.
14	Where was the data collected? e.g. home, clinic, workplace	Line 203
15	Was anyone else present besides the participants and researchers?	No
16	What are the important characteristics of the sample? e.g. demographic data, date	We did not collect any demographic data.
17	Were questions, prompts, guides provided by the authors? Was it pilot tested?	Workshops used the findings of earlier stages of the research as their focus.<br><br>Data analysis and progress summaries were considered by the wider author group and PPI representatives prior to the workshops.
18	Were repeat inter views carried out? If yes, how many?	N/A
19	Did the research use audio or visual recording to collect the data?	Line 211
20	Were field notes made during and/or after the interview or focus group?	No fieldnotes were made during or after data collection.
21	What was the duration of the inter views or focus group?	Line 250
22	Was data saturation discussed?	Data saturation was not a concept applied to this research, rather data collection
23	Were transcripts returned to participants for comment and/or correction?	No
24	How many data coders coded the data?	PL and JH coded the data. Other authors reviewed coded data and confirmed appropriateness of interpretations.
25	Did authors provide a description of the coding tree?	Yes Table 2

For peer review only

26	Were themes identified in advance or derived from the data?	Initial themes were derived from the inductive content analysis of the online survey. Themes were refined following the exploratory workshops.
27	What software, if applicable, was used to manage the data?	NVivo ver 12 was used to handle research data.
28	Did participants provide feedback on the findings?	No
29	Were participant quotations presented to illustrate the themes/findings? Was each quotation identified? e.g. participant number	Yes, identified by participant number and profession and by workshop number
30	Was there consistency between the data presented and the findings?	Yes findings were based on an analytic process that was iterative.
31	Were major themes clearly presented in the findings?	Yes in the form of the 10 questions
32	Is there a description of diverse cases or discussion of minor themes?	Yes where applicable in the text

For peer review only

Introduction

The responses from two open questions from the Aston Pharmacy Dermatology Survey have been analysed.

We have:

- Identified the top 20 words from the two open questions; the questions concerned are the “challenge” question and the “research priority” question.
- Created word trees from the most common words identified, then used thematic analysis of the word trees to draw up the long list of questions.

Please see below for an example of the analyses mentioned above.

For peer review only

Challenge word analysis

Challenge question: Please list at least one and up to two of the most challenging aspects of treating or managing patients presenting with skin conditions. For example: "How can community pharmacy best support safe use of topical corticosteroids?" or "How do we know when to refer someone with a mole?"

Number of challenge word responses=348

Top 20 words from the challenge question

Word	Count	Similar words
**refer**	44	refer, referal, referred, referring
**rash**	39	rash, rashes
**products**	26	product, products
**differential**	25	differential, differentials, differentiate, differentiating
**know**	24	know, knowing
**diagnosis**	22	diagnosis
**different**	21	difference, differences, different, differently
**steroid**	19	steroid, steroids
**need**	16	need, needed, needing, needs
**support**	16	support, supporting
**treatment**	16	treatment, treatments
**creams**	15	cream, creams
**infected**	15	infected, infection, infections
**time**	15	time, timely, times
**limited**	14	limitations, limited, limits
**difficult**	14	difficult
**emollients**	14	emollient, emollients
**identify**	14	identify, identifying
**treat**	14	treat, treated, treating
**mole**	14	mole, moles

For peer review only

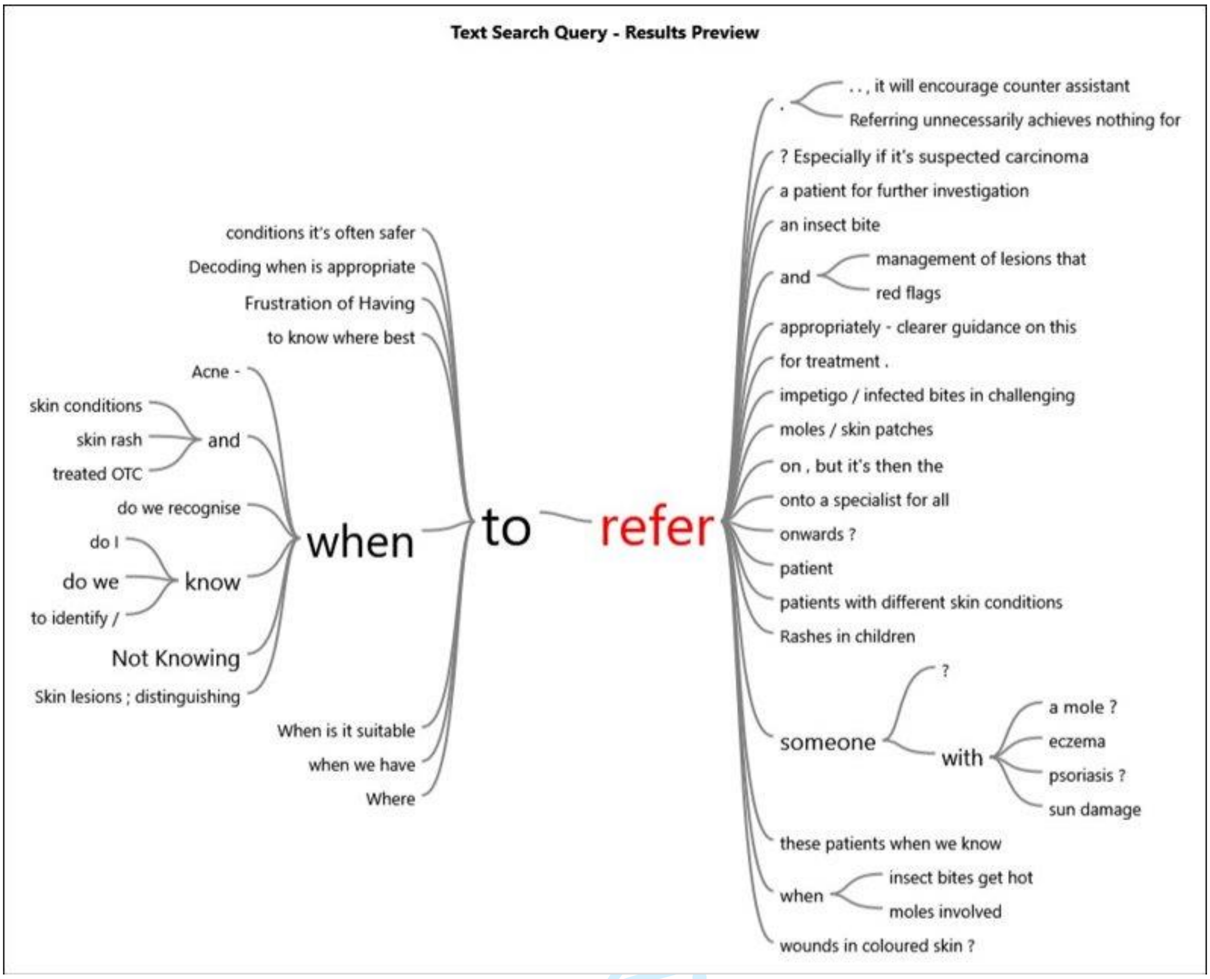
Text Search Query - Results Preview
conditions it's often safer
Decoding when is appropriate
Frustration of Having
to know where best
Acne -
skin conditions
skin rash
treated OTC
and
do we recognise
do I
do we
to identify /
know
Not Knowing
Skin lesions ; distinguishing
when
When is it suitable
when we have
Where
to
refer
. . , it will encourage counter assistant
Referring unnecessarily achieves nothing for
? Especially if it's suspected carcinoma
a patient for further investigation
an insect bite
and
management of lesions that
red flags
appropriately - clearer guidance on this
for treatment .
impetigo / infected bites in challenging
moles / skin patches
on , but it's then the
onto a specialist for all
onwards ?
patient
patients with different skin conditions
Rashes in children
someone
?
with
a mole ?
eczema
psoriasis ?
sun damage
these patients when we know
when
insect bites get hot
moles involved
wounds in coloured skin ?

Research priority analysis question

Please list at least one and up to three of your top research priorities (e.g.what do we need to know more about) in relation to the role of community pharmacy in skincare (consider both advice/counter prescribing and advice on prescription medications).

Number of research priority responses - 522

Top 20 words – research priority questions

Word	Count	Similar words
**treatment**	47	treatment, treatments
**different**	32	difference, different, differing
**products**	31	product, products
**need**	28	need, needed, needing, needs
**creams**	24	cream, creams
**rashes**	22	rash, rashes
**care**	21	care
**steroid**	21	steroid, steroidal, steroids
**pharmacists**	20	pharmacist, pharmacists
**eczema**	19	eczema
**psoriasis**	18	psoriasis
**training**	18	train, trained, training
**prescribing**	17	prescribe, prescribed, prescriber, prescribers, prescribing
**diagnosis**	16	diagnosis
**information**	16	inform, information
**effects**	15	effecting, effective, effectively, effectiveness, effects
**acne**	15	acne
**advice**	15	advice
**infections**	15	infected, infection, infections
**referral**	15	referral

For peer review only

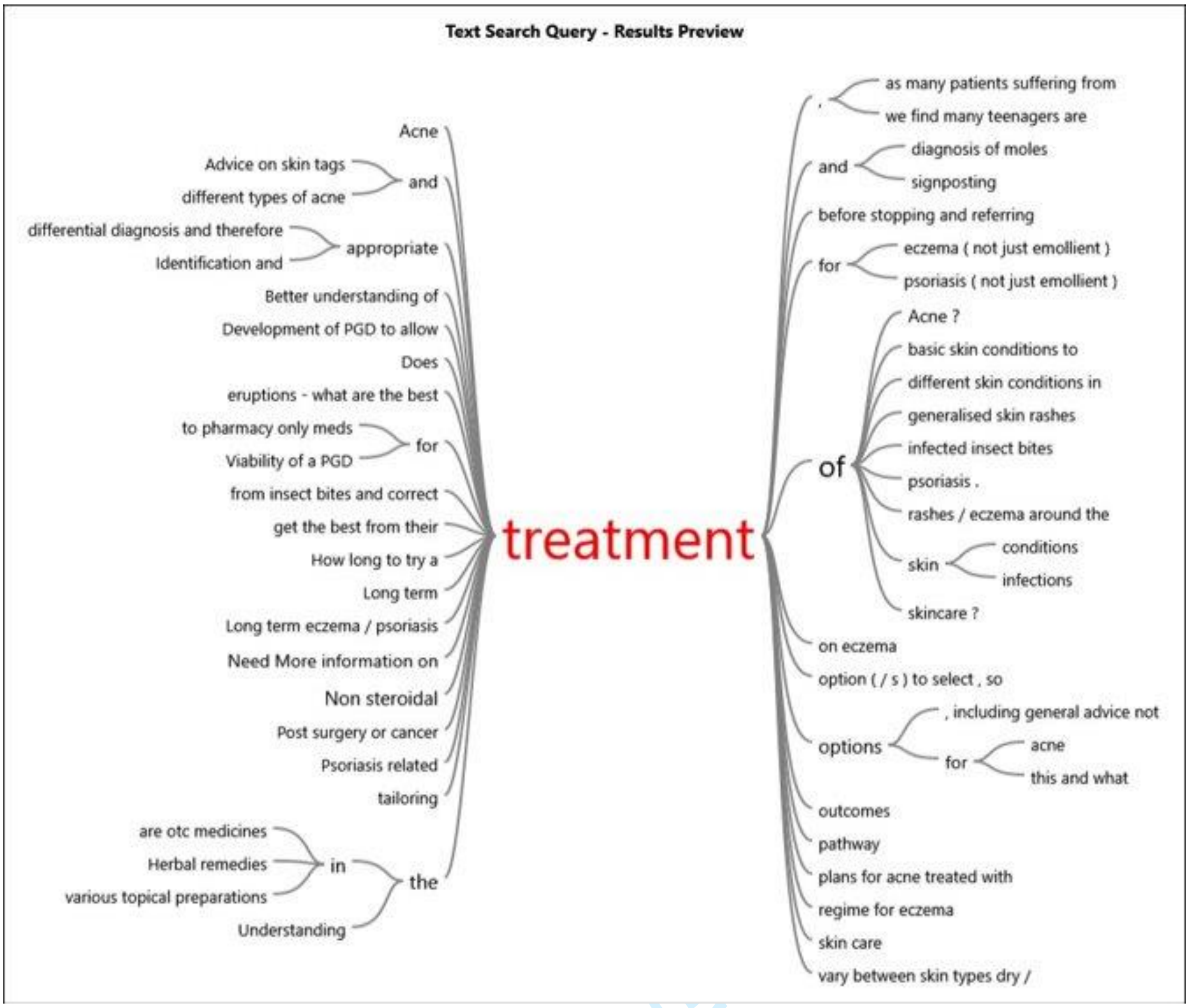
Text Search Query - Results Preview
treatment
Acne
Advice on skin tags
different types of acne
and
differential diagnosis and therefore
Identification and
appropriate
Better understanding of
Development of PGD to allow
Does
eruptions - what are the best
to pharmacy only meds
Viability of a PGD
for
from insect bites and correct
get the best from their
How long to try a
Long term
Long term eczema / psoriasis
Need More information on
Non steroidal
Post surgery or cancer
Psoriasis related
tailoring
are otc medicines
Herbal remedies
various topical preparations
in
Understanding
the
,
as many patients suffering from
we find many teenagers are
and
diagnosis of moles
signposting
before stopping and referring
for
eczema (not just emollient)
psoriasis (not just emollient)
of
Acne ?
basic skin conditions to
different skin conditions in
generalised skin rashes
infected insect bites
psoriasis .
rashes / eczema around the
skin
conditions
infections
skincare ?
on eczema
option (/ s) to select , so
options
, including general advice not
for
acne
this and what
outcomes
pathway
plans for acne treated with
regime for eczema
skin care
vary between skin types dry /

Further quotes to support research questions derived from Themes 1 and 2 – Resources to support Identifying and diagnosing skin conditions and resources to support Identifying and diagnosing skin conditions in skin of colour

Resources to help the Identification of skin conditions within community pharmacies	*"Yeah, if someone says they have dry eyes, you don't recognize patterns. You just accept you have dry eyes" Pharmacist*<br><br>*"I think a lot of dermatology things sometimes we've only seen them in pictures, so not actually in real life. And obviously everyone's different and they don't necessarily present how we've seen them in pictures so you're kind of making a judgment call whether it's close enough to the picture or whether it's something completely different that they're presenting with." Pharmacist*
Resources to help the Identification of skin conditions in skin of colour within community pharmacies	*"OK, I use Google images quite a bit for this because they're like what does it look like in a darker skin tones it and Google Google's great. But you know, like, I I don't know if this is like, you should say, oh, this is looking better. I can't say that in a different colour skin tone." Pharmacist*<br><br>*"Yeah, my worst fear is missing a red flag in like a darker skin tone like cancer. You're more likely to know what it looks like on Caucasian skin, but on dark skin I just worry that I missed the red flag." Pharmacist*

For peer review only

Further quotes to support the research question derived from Theme 3 - Resources to support referral

Resources to support referral of skin conditions	*"I probably wouldn't have gone to A&E had I not sort advice from them first, because I wouldn't have wanted to bother A&E. And so you almost feel like a bit better about going to and when they're really busy about what is just this little thing on your neck, because actually you can say, well, I've just been into boots and I was actually advised to come here and that makes you feel a little bit sort of that you've already gone and sought a bit of help first." Patient*<br><br>*"it would be really nice to think if the pharmacist really wanted to be useful and escalate the problem they knew where to send it to, because GPS also generally don't know that much about [skin] because there's thousands of different skin conditions" Patient*<br><br>*"My fear is we go give out antibiotics for that, or impetigo like give the Fucidin cream out for that and we actually delay treatment for something that maybe is a bit more serious." Pharmacist*<br><br>*"It is sometimes difficult even after all this time it is difficult to determine exactly or to have confidence in what you think a particular rash might be and how infectious it is or is not" Pharmacist*<br><br>*"I think the with children with any sort of, any really well, anytime a child comes to you in a pharmacy, I think you just. You feel. More pressure to make the right decision because. You refer just in case it is something more serious because you've got that doubt in the back of your mind." Pharmacist*<br><br>*"Yes, I struggled to refer things to the GP and just cos our GP is getting massive very quickly. We've got a lot of new builds in our area and so they are quite snowed under and generally I think any appointment time is about four weeks and so it is a struggle to refer and normally we just say if you mentioned that you've been to us because we are opposite, we are, we share a gangway so they are quite nice about it, but it's a struggle to say this is serious enough to be seen tomorrow." Pharmacist*<br><br>*"The idea of suggesting to GP that they sort of take on more of a training role or sort of a feedback system with a community pharmacist might not land very well, but in an ideal world I think it's a great a great idea. I would. I would love to have. So[me] two way dialogue, you know, even if if they if they send pictures through, like our district nurses send pictures of patients all the time of the of a wound on their leg and say do you think this needs antibiotics?" GP*

For peer review only

Further quotes to support the research question derived from Theme 4- Working with other healthcare professionals

**Working with other healthcare professionals**	
Initial management of skin conditions	*"Because what I usually tell patients is in this climate if you're not going to get into the GPS for a few weeks, you may as well try one week of hydrocortisone and this emollient and then you have that information to tell the prescriber. If it's better, brilliant if it's worse, at least you won't be sent away with hydrocortisone and an emollient, and you'll already be one step ahead, but I just don't know whether that's the most useful action, so I think just more of a conversation." Pharmacist*<br><br>*"I think going from Community pharmacy to now working alongside GPs when I do have a a concern about a skin condition or some sort of query, you find that a lot of them actually just use trial and error with different prescription only medications and creams. So they'll just say try this for a week If it doesn't work then come back and a lot of it, even they're not 100% sure." Pharmacist*
Roles in management of long-term skin conditions	*"I I think I think if people have got, […] already got a diagnosis. I think that [upskilling pharmacists to provide counselling] would be really helpful because you know follow up appointments are getting more and more difficult. I think everything's changed so much in the last 20 years because we've now got biologics. So in in secondary care then we're mainly focusing on patients who have had the systemic medications at the topicals, the systemics." Specialist dermatology nurse*<br><br>*"So in all the years that I've been having these meds I've never been offered any kind of advice except for once. When the pharmacist said don't forget to keep these in the fridge because they don't like lots of hot hot temperature." Patient*

For peer review only

Further quotes to support the research questions derived from Theme 5 - Resources to support the management of skin conditions and improving access to medications

Management of skin conditions	*I just always find it difficult with eczema, just the amount of emollients, and it's what emollients to recommend and what circumstance. Pharmacist*
Providing a wider range of treatments	*"I find it difficult to treat over the counter because I feel quite limited in what we're what we're licensed to sell and for what and for use for, like on on the areas of the body" Pharmacy dispenser*<br><br>*"I can't tell you how many referrals I've made for impetigo because there's just nothing to offer" Pharmacist*
Access to topical corticosteroids	*"And as someone mentioned, you have to be quite careful of what you say to them in order to get it [TCS]. " Patients*<br><br>*"I might treat something off license and and pharmacists are very much by the book. But in dermatology, we can't always do things by the book and we have to use things off licence because sometimes there's just nowhere else to go. I mean, you know, I for years, I I had a paediatric eczema clinic. And I just felt so sorry for those parents who, even with the GP, found it difficult to to get the medication that they needed if their children's skin was flaring and they had quite severe flares and they had to wait to come and see us and we only had it clinic once once a week." Specialist dermatology nurse*<br><br>*"[the patient said] I've forgotten my cream and they wanted to buy hydrocortisone cream and they said I've got eczema on my face, and you simply can't sell it. And and and you get into it. So so in many respects you you've got kind of silly restrictions like that which actually impede your ability to practice properly because you think because you effectively look a bit stupid because, well, the doctor gives it to me and you're saying you can't sell it to me and you think what is all that about?" Pharmacist*<br><br>*"I'm not concerned about pharmacists supplying the items inappropriately, but it's the whole over the counter consultation because this my staff know that the face is a no go zone for hydrocortisone. So if somebody was requesting it for the face, that's a hard kind of stop for them to come and grab me to have a conversation with the patient about this. Whereas if that licensing was removed, you may run* the *risk of just missing these patients throughout the whole pharmacy team." Pharmacist*

For peer review only

Further quotes to support Research Question from Theme 6 - Current involvement of pharmacists and the known benefits of the involvement of community pharmacists in the identification and management of skin conditions

Assessment of rising demand	*"And I see such a broad range [..] I just see a bit of everything all the time" Pharmacist*<br><br>*"but I do get a lot of people coming in children with children. All sorts of skin conditions. As you said ringworm and eczema and slap cheek." Pharmacist*<br><br>*"I think it has [consultations for skin disease] increased, but I was still seeing quite a few skin conditions, so people coming in asking before COVID as well asking what it might be and anything [I] could recommend." Pharmacist*<br><br>*"I think it will, I think it will stay [the increased demand], UM I, I think. [It's] the ease of access you know? And because people are not getting appointments with GP" Pharmacist*
Variation in service provisions	*"I think the main ailments aspect needs to be stretched. To cover all areas of the country to make it easier for pharmacies in areas that don't have it. Because some of the things that we can't assist with are ridiculous." Pharmacy dispenser*<br><br>*"[the geographical area] has some PGDs that allow for supply, for example, hydrocortisone on the face under PGD" Pharmacist*<br><br>*"You know, and if I've got the choice of trying to get an appointment to see a GP. And then they'll give me a prescription, and then because I've got a prepayment, I don't have to pay for it or going to a chemist and then charging me £9 for a bottle of Double Base Ointment. The logic says you go to the GP first. You've just saved yourself. You know, a tenner" Patient*<br><br>*"I'm not a massive fan of the CPCS thing yet, cause UM our area doesn't do much minor ailments and they're expecting things for free. And I'm, they're like, no, I can't. I can't give you that you're gonna have to. You're gonna have to pay." Pharmacist*<br><br>*" How well do you trust somebody's clinical judgment against their commercial need?" Patient*

For peer review only

Further quotes to support Research Question 9. and 10. Competency of community pharmacists in managing skin conditions and awareness of the public of the skills of community pharmacists with regards to the identification and management of skin conditions.

Pharmacist competency in the identification and treatment of skin conditions	*"I would guess a certificate on the wall […] go to my optician and he's got all his different specialities on on the wall. So something just with simple as that and I guess they would have to be trained by a consultant dermatologist or at least somebody with many years of experience at spotting all kinds of different conditions." Patient*<br><br>*"I think one I don't know what community pharmacists know, and I think that's part of the problem. We work very closely with community pharmacists in some ways, but we're very, very separate in others, so we phone them up about medication queries and they phone us all the time. But actually knowing what each other's skills are is is quite difficult." GP*<br><br>*"But what I do see is what they've attempted to treat and then comes to us anyway, so I see quite a lot of misdiagnoses from community pharmacies, so a lot of insect bites that look very red and inflamed are sent to us because they think it's infected […] we see lots of sort of cold sore sent to us as query impetigo needing antibiotics, lots of eczema that's just red and inflamed might be infected and gets sent to us and it's not, it just needs eczema treatment so I think. I I see some fairly basic areas of of lacking in knowledge […] but what I don't see are the ones that they have successfully treated and and and sent away so I don't know what sort of ratio we're talking here." GP*
Raising awareness of pharmacy skin services	*"I've had various sort of short term skin conditions and pharmacies have always been my sort of first port of call. It [has] always been good experiences" Patient*<br><br>*"I never knew the pharmacy that they could advise you on skin you know problems, I never knew that. Because I thought they dealt dealt with drugs only and they all seemed very busy. So I'd like to know how the pharmacist can help with skin conditions as well?" Patient*<br><br>*"This minor ailment scheme and for that it needs the patient consent, so we need them to reply to the text message or we need to speak to them on the phone to get their consent and then make the referral, which is a very quick process, but it's all quite clunky and it doesn't always have the patient buy in because they, sometimes incorrectly, value the GP's skills more than the community pharmacists skills, so there needs to be a degree of sort of selling of this and patient education" GP*

For peer review only

N.B. In the notes below, where we refer to community pharmacists, we include any appropriately qualified member of the community pharmacy team.

Question	Explanatory notes
1. What are the known benefits of community pharmacy involvement in the identification and management of skin conditions?	• This could be about establishing what is already known about community pharmacy involvement in the management of skin conditions.<br>• This could be important research which underpins the development of new services or resources.<br>• In our focus groups, participants described several different activities where community pharmacists have helped people with skin conditions. Some examples are these activities are; diagnosing, supporting self-management, recommending the use of products, and participating in dedicated medicines/illness schemes. Knowing which activities have evidence of being beneficial could support future provision.
2. In what ways are community pharmacists currently involved in the identification and management of skin conditions?	• This could be about developing a more detailed understanding about what currently happens in community pharmacy.<br>• It could include assessing levels of demand, mapping different types of activity, and identifying novel ways of working.<br>• In our focus groups it was apparent that services are developing differently across the country and that people were not aware of all that is currently happening in different areas.
3. How competent are community pharmacists in the identification and management of skin conditions?	• This could involve assessing the knowledge and skills of community pharmacists and other members of the pharmacy team.<br>• In our focus groups pharmacists described different levels of confidence and competence in addressing skin conditions. Members of the public were uncertain about how qualified community pharmacists are to manage skin conditions.
4. Would dedicated resources improve the **identification of skin conditions** by community pharmacists?	• This could involve developing and/or testing resources which support community pharmacists in identifying skin conditions. Resources might be online resources, training programmes etc.<br>• In our focus groups pharmacists described an absence of resources that they are in confident in using to identify skin conditions, they said that high quality resources could be useful in identifying skin conditions.
5. Would dedicated resources improve the **identification of skin conditions** in **skin of colour** by community pharmacists?	• This could involve developing and/or testing resources which support community pharmacists in identifying skin conditions in skin of colour.<br>• In our focus groups pharmacists described that identifying skin conditions in skin of colour is an additional challenge due to the different ways that skin conditions can look, and also because most current training/resources present images of white skin.
6. Would dedicated resources support community pharmacists in the **management** of skin conditions?	• This could involve developing and/or testing resources which support community pharmacists in managing skin conditions.<br>• In our focus groups pharmacists described being unsure about when they might take an active role in skin condition management. They also wanted

For peer review only

Question	Explanatory notes
	evidence-based information about what support and products they can provide.<br><br>• This might focus upon specific conditions (eczema, psoriasis, insect bites, etc.) or could be for more generic conditions (a rash, dry skin, etc.).
7. Would dedicated resources support community pharmacists to **effectively refer skin conditions** that require urgent or more specialist attention?	• This could involve developing and/or testing resources which support community pharmacists in referring skin conditions to other sources of healthcare (e.g. emergency care, general practice, etc.).<br><br>• It might include developing referral pathways and new services for specific serious skin conditions.<br><br>• In our focus groups pharmacists described feeling uncertain about when to take this sort of action; members of the public recognised that sometimes they wanted a pharmacist to 'tell them to go to the doctor'.
8. Could a wider range of products and treatments for skin conditions be made available via community pharmacy?	• This might include considering treatments which are restricted due to legal reasons (e.g. can only be supplied against a prescription) or economic reasons (e.g. where price might inhibit purchase).<br><br>• In our focus groups both pharmacists and members of the public described frustration that certain products are restricted and cannot be provided by a pharmacist.
9. What could be done to raise awareness of the skills that community pharmacists have with regards to the identification and management of skin conditions?	• This could involve local or national campaigns to raise awareness about community pharmacy as a healthcare resource. It might focus upon how community pharmacy services are understood by members of the public.<br><br>• In our focus groups some members of the public were not aware that community pharmacists are able to provide healthcare advice about skin conditions. Some members of the public were concerned that commercial concerns might be a priority for pharmacists, i.e. selling products might be more important than providing appropriate advice.
10. How can community pharmacists work most effectively with other healthcare professionals in the identification and management of skin disease?	• This might involve new integrated services for the management of skin conditions that include community pharmacists and other healthcare providers (e.g. GPs, nurses, dermatologists). It could include the use of pharmacist independent prescribers or pharmacists with additional training in dermatology.<br><br>• In our focus groups pharmacists reported working in isolation with little or no feedback about the advice and services that they offer. They also indicated that it is sometimes difficult to contact or engage with other healthcare providers about skin conditions.

For peer review only
